# Mechanisms of *ag85a/b* DNA vaccine conferred immunotherapy and recovery from *Mycobacterium tuberculosis*‐induced injury

**DOI:** 10.1002/iid3.854

**Published:** 2023-05-16

**Authors:** Nan Wang, Yan Liang, Qianqian Ma, Jie Mi, Yong Xue, Yourong Yang, Lan Wang, Xueqiong Wu

**Affiliations:** ^1^ Tuberculosis Prevention and Control Key Laboratory, Beijing Key Laboratory of New Techniques of Tuberculosis Diagnosis and Treatment, Senior Department of Tuberculosis, The Eighth Medical Center of PLA General Hospital PLA General Hospital Beijing China

**Keywords:** *ag85a/b* DNA, DNA vaccine, immunotherapy, *Mycobacterium tuberculosis*, transcriptome

## Abstract

Our previous research developed a novel tuberculosis (TB) DNA vaccine *ag85a/b* that showed a significant therapeutic effect on the mouse tuberculosis model by intramuscular injection (IM) and electroporation (EP). However, the action mechanisms between these two vaccine immunization methods remain unclear. In a previous study, 96 *Mycobacterium tuberculosis* (MTB) H_37_Rv‐infected BALB/c mice were treated with phosphate‐buffered saline, 10, 50, 100, and 200 μg *ag85a/b* DNA vaccine delivered by IM and EP three times at 2‐week intervals, respectively. In this study, peripheral blood mononuclear cells (PBMCs) from three mice in each group were isolated to extract total RNA. The gene expression profiles were analyzed using gene microarray technology to obtain differentially expressed (DE) genes. Finally, DE genes were validated by real‐time reverse transcription‐quantitive polymerase chain reaction and the GEO database. After MTB infection, most of the upregulated DE genes were related to the digestion and absorption of nutrients or neuroendocrine (such as Iapp, Scg2, Chga, Amy2a5), and most of the downregulated DE genes were related to cellular structural and functional proteins, especially the structure and function proteins of the alveolar epithelial cell (such as Sftpc, Sftpd, Pdpn). Most of the abnormally upregulated or downregulated DE genes in the TB model group were recovered in the 100 and 200 μg *ag85a/b* DNA IM groups and four DNA EP groups. The pancreatic secretion pathway downregulated and the Rap1 signal pathway upregulated had particularly significant changes during the immunotherapy of the *ag85a/b* DNA vaccine on the mouse TB model. The action targets and mechanisms of IM and EP are highly consistent. Tuberculosis infection causes rapid catabolism and slow anabolism in mice. For the first time, we found that the effective dose of the *ag85a/b* DNA vaccine immunized whether by IM or EP could significantly up‐regulate immune‐related pathways and recover the metabolic disorder and the injury caused by MTB.

## Graphical abstract

Our research explored the immunotherapeutic mechanism of tuberculosis *ag85a/b* DNA vaccine by electroporation. The results showed the *ag85a/b* DNA vaccine has a significant immunotherapeutic effect on gene expression disturbance of tuberculosis‐infected mice.

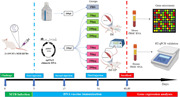

## 1 INTRODUCTION

Tuberculosis (TB) is an infectious disease caused by *Mycobacterium tuberculosis* (MTB) invasion, which is one of the main causes of death from infectious diseases and the leading cause of death from drug resistance.^1^ According to the Global Tuberculosis Report 2022 published by World Health Organization (WHO), there were about 10.6 million new cases, 1.6 million dead cases of TB, and 450,000 multidrug‐resistant/rifampicin‐resistant TB (MDR/RR‐TB) cases worldwide in 2021.^1^ It can be seen that TB remains a major infectious disease threatening human health. The diagnosis and treatment of MDR/RR‐TB is a major clinical problem. The global pandemic COVID‐19 has also brought great challenges to the prevention and control of TB.^2^

TB is not only a bacterial infectious disease but also an immune disease.^3^ The occurrence and development of TB are closely related to immunodeficiency,^4^, ^5^ imbalance of Th1/Th2 immune response,^6^ and hypoimmunity.^7^, ^8^, ^9^ Chemotherapy only with anti‐TB drugs needs 6–9 months or even longer to kill the vast majority of MTB in the lesion.^10^, ^11^ However, there may still be a small amount of persisting MTB in vivo, especially in macrophages, which is difficult to remove and becomes a “time bomb” for TB recurrence.^12^ Anti‐TB immunoadjuvant therapy with immunomodulators has great potential in preventing latent MTB reactivation and treating active TB patients.^13^ It can correct low or abnormal immune function, inhibit the adverse immune response and inflammatory injury, and improve the immune function and curative effect. In recent years, immunoadjuvant therapy for TB has made great progress. Some immunomodulators have entered clinical trials or been marketed, mainly including immunoactive substances, immunotherapeutic vaccines, chemical agents,^14^ traditional Chinese medicine, and cell therapy.^13^

TB immunotherapeutic vaccine is used to regulate or selectively induce the potential of the immune system of MTB‐infected people, to achieve the purpose of suppressing immune damage, recovering immune balance, improving immunity, and inhibiting or killing MTB in vivo.^15^ It is mainly used to prevent individuals with latent tuberculosis infection from turning into active TB or help active TB patients recover faster. Using a vaccine for the prophylactic treatment of high‐risk populations with MTB infection is simple, convenient, economical, and has few side effects.^16^ At present, there are the following types of TB therapeutic vaccines: (1) Inactivated vaccines: Of the TB inactivated vaccines prepared from nontuberculous *Mycobacteria*, *Vaccae* (Prepared from inactivated *Mycobacterium vaccae*)^17^ and Utilins^18^ (prepared from inactivated *Mycobacterium phlei*) have new drug certificates in China. DAR‐901^18^ (SRL172, prepared from inactivated *Mycobacterium kyogaense*) and MIP^19^ (prepared from inactivated *Mycobacterium indicus pranii*) have entered clinical trials. (2) Subunit vaccine: Of the subunit vaccines prepared from some cell components of the MTB complex, BCG polysaccharide and nucleic acid injection (trade name Siqikang) has obtained a new drug certificate in China^18^; RUTI (prepared from MTB H37Rv cultured under low oxygen, low pH and low nutrient conditions by crushing, detoxification and then embedding in liposomes),^18^ and four recombinant protein vaccines (M72/AS01E, H56/IC31, ID93/GLA‐SE, and AEC/BC02) have entered phase I or II clinical trials^20^, ^21^, ^22^; (3) DNA vaccine: Of the DNA vaccines constructed from the genes encoding MTB antigen and eukaryotic expression vectors, only Korean GX‐70 (composed of four MTB antigen plasmids and Flt3 ligand) has obtained the approval for phase I clinical trial (ClinicalTrials.gov Identifier: NCT03159975), but this study has been withdrawn because of unconfirmed research expenses. Although no TB DNA vaccine has entered the clinical trial status, many results of animal experiments proved that DNA vaccine could provide remarkable protective efficacy and strong therapeutic effect on mouse TB models.^23^, ^24^, ^25^

MTB Ag85A and Ag85B are secreted proteins and antigens recognized by host innate immune cells at an early stage, with good immunogenicity. However, the adaptive immune response in the mouse lungs arrests the proliferation of MTB and results in a 10 to 20‐fold reduction in the messenger RNA (mRNA) expression of the secreted Ag85 complex.^26^, ^27^ The downregulation of gene expression significantly reduces the frequency of Ag85A/Ag85B‐specific CD4^+^ effector T cells activated during the MTB infection. Therefore, the ag85 antigens have become popular candidate targets for developing new TB vaccines.^22^

Our previous studies have demonstrated that the *ag85a/b* chimeric DNA vaccine could induce significant Th1 and CTL cellular immune responses, relieve lung tissue lesions, reduce the bacterial load in organs, and have a significant treatment effect on MTB‐infected mice.^28^ To solve the problem of relatively low immunogenicity of DNA vaccines and the need for very high doses in large animal and human clinical trials,^29^, ^30^ our team used electroporation (EP) technology to deliver different doses of MTB *ag85a/b* chimeric DNA vaccine and compared their immunotherapeutic effect with traditional intramuscular injection (IM). The results showed that the CD4^+^IFN‐γ^+^T cells% in whole blood from 200 µg DNA IM group and 4 DNA EP groups increased significantly (*p* < .05). The CD4^+^CD25^+^Treg cells% decreased significantly in all DNA vaccine groups (*p* < .01). These results proved that EP immunization can improve the immunogenicity of low‐dose DNA vaccines and reduce the amount of plasmid DNA used. The therapeutic effect of the 50 μg DNA EP group on the mouse TB model had no significant difference with the 100 μg DNA IM group. They all could significantly reduce the bacterial load of the lung and spleen, and lung lesion areas, resulting in a good immunotherapeutic effect.^31^

At present, the pathogenesis of MTB and the interaction between MTB and host have not been fully elucidated, which is a challenge to the research and development of an effective vaccine for TB. After the DNA vaccine is expressed in vivo, the correlation and mechanism of its inducing protective immunity have also not been completely determined. First, we need to understand the interaction between the DNA vaccine and the host, the key anti‐TB targets of the proteins expressed by the DNA vaccine, and the body's multiple anti‐TB systems regulated by the DNA vaccine. Second, it is necessary to understand in depth the protective immune response of DNA vaccine in TB treatment, determine whether it can repair the pathological damage caused by MTB infection, help to inhibit and eliminate MTB, and find out what indexes are helpful to evaluate the effectiveness of the new TB vaccine. Third, we need to understand the possibility of DNA vaccine inducing pathological immune responses to determine the risk of possible adverse reactions to the vaccine.

In recent years, the development of the frontier disciplines of systems biology has provided a powerful tool for the study of the pharmacological mechanism of vaccines.^32^, ^33^, ^34^ Therefore, this study used gene chip technology to obtain the gene expression profiles of experimental animals, and used bioinformatics methods to identify the differential expression levels of genes from mouse peripheral blood mononuclear cells (PBMCs) before and after MTB infection and before and after *ag85a/b* DNA vaccine treatment. This is the first attempt to analyze the pathogenic targets of MTB and the therapeutic targets of *ag85a/b* DNA vaccine at the level of gene transcription, and then to elaborate the molecular mechanism of DNA vaccine in regulating disease network and playing the role of anti‐TB by combining pathway analysis and functional analysis, and so forth. At the same time, we analyzed whether there are differences in the effective dose, action target, and action mechanism of the two DNA immunization methods by comparing the differentially expressed (DE) genes before and after immunotherapy with different doses of *ag85a/b* DNA IM and EP. In addition, the immune characteristics of the *ag85a/b* DNA vaccine were verified through animal experiments, and the protective immune response of the vaccine was analyzed by comparing the therapeutic effects. Finally, the expression levels of three MTB pathogenic target genes found in this study were verified in TB patients by real‐time reverse transcription‐quantitive polymerase chain reaction (RT‐qPCR) to determine the reliability of the gene expression profiling results. In addition, we downloaded gene expression data sets from the GEO database to compare with our expression profile results. The same MTB pathogenic target genes and therapeutic target genes were screened to verify our expression profile results.

## 2 MATERIALS AND METHODS

**Figure 1:**
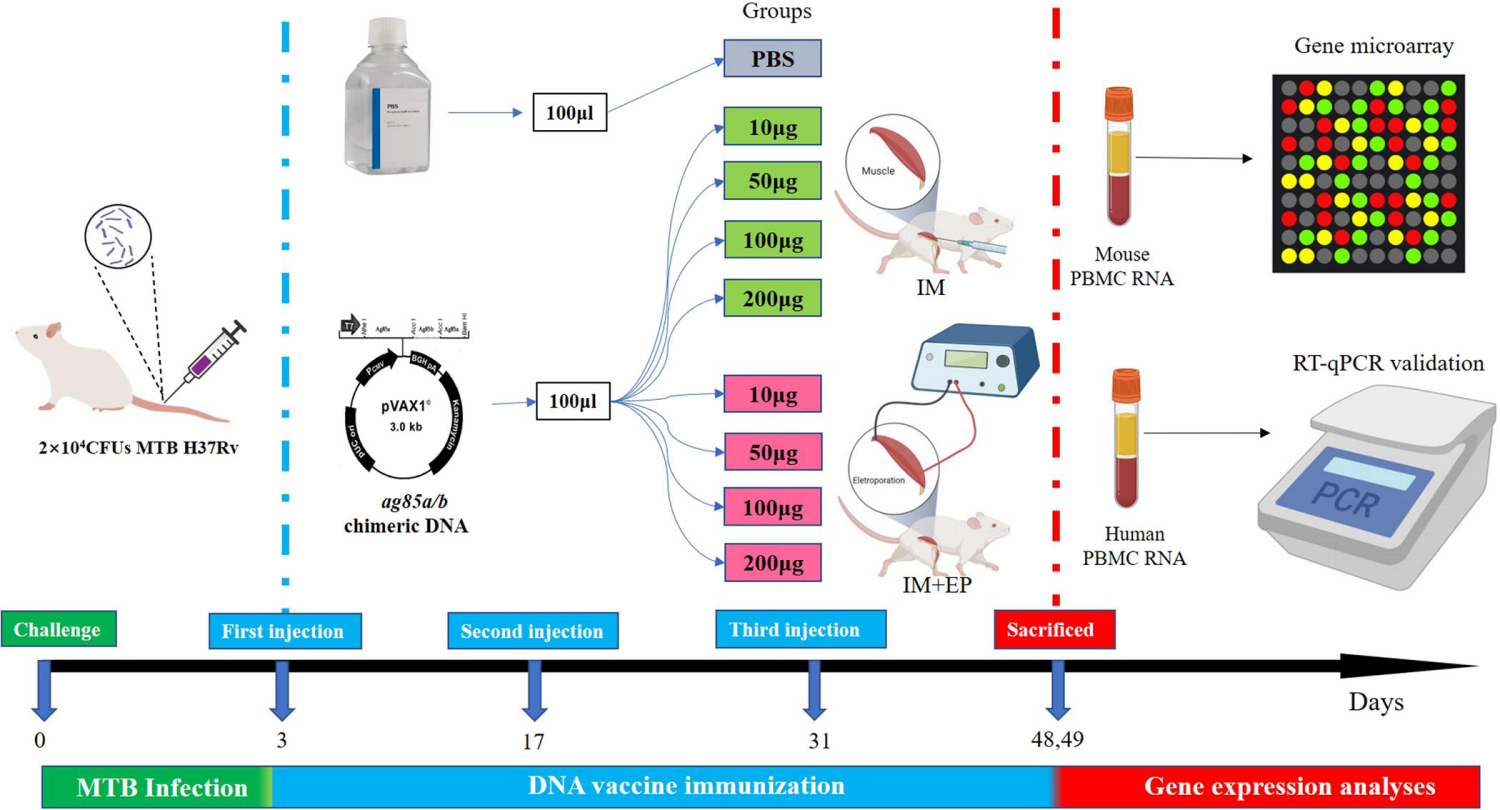
96 BALB/c mice were challenged with MTB H37Rv at 0 days, divided into nine groups, and immunized respectively with PBS or *ag85a/b* chimeric DNA by IM or EP at 3, 17, and 31 days. All mice were killed at 48 or 49 days and extracted mouse peripheral blood mononuclear cells (PBMC) messenger RNA (mRNA) for gene microarray assay. Finally, the expression levels of three DE genes were verified in human PBMC mRNA by RT‐qPCR. DE, differentially expressed; EP, electroporation; IM, intramuscular injection; MTB, *Mycobacterium tuberculosis*; PBS, phosphate‐buffered saline; RT‐qPCR, real‐time reverse transcription‐quantitive polymerase chain reaction.

The flowchart of the study design was shown in Figure 1.

### 2.1 Mouse TB models and DNA vaccine treatment

The preparation of the mouse TB model and the immunotherapy with DNA vaccine were conducted in our previous study.^31^ In brief，96 MTB H37Rv‐infected female BALB/c mice were randomly divided into the following groups: (1) phosphate‐buffered saline; (2) 10 μg *ag85a/b* DNA IM; (3) 50 μg *ag85a/b* DNA IM; (4) 100 μg *ag85a/b* DNA IM; (5) 200 μg *ag85a/b* DNA IM; (6) 10 μg *ag85a/b* DNA IM + EP; (7) 50 μg *ag85a/b* DNA IM + EP; (8) 100 μg *ag85a/b* DNA IM + EP; (9) 200 μg *ag85a/b* DNA IM + EP. On the 3rd day after MTB infection, the mice were immunized three times at 2‐week intervals, respectively. EP was performed at 36 V and 25 Hz, and six pulses of 10 ms in 3 mm depth of the thigh muscle of the hind leg of a mouse using a TERESA Gene Delivery Device (TERESA Health Technology Co., Ltd).

### 2.2 PBMC isolation and total RNA extraction

In this study, the whole blood from three mice in each group mentioned above was collected in an ethylenediaminetetraacetic acid dipotassium anticoagulant tube 3 weeks after the last immunization, and then isolated PBMCs with the Mouse PBMC Isolation Kit (Haoyang Biological Products Technology). Total RNA was extracted from PBMCs using TRIzol Reagent (Life Biotechnology) according to the manufacturer's instructions.

### 2.3 Gene chip expression analysis

Gene chip expression analysis was performed by Kangcheng Biotechnology Co., Ltd. using the Agilent array platform. First, the RNA quantity and quality were assessed using a NanoDrop ND‐1000 UV Spectrophotometer (Implen). RNA integrity of each sample was identified by standard denaturing agarose gel electrophoresis. Second, sample labeling and chip hybridization were performed according to the Agilent One‐Color Microarray‐Based Gene Expression Analysis protocol (Agilent). The samples were labeled by using the Agilent Quick Amp Labeling kit and hybridization experiments were performed by using the Agilent SureHyb. Specifically, the total RNA of each sample was linearly amplified and labeled with Cy3‐UTP; the labeled complementary RNAs (cRNAs) were purified by using the RNeasy Mini Kit (Qiagen), and the concentration and activity were detected with a NanoDrop ND‐1000 UV spectrophotometer. The purified Cy3‐UTP‐labeled cRNAs were hybridized with Agilent mouse 4 × 44 K gene expression profiling chip v2 (Agilent). The hybridization chip was washed, fixed, and scanned by using Agilent DNA Microarray Scanner (part number G2505C); Chip probe signal values were acquired by using Agilent Feature Extraction software (v11.0.1.1) to obtain raw data. Finally, the quantile normalization of raw data and subsequent data processing were performed by using GeneSpring GX v12.1 software (Agilent).

### 2.4 Differential gene screening and identification

DE genes after treatment with different doses of *ag85a/b* DNA vaccine immunized with IM or EP were screened by a hierarchical clustering heat map, scatter plot, and volcano plot analysis. The DE genes were compared between the TB model group and the normal control group and between each *ag85a/b* DNA treatment group and the TB model group. A hierarchical cluster plot shows differences in gene expression among different groups of samples. The scatter plot shows the DE gene correlation between the two groups of samples, where the *x*‐axis values and the *y*‐axis values are mean‐normalized (log2 scaling) processed results. The volcano plot shows the distribution of DE genes between the two groups of samples, and the *x*‐axis represents the log2 value (Fold Change), and the *y*‐axis represents the −log10 value (*p* value).

### 2.5 Gene Ontology (GO) analysis and Kyoto Encyclopedia of Genes and Genomes (KEGG) analysis

GO is an internationally standardized gene function classification system that comprehensively describes the properties of any biological gene and its products by using a dynamically updated controlled vocabulary and strictly defined concepts. In this study, significant DE genes between different groups of samples were subjected to GO terms for the three parts: molecular function (MF), biological process (BP), and cell component (CC) through the standard vocabulary provided by GO (http://www.geneontology.org) enrichment analysis to calculate the hypergeometric distribution relationship between DE genes and several specific branches in the GO classification to explore which gene function changes may be related to DE genes in different samples. The KEGG is a database that systematically analyzes gene functions, links genomic information and functional information, and the pathway database is the most widely used. Pathway analysis of DE genes was used to find the metabolic pathways enriched in DE genes, and to clarify which metabolic pathways may lead to the differential expression of genes between different groups of samples. GO analysis and pathway analysis utilized hypergeometric tests to calculate *p* values, which represent the importance of condition‐related pathways or GO terms enriched in DE genes. The lower the *p* value, the more significant the GO term or pathway (recommended cutoff value 0.05).

### 2.6 Validation of DE genes

About 3 mL of whole blood from 10 initially treated pulmonary TB patients and 15 healthy people controls (HC) was collected in a heparin sodium anticoagulant tube. The inclusion criteria of the TB group was sputum MTB‐positive, and/or interferon‐gamma release assay (IGRA)‐positive pulmonary TB patients without anti‐TB treatment or with anti‐TB treatment for less than 1 month. The inclusion criteria of HC were IGRA‐negative and without abnormal findings in lung computed tomography (CT) scan. All samples were respectively stimulated with MTB CFP‐10‐ESAT‐6 fusion protein for 24 h.^35^ Total RNA was extracted using Trizol reagent (Invitrogen), and cDNA was reverse‐transcribed from 1 μg of purified RNA using PrimeScript^TM^ RT Reagent Kit (Takara). The primer sequences for amplification of the DE genes were obtained from PrimerBank and synthesized by Sangon Biotech and shown in Table 1. RT‐qPCR amplification of the genes was performed using the RT‐qPCR kit with the SYBRGreen Ⅰ method (KAPA SYBR® FAST). The quantification of mRNA was examined using RT‐qPCR on Roche 480 (Roche) with the following program: predenatured at 95°C for 3 min; 40 cycles of denaturing at 95°C for 10 s, annealing at 60°C for the 20 s; extension at 72°C for 1 s. RT‐qPCR of each cDNA sample was repeated twice, and the final *C*_t_ value was the mean of the two times. The relative expression amount of Amy2a, Retn, and Sftpd was calculated by the $2^{‐△△C_{t}}$ method using the GAPDH gene as an internal reference.

**Table 1:** The primer sequences for amplification of the DE genes.

Gene name	Prime sequence forward 5′→3′	Prime sequence reverse 5′→3′
Amy2a	AATACACAACAAGGACGGACATC	TCCAAATCCCTTCGGAGCTAAA
Retn	CTGTTGGTGTCTAGCAAGACC	CCAATGCTGCTTATTGCCCTAAA
Sftpd	CGTCTTGTGGTCTGCGAGTTCTG	TGAGGGTCTAAGCCTTGACTTCTGG
GAPDH	TGCACCACCAACTGCTTA	GGATGCAGGGATGATGTTC

### 2.7 Validation by gene expression omnibus

A total of 11,824 studies were retrieved using the keyword “tuberculosis” in the GEO database (https://www.ncbi.nlm.nih.gov/geo/). Set the study type as expression profiling by array, species as *mus musculus* and *homo sapiens*, and sample type as the whole blood or PBMCs. The expression profile data of the GSE140943 data set^36^ composed of three uninfected mice and five 56 days infected mice and the expression profile data of six uninfected C57BL/6 mice and five 21 days infected C57BL/6 mice in the GSE89389 data set.^37^ In the GSE102459 data set,^33^ there were PBMC gene expression profile data of the M72/AS01 vaccine at 0 and 31 days of treatment. The DE genes were filtered using the GEO2R online analysis tool (set |log2FC| >1 and *p* < .05 as thresholds).^38^ We compared the DE genes in the three data sets with our research results in the TB model versus the normal group, 100 μg DNA IM versus TB model group, and 50 μg DNA EP versus TB model group. The GSE89403 data set using peripheral blood samples identified biomarkers for clinically relevant responses to TB treatment. Selected the DE genes both in the TB model versus the normal group and in the GSE89403 data set. The correlation between the six DE genes' Fold Change values at 4 and 24 weeks and anti‐TB treatment response was analyzed by multivariate logistic regression analysis with SPSS version 26. *p* Value < 0.05 was considered to have a significant difference.

### 2.8 Data analysis

The mouse gene expression profile was analyzed by assessing Fold Change, and the threshold for screening upregulated or downregulated DE genes were Fold Change ≥ 2, *p* < 0.05. Hierarchical clustering analysis by using R language. GO analysis and Pathway analysis were performed by using standard enrichment computational methods. The qPCR data were analyzed by GraphPad Prism 9. Student's *t* test was used for comparisons between the two groups. A *p*‐value less than 0.05 indicated statistical significance.

## 3 RESULT

### 3.1 Effect of *ag85a/b* DNA vaccine on the differential expression induced by MTB infection

#### 3.1.1 DE genes

**Figure 2:**
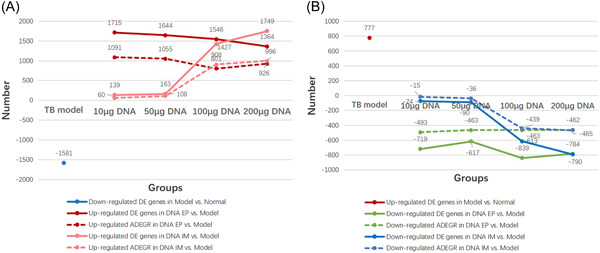
The total number of differentially expressed (DE) genes and abnormal differentially expressed gene recovered (ADEGR) between the tuberculosis (TB) model group and the normal group or each *ag85a/b* DNA vaccine group and TB model group. The *x*‐axis represents the group, the *y*‐axis represents the number of DE genes, >0 represents the upregulated DE gene, and <0 represents the downregulated DE gene. ADEGR refers to those DE genes significantly upregulated or downregulated in the TB model group were recovered to varying degrees in each *ag85a/b* DNA vaccine group. (A) The number of those DE genes significantly downregulated in the TB model group and recovered in each *ag85a/b* DNA vaccine group. (B) The number of those DE genes significantly upregulated in the TB model group and recovered in each *ag85a/b* DNA vaccine group. EP, electroporation; IM, intramuscular injection.

After MTB infection, the gene expression in mice was significantly abnormal, there were 777 DE genes upregulated and 1581 DE genes downregulated. The gene expression variation between the TB model group and the normal control group or between each *ag85a/b* DNA treatment group and TB model group was analyzed and visualized by the scatter plot, volcano plot, and cluster plot (shown in Supporting Information: Figure 1). Many DE genes significantly upregulated or downregulated in the TB model group were recovered to varying degrees in each *ag85a/b* DNA vaccine treatment group. The total number of DE genes and abnormal DE genes recovered (ADEGR) in each group were shown in Figure 2. The results showed that: (1) The gene expression was significantly abnormal in mice infected with MTB. Most DE genes with significantly upregulated or downregulated expression were recovered after treatment with 100, 200 μg DNA IM, or 10–200 μg DNA EP. (2) The number of DE genes upregulated or downregulated in the *ag85a/b* DNA IM group was positively correlated with the DNA injection dose. When the IM doses increased from 50 to 100 μg, both the number of upregulated DE genes and the number of downregulated DE genes increased greatly, while when the injection dose increased from 100 to 200 μg, the number of upregulated and downregulated DE genes increased slowdown. (3) The number of upregulated DE genes in each dose of the DNA EP group was negatively correlated with the immune dose, but the downward trend was not obvious. There was no obvious correlation between the number of downregulated DE genes and the dose of DNA EP. (4) The number of DE genes in the 10 μg DNA EP group was equivalent to that in the 200 μg DNA IM group.

The changes in the top 20 DE genes significantly upregulated and downregulated after MTB infection or after treatment with *ag85a/b* DNA vaccine IM and EP are shown in Tables 2 and 3, respectively. These genes may be involved in the occurrence and development of TB, and reveal the new potential preventative and immunotherapeutic targets of the *ag85a/b* DNA vaccine for TB. The results show that: (1) The significantly upregulated or downregulated top 20 DE genes in the TB model group had no significant change in the 10 μg DNA IM group and 50 μg DNA IM group (*p* ≥ .05) and were all recovered significantly in 100 μg DNA IM group and 200 μg DNA IM group (*p* < .05). (2) The abnormally upregulated or downregulated DE genes in the TB model group were all recovered in the 4 *ag85a/b* DNA EP group. Furthermore, the Fold Change values of upregulated or downregulated DE genes in the TB model group were reversed in 4 DNA EP groups. (3) Most of the upregulated DE genes in the TB model group were related to the digestion and absorption of nutrients or neuroendocrine (Table 2), for example, Iapp, Scg2, Amy2a5, Try5, Chga, Cpa1, Gcg, and so forth. Most of the downregulated DE genes in the TB model group were related to cellular structural proteins and cellular functional proteins (Table 3), in which the structure and function proteins of alveolar epithelial cells account for a large proportion, for example, Sftpc, Sftpb, Sftpd, Sftpa1, Wfdc2, Sec1413, Postn, Cldn5, Aqp5, Emp2, and Foxf1, and so forth.

**Table 2:** The top 20 significantly upregulated DE genes between the TB model and the normal group and their changes in various *ag85a/b* DNA versus TB model groups.

Genbank accession	Gene symbol	Fold Change values of the DE genes									Annotation
		TB model group versus Normal group	DNA IM group versus TB model group				DNA EP group versus TB model group				
			10 μg	50 μg	100 μg	200 μg	10 μg	50 μg	100 μg	200 μg	
NM_010491	Iapp	832↑	NO	NO	620↓	981↓	665↓	640↓	760↓	843↓	Islet amyloid polypeptide, has direct toxicity to islet B cells, mediates local inflammatory reaction, leading to pancreatic islet dysfunction,^39^, ^40^, ^41^ an important pathological factor causing type 2 diabetes mellitus.
NM_009129	Scg2	428↑	NO	NO	391↓	363↓	382↓	345↓	396↓	399↓	Secretogranin II, has anti‐inflammatory properties and participates in inflammatory reaction, involves in the pathogenesis of diabetes.^42^, ^43^
NM_001042711	Amy2a5	365↑	NO	NO	239↓	356↓	285↓	272↓	457↓	400↓	Amylase alpha 2A, a member of the amylase family, involves in starch digestion and glycogen metabolism.^44^
NM_001003405	Try5	337↑	NO	NO	314↓	295↓	330↓	287↓	313↓	239↓	Trypsin 5, involves in the hydrolysis of proteins and peptide chains.
NM_007693	Chga	326↑	NO	NO	433↓	440↓	317↓	342↓	440↓	408↓	Chromogranin A, has anti‐inflammatory properties and participates in inflammatory reaction, involves in the pathogenesis of diabetes.^43^, ^45^
NM_025350	Cpa1	309↑	NO	NO	312↓	297↓	274↓	305↓	307↓	307↓	Carboxypeptidase A1, hydrolyzes C‐terminal aromatic or neutral aliphatic amino acids residues of in proteins and polypeptide substrates.
NM_008100	Gcg	260↑	NO	NO	326↓	324↓	323↓	212↓	341↓	275↓	Glucagon, a kind of pleiotropic hormone with metabolic effects secreted by islet α cells, which can promote glycogen decomposition and gluconeogenesis to increase blood sugar, and also can promote fat decomposition and lipid oxidation.^46^
NM_001126318	Gm13011	218↑	NO	NO	218↓	234↓	228↓	178↓	219↓	231↓	Elastase 3A, a member of the elastase family, involves in the hydrolysis of various proteins.
NM_198627	Vstm2l	198↑	NO	NO	199↓	136↓	180↓	182↓	197↓	197↓	V‐set and transmembrane domain containing two like, a novel modulator of neuroprotective activity. Overexpression of VSTM2L in a variety of cancer samples regulates IL‐4 signaling pathway, mainly enrichs in cell signal transduction, immune response, inflammatory response, calcium binding, and so forth.^47^
NM_008386	Ins1	177↑	NO	NO	181↓	259↓	207↓	184↓	253↓	182↓	Insulin 1, a protein hormone secreted by the islets β cells, which can reduce blood sugar and promote the synthesis of glycogen, fat and protein.
NM_153518	Ccdc65	163↑	NO	NO	87↓	137↓	168↓	154↓	115↓	159↓	Coiled‐coil domain containing 65, a member of the coiled‐coil domain‐containing protein family, encodes many proteins like motor and skeletal proteins and involved in protein refolding and molecular recognition systems.^48^
NM_011646	Try4	140↑	NO	NO	250↓	253↓	252↓	240↓	205↓	248↓	Trypsin 4, involved in the hydrolysis of proteins and peptide chains.
NM_009430	Prss2	135↑	NO	NO	179↓	206↓	151↓	198↓	205↓	137↓	Serine protease 2, a member of the trypsin family, plays a role in food digestion.
NM_029706	Cpb1	121↑	NO	NO	125↓	103↓	124↓	124↓	124↓	124↓	Carboxypeptidase B1, cleaves the C‐terminal of lysine or arginine, used as a serological marker of acute pancreatitis.
NM_026925	Pnlip	112↑	NO	NO	60↓	71↓	219↓	111↓	187↓	145↓	Pancreatic lipase, a member of the lipase family, secreted by the pancreas, hydrolyzes dietary triglycerides in the small intestine.^49^
NM_007919	Cela2a	99↑	NO	NO	103↓	103↓	103↓	84↓	102↓	81↓	Chymotrypsin‐like elastase 2A, a member of the elastase family, circulates in plasma, reduces platelet hyperactivation, triggers insulin secretion and degradation, and increases insulin sensitivity.^50^
NM_007694	Chgb	91↑	NO	NO	91↓	89↓	66↓	49↓	54↓	64↓	Chromogranin B, a member of the chromogranins/secretogranins protein family, has both extracellular and intracellular roles in the neuroendocrine system.
NM_009049	Resp18	81↑	NO	NO	80↓	81↓	76↓	63↓	43↓	59↓	Regulated endocrine specific protein 18, a novel secreted peptide, specifically expressed in mammalian central nervous system and endocrine system.^51^
NM_172816	Slc30a8	78↑	NO	NO	58↓	44↓	72↓	80↓	69↓	60↓	Solute carrier family 30 member 8, a zinc transporter, transports Zinc from cytoplasm to insulin secretory granules in the pancreatic beta‐cells.^52^
NM_007446	Amy1	75↑	NO	NO	53↓	57↓	62↓	65↓	54↓	58↓	Amylase 1, hydrolyzes 1,4‐alpha glycosidic bonds within starch, and plays a key role in starch digestion.

Abbreviations: DE, differentially expressed; EP, electroporation; IM, intramuscular injection; TB, tuberculosis; ↑, upregulated expression; ↓, downregulated expression.

**Table 3:** The top 20 significantly downregulated DE genes in the TB model versus normal group and their changes in various *ag85a/b* DNA versus TB model group.

Genbank accession	Gene symbol	Fold Change values of the DE genes									Annotation
		TB model group versus Normal group	DNA IM group versus TB model group				DNA EP group versus TB model group				
			10 μg	50 μg	100 μg	200 μg	10 μg	50 μg	100 μg	200 μg	
NM_011359	Sftpc	167↓	NO	NO	559↑	395↑	255↑	289↑	565↑	280↑	Surfactant protein C (SFTPC), a hydrophobic protein secreted by alveolar epithelial cells, maintains lung tissue stability by reducing the surface tension of the fluid covering the lungs.^53^
NM_001282071	Sftpb	68↓	NO	NO	348↑	253↑	100↑	129↑	207↑	99↑	SFTPB, an amphoteric SFTP secreted by alveolar epithelial cells, increases the diffusion rate and stability of the surfactant layer in vitro.^54^
NM_009160	Sftpd	48↓	NO	NO	141↑	60↑	59↑	78↑	146↑	98↑	SFTPD, a member of the collectin family, is soluble innate immune molecule which maintain lung homeostasis through their dual roles as anti‐infectious and immunomodulatory agents.^55^, ^56^
NM_009349	Inmt	43↓	NO	NO	164↑	154↑	39↑	36↑	223↑	116↑	Indolethylamine N‐methyltransferase, is a methyltransferase that regulates the N‐methylation of tryptamine family proteins, participates in the development and activity of the nervous system.^57^
NM_023134	Sftpa1	32↓	NO	NO	127↑	102↑	64↑	50↑	127↑	75↑	SFTPA1, a member of type C lectin subfamily, plays an important role in surfactant homeostasis and defense against respiratory pathogens, and mediates adhesion and phagocytosis of MTB by alveolar macrophages.^58^
NM_010329	Pdpn	32↓	NO	NO	57↑	27↑	38↑	66↑	55↑	41↑	Podoplanin, a small transmembrane mucin‐like glycoprotein that plays an important role in immune response. In different inflammation‐related diseases, the expression of Pdpn in immune cells participates in the regulation of inflammation.^59^
NM_026323	Wfdc2	32↓	NO	NO	117↑	66↑	53↑	73↑	113↑	70↑	Whey acidic protein four‐disulfide core domain 2, a small secreted protein that functions as a protease inhibitor, plays critical roles in multiple aspects of lung function, such as promoting mucociliary clearance, conferring anti‐inflammatory activity, and reducing surface tension.^60^
NM_001029937	Sec1413	28↓	NO	NO	116↑	185↑	83↑	86↑	237↑	139↑	SEC14‐like 3, a 45 kDa secretory protein specifically expressed in airway epithelial cells, has a close relationship with airway inflammation, and decreases significantly with the aggravation of airway inflammation, which may be a new marker of airway inflammation.^61^, ^62^
NM_001198766	Postn	27↓	NO	NO	87↑	52↑	46↑	79↑	101↑	71↑	Periostin, a 90‐kDa secreted extracellular matrix protein, binds to many extracellular matrix proteins through its different domains, and can bind to diverse integrins to activate the TGF‐β, PI3K/Akt, Wnt, RhoA/ROCK, NF‐κB, MAPK, and JAK pathways.^63^
NM_013805	Cldn5	23↓	NO	NO	52↑	28↑	19↑	36↑	66↑	37↑	Claudin‐5, a member of the Claudin family, is an integral membrane protein and a component of tight junctions, which act as physical barriers preventing free passage of solutes and water through the paracellular space between epithelial or endothelial cell sheets.
NM_007817	Cyp2f2	22↓	NO	NO	166↑	209↑	54↑	48↑	314↑	138↑	Cytochrome P450, family 2, subfamily f, polypeptide 2, is a monooxygenase that catalyzes many reactions in drug metabolism and metabolizes a variety of pulmonary toxicants.^64^
NM_009701	Aqp5	22↓	NO	NO	31↑	18↑	22↑	25↑	35↑	31↑	Aquaporin 5, a small water channel membrane proteins associated with major intrinsic proteins (p38, MIP etc.), mediates cell migration and proliferation, and participates in inflammatory reaction.^65^
NM_007929	Emp2	21↓	NO	NO	64↑	47↑	41↑	45↑	77↑	53↑	Epithelial membrane protein 2, a member of the tetraspan superfamily of membrane protein, has a variety of functions, including endocytosis, cell signaling, proliferation, migration, and adhesion.^66^
NM_020509	Retnla	21↓	NO	NO	24↑	NO	27↑	95↑	12↑	18↑	Resistin‐like alpha, a member of the resistin family, is a secreted cysteine‐rich protein with insulin resistance and also an anti‐inflammatory marker of macrophages.^67^, ^68^
NM_011315	Saa3	20↓	NO	NO	NO	NO	NO	56↑	NO	NO	Serum amyloid A3, a pseudogene in humans, is a major component of acute‐phase inflammation, serves as an endogenous peptide ligand for TLR4, and binds MD‐2 to activate p38 and NF‐κB pathways in a MyD88‐dependent manner.^69^
NM_010426	Foxf1	17↓	NO	NO	86↑	68↑	26↑	39↑	124↑	59↑	Forkhead box F1, a mesenchymal transcriptional factor essential for lung development, promotes normal lung homeostasis and repair.^70^
NM_008485	Lamc2	17↓	NO	NO	22↑	14↑	23↑	27↑	24↑	19↑	Laminin subunit gamma 2, laminin is an extracellular matrix glycoprotein and the important adhesive component of the epithelial basement membrane. They are involved in various biological processes such as cell adhesion, differentiation, migration, signaling, neurite outgrowth and metastasis.^71^
NM_008597	Mgp	17↓	NO	NO	32↑	21↑	26↑	48↑	40↑	27↑	Matrix gla protein, a vitamin K‐dependent inhibitor of calcification, may play an anti‐inflammatory role in monocytes and macrophages.^72^
NM_008344	Igfbp6	16↓	NO	NO	58↑	38↑	19↑	15↑	66↑	39↑	Insulin‐like growth factor binding protein 6, prolongs the half‐life of insulin‐like growth factor (IGF), inhibits or stimulates the growth‐promoting effect of IGF in cell culture, activates the MAPK signaling pathway, and induces cell migration ^73^
NM_010217	Ctgf	15↓	NO	NO	31↑	18↑	25↑	50↑	53↑	44↑	Connective tissue growth factor, a 38‐kDa protein with a tetramodular structure, is involved in the basic lung development process and promotes lung fibroblast proliferation, migration, and differentiation.^74^

Abbreviations: DE, differentially expressed; EP, electroporation; IM, intramuscular injection; MTB, *Mycobacterium tuberculosis*; TB, tuberculosis; ↑, upregulated expression; ↓, downregulated expression.

#### 3.1.2 GO analyses of DE genes

The significant DE genes between the TB model group and the normal group or each *ag85a/b* DNA vaccine group and TB model group were subjected to GO terms for the BP, CC, and MF by GO analyses, and the top 10 significant BP, CC, and MF items enriched in each group were compared (Supporting Information: Figure 2). The results showed that: (1) In the TB model group, the upregulated BP items were mainly enriched in the secretion and regulation of cells, proteins, insulin and hormones, and so forth; the downregulated BP items were mainly enriched in locomotion, developmental process, and cell migration, and so forth. The upregulated CC items were mainly enriched in the host cell cytoplasm, host intracellular part, neuronal cell body, and secretory granule, and so forth; the downregulated CC items were mainly enriched in extracellular matrix part, extracellular region, plasma membrane, and the vesicle, and so forth. The upregulated MF items were mainly enriched in ligand activation, G‐protein coupled receptor binding, calcium ion binding, peptidase activity, and hormone activity, and so forth. the downregulated MF items were mainly enriched in binding, protein binding, ion binding, and receptor binding, and so forth. (2) Among the top 10 significantly upregulated or downregulated GO items in the TB model group, the 10 and 50 μg DNA IM groups had little effect on them, most upregulated GO items were significantly downregulated, and most downregulated GO items were significantly upregulated in 100, 200 μg DNA IM groups, and four DNA EP groups.

#### 3.1.3 Enrichment analyses of the signal pathway

We performed the analysis on the pathway enrichment of DE genes by using the KEGG database to screen out the enriched metabolic pathways or signal transduction pathways. We selected the top 20 pathways ranked by enrichment score in the TB model group, and then counted the enrichment scores of these pathways in each *ag85a/b* DNA vaccine group to analyze the effects of *ag85a/b* DNA vaccine on metabolic pathways or signal transduction pathways and the repair effects of the damage caused by MTB infection (Tables 4 and 5). The results showed that: (1) Most of the pathways with upregulated enrichment scores in the TB model group were related to nutrient digestion and absorption or endocrine and neuroendocrine, for example, insulin secretion, digestion and absorption of protein and Fat, neuroactive ligand‐receptor interaction, and so forth. Most of the pathways with downregulated enrichment scores in the TB model group were related to immune responses or enzyme metabolism, for example, cytokine–cytokine receptor interaction, chemokine signaling pathway, Focal adhesion, ECM‐receptor interaction, and metabolism of xenobiotics by cytochrome P450, and so forth. (2) The enrichment score of the top 20 pathways in the TB model group had no significant change in the 10 and 50 μg DNA IM groups. However, most of them were reversed in the other 6 groups with the *ag85a/b* DNA vaccine.

**Table 4:** The top 20 significantly upregulated pathways in TB model group and their changes in various *ag85a/b* DNA vaccine groups.

Pathway ID	Definition	Enrichment score of the pathway								
		TB model group versus Normal group	DNA IM group versus TB model group				DNA EP group versus TB model group			
			10 μg	50 μg	100 μg	200 μg	10 μg	50 μg	100 μg	200 μg
mmu04972	Pancreatic secretion	11.335708↑	NO	NO	13.494424↓	11.410462↓	15.449871↓	13.102152↓	10.378839↓	11.678653↓
mmu04974	Protein digestion and absorption	6.798788↑	NO	NO	9.228974↓	7.719846↓	7.406446↓	8.944734↓	7.665778↓	7.913878↓
mmu04975	Fat digestion and absorption	4.937938↑	NO	NO	4.715005↓	4.016645↓	4.29808↓	4.584038↓	3.991484↓	4.106856↓
mmu04911	Insulin secretion	3.887882↑	NO	NO	4.053136↓	4.653304↓	4.676213↓	NO	4.615988↓	4.039977↓
mmu04080	Neuroactive ligand‐receptor interaction	3.434282↑	NO	NO	3.895453↓	6.009911↓	3.649268↓	NO	3.434282↓	4.641685↓
mmu04950	Maturity onset diabetes of the young	3.101886↑	NO	NO	5.934764↓	5.205878↓	4.619483↓	NO	4.097165↓	6.481842↓
mmu00561	Glycerolipid metabolism	2.924039↑	NO	NO	2.069674↓	1.636779↓	1.987283↓	NO	1.621508↓	1.691735↓
mmu04713	Circadian entrainment	2.7979↑	NO	NO	2.297331↓	NO	2.188517↓	NO	2.228↓	1.802273↓
mmu05030	Cocaine addiction	2.644894↑	NO	NO	2.414299↓	1.960102↓	1.619297↓	2.710407↓	1.943952↓	1.38322↓
mmu05164	Influenza A	2.532544↑	NO	NO	1.508885↓	NO	1.40679↓	NO	NO	NO
mmu04917	Prolactin signaling pathway	2.354739↑	NO	NO	2.299486↓	2.376645↓	2.202203↓	NO	NO	1.322084↓
mmu04010	MAPK signaling pathway	2.327035↑	NO	NO	NO	NO	1.559062↓	NO	NO	NO
mmu04970	Salivary secretion	2.2593↑	NO	1.541781↓	2.215196↓	1.712174↓	1.5306↓	NO	NO	NO
mmu04710	Circadian rhythm	1.987694↑	NO	NO	NO	NO	NO	NO	NO	1.309626↓
mmu04912	GnRH signaling pathway	1.949105↑	NO	NO	NO	NO	1.847607↓	NO	1.443851↓	NO
mmu05031	Amphetamine addiction	1.941061↑	NO	NO	NO	1.403914	NO	NO	1.941061↓	1.456146↓
mmu04728	Dopaminergic synapse	1.883599↑	NO	NO	2.070013↓	1.477067↓	2.184166↓	1.486645↓	1.883599↓	NO
mmu05032	Morphine addiction	1.825693↑	NO	NO	2.423023↓	1.845834↓	2.66319↓	NO	NO	NO
mmu04614	Renin‐angiotensin system	1.804049↑	NO	NO	4.057283↓	3.460628↓	2.788612↓	2.121122↓	5.399902↓	2.656079↓
mmu04961	Endocrine and other factor‐regulated calcium reabsorption	1.772715↑	NO	NO	3.847204↓	3.181395↓	3.448604↓	2.897346↓	4.808097↓	3.266867↓

Abbreviations: DE, differentially expressed; EP, electroporation; IM, intramuscular injection; TB, tuberculosis; ↑, upregulated score; ↓, downregulated score.

**Table 5:** The top 20 significantly downregulated pathway in TB model group and their changes in various *ag85a/b* DNA vaccine groups.

Pathway ID	Definition	Enrichment score of the pathway								
		TB model group versus Normal group	DNA IM group versus TB model group				DNA EP group versus TB model group			
			10 μg	50 μg	100 μg	200 μg	10 μg	50 μg	100 μg	200 μg
mmu04060	Cytokine‐cytokine receptor interaction	6.389233↓	NO	1.617686↑	1.422668↑	5.262387↑	4.452331↑	2.437235↑	5.027105↑	3.917855↑
mmu05133	Pertussis	4.154819↓	NO	NO	2.424137↑	3.131711↑	2.76652↑	2.846166↑	1.682164↑	2.482508↑
mmu04974	Protein digestion and absorption	3.682461↓	NO	NO	6.066787↑	4.744358↑	2.866031↑	5.672109↑	7.386822↑	4.903948↑
mmu04360	Axon guidance	2.756632↓	NO	NO	2.442363↑	4.352212↑	3.643141↑	3.325582↑	6.778728↑	3.843059↑
mmu04933	AGE‐RAGE signaling pathway in diabetic complications	2.609451↓	NO	NO	4.077841↑	5.564438↑	3.175328↑	4.263249↑	5.156242↑	4.17245↑
mmu04512	ECM‐receptor interaction	2.574761↓	NO	NO	8.116145↑	5.285638↑	3.243851↑	7.645445↑	11.261432↑	6.069831↑
mmu04610	Complement and coagulation cascades	2.391915↓	1.769156↑	NO	5.643564↑	4.968025↑	4.035406↑	4.144754↑	5.654917↑	3.91844↑
mmu00590	Arachidonic acid metabolism	2.263825↓	NO	2.628508↑	2.655805↑	NO	NO	NO	NO	NO
mmu05150	*Staphylococcus aureus* infection	2.220857↓	NO	NO	2.522364↑	2.986781↑	2.540976↑	2.605515↑	3.381485↑	2.573856↑
mmu00980	Metabolism of xenobiotics by cytochrome P450	2.042694↓	2.112486↑	2.112486↑	2.886266↑	4.337605↑	1.437203↑	1.8995↑	3.070669↑	1.482553↑
mmu00982	Drug metabolism ‐ cytochrome P450	1.951735↓	2.076184↑	2.076184↑	3.331959↑	6.833289↑	1.754782↑	2.742079↑	5.387121↑	2.315482↑
mmu05146	Amoebiasis	1.914775↓	NO	NO	2.30181↑	3.510302↑	2.393835↑	2.893891↑	4.645635↑	2.799971↑
mmu04510	Focal adhesion	1.881851↓	NO	NO	6.102566↑	7.401803↑	4.954266↑	8.554973↑	9.255016↑	8.402425↑
mmu04062	Chemokine signaling pathway	1.74527↓	NO	NO	NO	1.560615↑	1.451812↑	1.793169↑	NO	NO
mmu00910	Nitrogen metabolism	1.662142↓	NO	NO	1.815201↑	NO	NO	NO	1.670625↑	1.841053↑
mmu04350	TGF‐beta signaling pathway	1.656492↓	NO	NO	1.568223↑	2.109613↑	NO	2.298151↑	2.973736↑	2.02199↑
mmu00730	Thiamine metabolism	1.629587↓	NO	NO	NO	NO	NO	NO	NO	NO
mmu00480	Glutathione metabolism	1.550779↓	NO	2.292867↑	2.299118↑	2.709633↑	NO	NO	3.092353↑	1.838204↑
mmu04151	PI3K‐Akt signaling pathway	1.526425↓	NO	NO	4.191528↑	3.487234↑	2.67112↑	4.990313↑	6.414413↑	5.464275↑
mmu05144	Malaria	1.411386↓	1.42469↑	1.42469↑	NO	1.632998↑	1.708676↑	1.313239↑	NO	NO

Abbreviations: DE, differentially expressed; EP, electroporation; IM, intramuscular injection; TB, tuberculosis; ↑, upregulated score; ↓, downregulated score.

### 3.2 Action target and mechanism of *ag85a/b* DNA vaccine

Based on the results from our team's previous pharmacodynamic study^31^ and the preceding part above, the 100 μg *ag85a/b* DNA IM and 50 μg *ag85a/b* DNA EP were optimal effective therapeutic doses. Therefore, we analyzed the DE genes in the 100 μg DNA IM group and 50 μg DNA EP group to explore the action targets and mechanism of the DNA vaccine.

#### 3.2.1 DE genes

**Figure 3:**
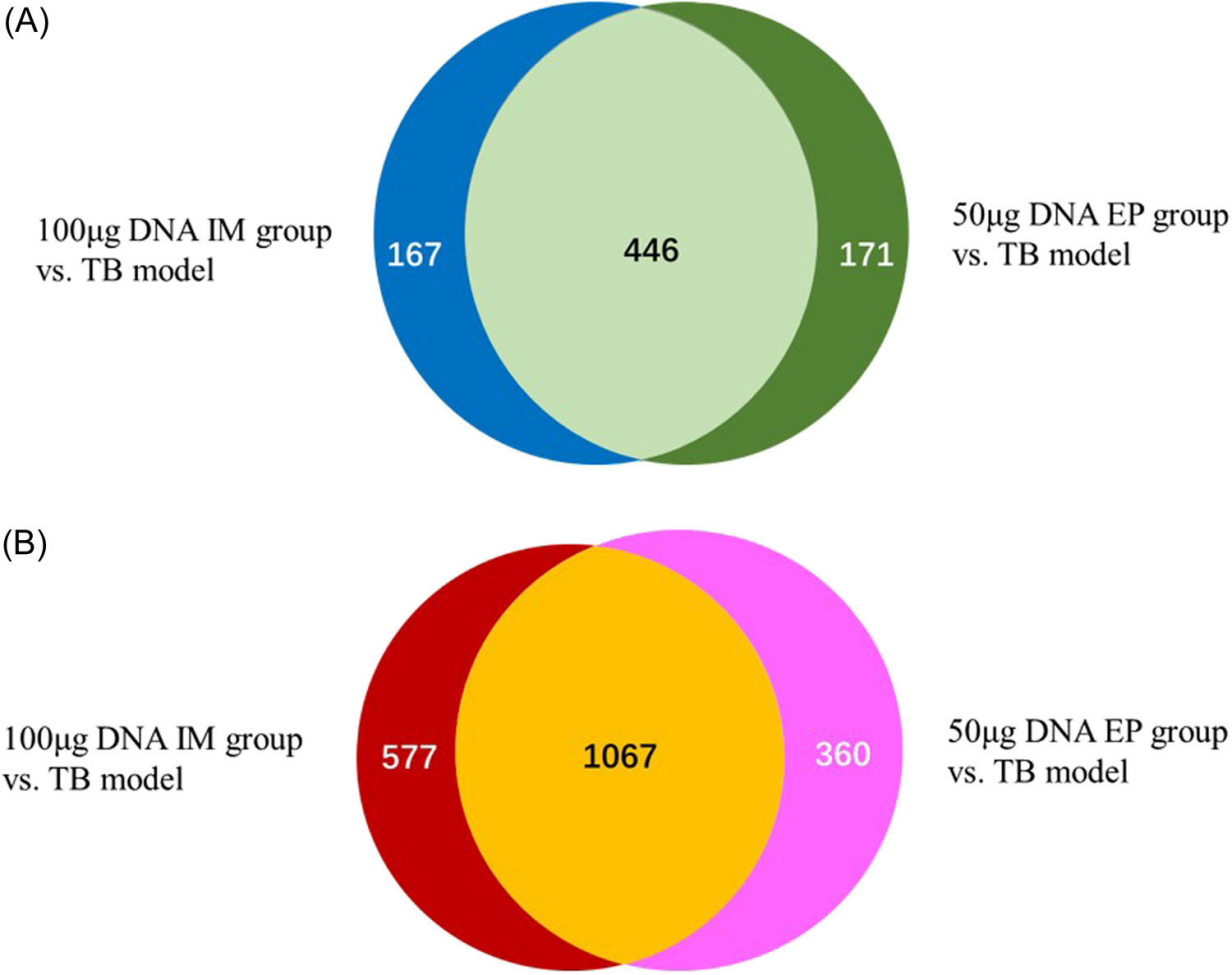
The analysis of differentially expressed (DE) genes between 100 μg DNA intramuscular injection (IM) group and 50 μg DNA electroporation (EP) group. (A) The circle on the left represents the number of downregulated DE genes in the 100 μg DNA IM group versus tuberculosis (TB) model group and the circle on the right represents the number of downregulated DE genes in the 50 μg DNA EP group versus TB model group. The intersection part represents the number of the same DE genes between the two groups. (B) The circle on the left represents the number of upregulated DE genes in the 100 μg IM versus TB model group and the circle on the right represents the number of upregulated DE genes in the 50 μg DNA EP versus TB model group. The intersection part represents the number of the same DE genes between the two groups.

The analysis of DE genes between the 100 μg DNA IM group and 50 μg DNA EP group was shown in Figure 3. The concordance rates of downregulated and upregulated DE genes between the two groups were 56.9% and 53.2%, respectively. First, we screened out the top 20 upregulated DE genes and downregulated DE genes in the 100 μg DNA IM group, and then counted the differential expression levels of these DE genes in the TB model group and 50 μg DNA EP group (Supporting Information: Tables 1, 2). Second, we screened out the top 20 upregulated DE genes and downregulated DE genes in the 50 μg EP group, and then collected the differential expression levels of these DE genes in the TB model group and 100 μg DNA IM group (Supporting Information: Tables 3, 4). Finally, we compared the consistency of DE genes in four tables to analyze the commonality of the action targets of the two vaccination methods (IM and EP) in the mouse TB model. The results showed that: (1) Of the top 20 downregulated and the top 20 upregulated DE genes in the 100 μg DNA IM group and 50 μg DNA EP group, 18 (81.8%) downregulated DE genes and 13 (48.1%) upregulated DE genes between the two groups were the same. (2) Most of the DE genes significantly downregulated in the 100 μg DNA IM group and 50 μg DNA EP group were related to the digestion and absorption of nutrients or neuroendocrine, for example, dysfunction of the pancreatic island (Iapp, Scg2, Chga, Chgb), metabolism of carbohydrate and glycogen (Amy2a5, Gcg, Ins1), protein hydrolysis (Try5, Cpa1, Gm13011, Try4, Cpb1), lipid metabolism (Ins1, Pnlip), neuroendocrine (Vstm2l, Resp18), and so forth. Most of the significantly upregulated DE genes were related to the cellular structural proteins and cellular functional proteins, in which alveolar cell structure and function proteins account for a large proportion, for example, Sftpc, Sftpc, Sftpb, Sftpd, Sftpa1, Wfdc2, Sec1413, Postn, Cldn5, Aqp5, Emp2, and Foxf1, and so forth. (3) The expressions of the top 20 DE genes in 100 μg DNA IM group and 50 μg DNA EP group, whether upregulated or downregulated, were opposite to those in the TB model group.

#### 3.2.2 GO analyses of DE genes

After comparing and analyzing the GO results for the BP, CC, and MF in the 100 μg DNA IM group and 50 μg DNA EP group (Supporting Information: Figure 2), we found that: (1) 20 of the 30 GO items in downregulated DE genes and 22 of the 30 GO items in upregulated DE genes in the two groups are consistent. (2) In the two DNA groups, the downregulated BP items were mainly enriched in the secretion and regulation of cells, proteins, insulin, hormones, and so forth, in addition, DNA EP significantly decreased the secretion and regulation of neurotransmitters at the same time; the upregulated BP items were mainly enriched in the developmental process, and so forth. The downregulated CC items were mainly enriched in the neuronal cell body, secretory granule, synapse part, extracellular region, and so forth; the upregulated CC items were mainly enriched in the extracellular region, extracellular matrix part, vesicle, so forth. The downregulated MF items were mainly enriched in peptidase activity, calcium ion binding, hormone activity, triglyceride lipase activity, so forth; the upregulated MF items were mainly enriched in binding, protein binding, receptor binding, growth factor binding, ion binding, so forth.

#### 3.2.3 Enrichment analyses of the signal pathway

We also performed the analysis on pathway enrichment of DE genes in 100 μg *ag85a/b* DNA IM group and 50 μg *ag85a/b* DNA EP group. The pathway analysis results revealed the action pathway of *ag85a/b* DNA vaccine (Supporting Information: Tables [Link], [Link]): (1) There were respectively 15 identical pathways in both upregulated and downregulated DE genes of the 100 μg DNA IM group and 50 μg DNA EP group. (2) The downregulated pathways in the 100 μg DNA IM group and 50 μg DNA EP group were mainly related to nutrient digestion and absorption, hormone and neurotransmitter secretion, endocrine and neuroendocrine, for example, pancreatic secretion, protein digestion and absorption, maturity‐onset diabetes of the young, insulin secretion, fat digestion and absorption, renin‐angiotensin system, neuroactive ligand‐receptor interaction, and so forth; the upregulated pathways were mainly related to immune defense, substance metabolism and tumor, for example, ECM‐receptor interaction, focal adhesion, protein digestion and absorption, PI3K‐Akt signaling pathway, proteoglycans in cancer, and so forth.

**Figure 4:**
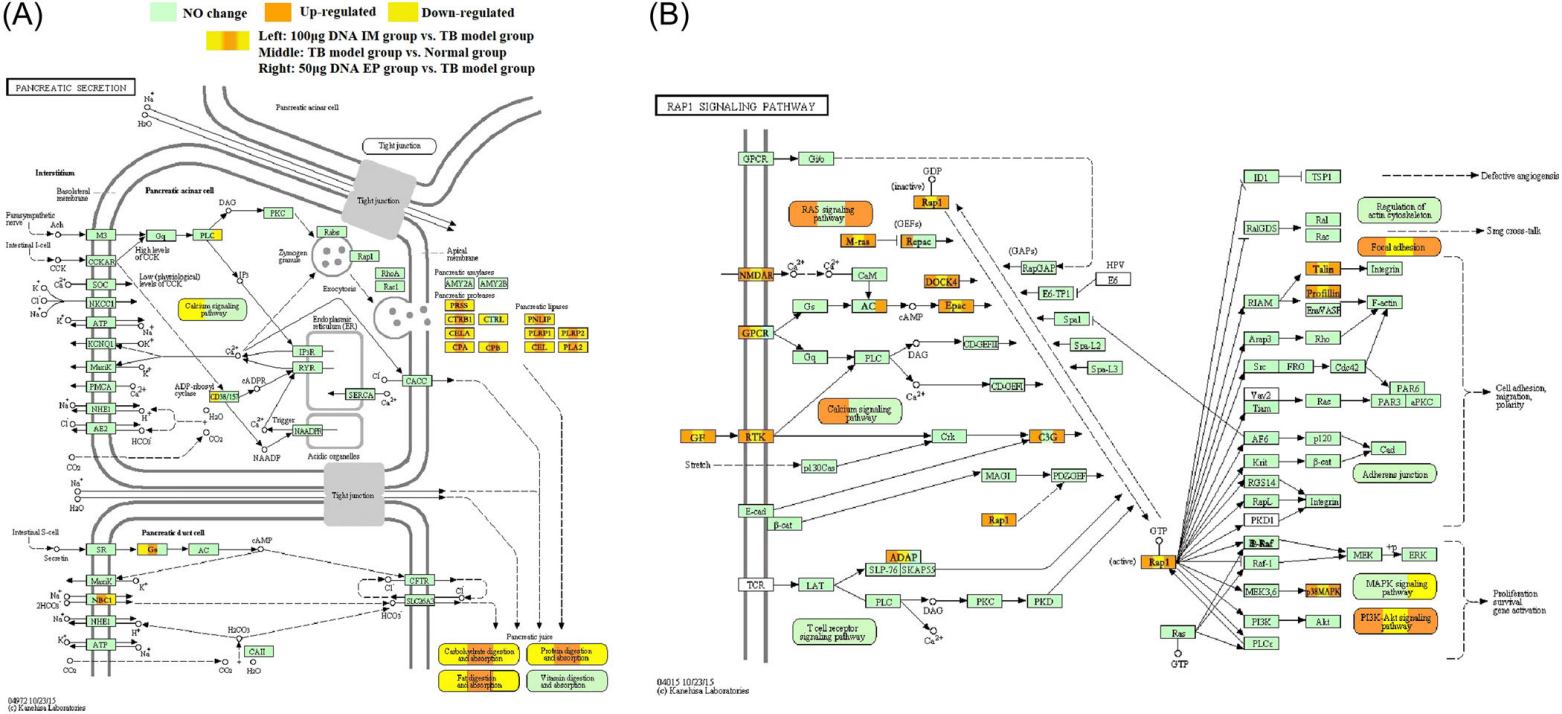
The expression of pancreatic secretion pathway and Rap1 signaling pathway before and after the treatment with 100 μg *ag85a/b* DNA IM or 50 μg DNA EP. (A) Pancreatic secretion pathway. (B) Rap1 signaling pathway. EP, electroporation; IM, intramuscular injection.

After further analysis of the specific pathways, we found that the pancreatic secretion pathway downregulated and Rap1 signal pathway upregulated had particularly significant changes during the immunotherapy of the *ag85a/b* DNA vaccine on the mouse TB model. The former was closely related to the digestion and absorption of nutrients, and the latter was related to immunity. Therefore, we focused on the regulatory changes of these two pathways before and after *ag85a/b* DNA vaccine treatment to explore the therapeutic effect of *ag85a/b* DNA vaccine on damage recovery caused by MTB infection and the mechanism of immunotherapy (shown in Supporting Information: Figure 3 and Figure 4). We found that the expression levels of 15 DE genes in the pancreatic secretion pathway were significantly changed before and after *ag85a/b* DNA treatment, in which the expression levels of 12 DE genes, NBC1, GS, PRSS, CTRB1, CELA, CPA, CPB, PNLIP, PLRP1, CEL, PLRP2, and PLA2, increased significantly after MTB infection (shown in Supporting Information: Figure 3A), but decreased significantly after *ag85a/b* DNA treatment (shown in Supporting Information: Figure 3B,C and Figure 4A). The expression levels of 15 DE genes in the Rap1 signal pathway were significantly changed before and after *ag85a/b* DNA treatment, in which the expression levels of 12 DE genes, GF, NMDAR, RTK, GPCR, ADAP, M‐ras, Rap1, DOCK4, Epac, Talin, Profillin, p38MAPK, decreased significantly after MTB infection (shown in Supporting Information: Figure 3D), but increased significantly after *ag85a/b* DNA treatment (shown in Supporting Information: Figure 3E,F and Figure 4B).

### 3.3 RT‐qPCR and GEO validation

**Figure 5:**
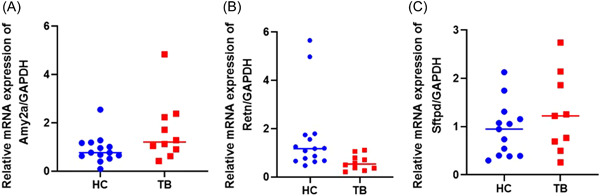
Transcript levels of Retn, Amy2a, and Sftpd genes in the human peripheral blood mononuclear cells samples by real‐time reverse transcription‐quantitive polymerase chain reaction assay. HC (healthy controls): the people with negative interferon‐gamma release assay (IGRA)—and no abnormality in lung computed tomography; TB (tuberculosis patients): initial treatment tuberculosis patients with positive IGRA or positive smear examination. The data between these two groups were tested by Student's *t* test. The relative messenger RNA (mRNA) expression levels of Retn (*p* = 0.0502), Amy2a (*p*  = 0.0642), and Sftpd genes (*p* value = 0.2708) were shown as a scatter chart in (A), (B), and (C), respectively.

The transcript expression levels of three genes, Retn, Sftpd, and Amy2a, in 10 TB patients and 15 healthy volunteers were analyzed by RT‐qPCR assay (Figure 5). Compared with the HC group, the relative expression of the Retn gene in TB patients showed a downward trend, and Amy2a gene in TB patients showed an upward trend, which is consistent with the results of the gene expression profile of the mouse TB model. However, the relative expression of the Sftpd gene in the initial treatment TB patients exhibited an upward trend, which is inconsistent with the results of the gene expression profile of the mouse TB model.

Through GEO2R, we verified four GSE data sets. The results were shown as follows: (1) Of the top 20 DE genes in the TB model versus the normal group, 6 DE genes also showed differential expression in GSE89389 and GSE140943 data sets (Table 6), in which the differential expression trend of Sftpd, Mgp, and Retnla was consistent with our results. (2) The expression level of DE gene Ccdc65 decreased significantly after the immunotherapy with *ag85a/b* DNA IM or EP and M72/AS01 (1.535 downregulated in GSE102459 data sets). (3) The differential expression trend of six DE genes in GSE89403 after 4 and 24 weeks of anti‐TB treatment is consistent with that after effective treatment with *ag85a/b* DNA IM or EP (Table 7). The higher expression levels of Cldn5 and Sftpd have positive correlations respectively with 4 weeks or 24 weeks of treatment response. In contrast, the expression level of Mgp was negatively correlated with 4 weeks of treatment response.

**Table 6:** Validation of DE genes in TB model versus normal group by GEO.

Gene symbol	Fold Change values of the DE genes		
	TB model versus normal group	GSE89389	GSE140943
Iapp	832↑	1.104↓	
Sftpd	48↓	1.659↓	
Saa3	20↓	6.615↑	
Mgp	17↓	1.306↓	
Igfbp6	16↓	3.718↑	
Retnla	21↓		1.126↓

Abbreviations: DE, differentially expressed; TB, tuberculosis.

**Table 7:** Multinomial logistics regression of DE genes after 4 and 24 weeks anti‐TB treatment.

Treatment time	Gene symbol	Expression	*B*	Std. error	Wald	Sig.	95% confidence interval	
							Lower bound	Upper bound
4 weeks	Vstm2l↓	Downregulated	−0.171	0.184	0.86	0.354	0.588	1.209
	Chgb↓	Downregulated	0.081	0.11	0.547	0.46	0.874	1.346
	Slc30a8↓	Downregulated	−0.042	0.08	0.282	0.596	0.82	1.121
	Sftpd↑	‐	0.164	0.211	0.6	0.439	0.779	1.782
	Mgp↑	Downregulated	−0.433	0.213	4.138	0.042	0.428	0.984
	Cldn5↑	Upregulated	−0.432	0.21	4.224	0.04	0.43	0.98
24 weeks	Vstm2l↓	Downregulated	−0.059	0.241	0.059	0.808	0.588	1.512
	Chgb↓	Downregulated	0.02	0.127	0.025	0.874	0.796	1.308
	Slc30a8↓	Downregulated	0.091	0.094	0.939	0.333	0.911	1.316
	Sftpd↑	‐	0.929	0.262	12.6	<0.001	1.516	4.228
	Mgp↑	Downregulated	−0.085	0.255	0.111	0.739	0.557	1.514
	Cldn5↑	Upregulated	−0.361	0.235	2.362	0.124	0.44	1.104

*Note*: *p* < 0.05 was considered statistically significant.

Abbreviations: expression, genes expression after anti‐TB treatment in the GSE89403 data set; ↓, expression downregulated after *ag85a/b* DNA treatment; ↑, expression upregulated after *ag85a/b* DNA treatment.

## 4 DISCUSSION

EP can improve the transfer efficiency of plasmid DNA to the nucleus and significantly improve the immunogenicity of DNA vaccines.^75^ In research on animal models of various diseases, the immunogenicity induced by EP is 10 to 100 times higher than that of other immunization methods, and the efficiency of delivering plasmid DNA using EP is 100 to 1000 times higher than that without EP.^76^, ^77^, ^78^, ^79^, ^80^ The clinical trial of DNA vaccines delivered by EP has been used for a variety of diseases, for example, hepatitis B, COVID‐19, influenza, and HIV, and all showed good safety. In the same way, EP can also enhance the immunogenicity of DNA vaccines in humans.^81^ Our previous studies have demonstrated that the *ag85a/b* DNA vaccine especially delivered by EP, had significant efficacy in the treatment of the TB model, which could induce a Th1‐type immune response, and reduce the number of viable bacteria in organs and the degree of organ lesions.^28^, ^31^ The vaccines containing ag85 complexes constructed by other researchers also showed good therapeutic effects on MTB infection.^82^, ^83^ However, the current research on vaccines containing ag85 antigens mainly focused on protective or therapeutic efficacies, and the research on their mechanism was mainly limited to the adaptive immune response of the host, and the target and mechanism of protection and immunotherapy with ag85 vaccine have not been studied through system biology. This study analyzed the effect of different doses of *ag85a/b* DNA vaccine IM or EP immunization on PBMC gene transcriptome by gene expression profiling, and clarified for the first time that *ag85a/b* DNA vaccine had a significant recovery effect on abnormal gene expression and regulatory pathway changes caused by MTB infection, and further revealed the target and mechanism of action of *ag85a/b* DNA vaccine. The exploration of a therapeutic DNA vaccine for tuberculosis may provide a new host‐directed therapy for the clinic.

### 4.1 Positive correlation trend between the dose of *ag85a/b* DNA vaccine and the immune effect

At present, the effective dose of DNA vaccine IM immunized mice is usually 100 μg.^31,84^ The results of our study confirmed that the 100 μg DNA IM could significantly affect the differential expression of host genes and changes in regulatory pathways. The lower than 100 μg DNA vaccine had little effect on differential gene expression and regulatory pathways, and the higher than 100 μg would not lead to more significant effects and make a waste of vaccine inversely. The results suggest that it is necessary to explore the appropriate immunization dose to achieve an effective intervention effect when different animals and humans are IM immunized with DNA vaccines. The method of system biology will be helpful to determine the effective dose in future clinical trials of DNA vaccines.

### 4.2 EP immunization enhances the immune effect of the vaccine

Our previous research showed that only 50 μg of *ag85a/b* DNA vaccine by EP immunization could reach the efficacy of 100 μg DNA IM.^31^ Other results of clinical trials or animal experiments have also demonstrated that DNA vaccines, for example, from HIV,^85^ Zika virus,^86^ Japanese encephalitis virus,^87^ and HPV,^88^ EP immunization could improve the immune effect. From the gene transcriptome level, our study reveals that 10 μg DNA EP immunization could significantly affect the differential expression of genes in the body. With the gradual increase of DNA doses, the effects of DNA vaccine on DE gene numbers, differential expression levels, and regulatory pathways had not changed obviously. But 50 μg DNA EP immunization had the best efficacy, such as the lowest number of bacterial colonies in organs, and the lowest lesion area and degree.^31^ These results suggest that the effect of EP immunization was also related to the DNA dose, but EP immunization could improve the host immune efficiency more than IM immunization. A lower dose of DNA EP could reach the same immune effect as a higher dose of DNA IM, which can reduce the amount of DNA vaccine used. In addition, we also found that the targets of DNA EP were highly consistent with those of DNA IM, and the DE genes and regulatory pathways affected by the two immunization methods of effective DNA doses were highly overlapping (Figure 3). Our study further proves that DNA EP immunization could enhance the host's immune response, but did not change its main targets and mechanism of action.^89^

### 4.3 *Ag85a/b* DNA vaccine therapy reduces the catabolism of tuberculosis

TB is a consumptive disease.^90^ The results of DE gene analysis, GO biological process, and KEGG pathway analyses in this study all proved that the metabolic function of the mouse TB model group had changed greatly and their catabolism had increased. Some DE genes related to pancreatic islet dysfunction (Iapp, Scg2, Chga, Chgb, Slc30a8), carbohydrate and glycogen metabolism (Amy2a5, Gcg, Ins1, Amy1), protein hydrolysis (Try5, Cpa1, Gm13011, Try4, Cpb1), lipid metabolism (Ins1, Pnlip), neuroendocrine (Vstm2l, Resp18) were significantly upregulated, which undoubtedly increased the basic metabolic rate of the mice with MTB infection. However, the state of chronic high consumption will lead to malnutrition of the body and tuberculosis will worsen. We further characterized five highly enriched genes relevant to islet dysfunction in the TB model group. Among them, the most upregulated islet amyloid peptide (IAPP) is a peptide hormone that regulates glucose metabolism synthesized and secreted by islet B cells. At present, studies have found that IAPP aggregation not only had direct toxicity to insulin‐producing B cells but also lead to inflammation and dysfunction of pancreatic B cells by activating NLRP3 inflammasome in infiltrating macrophages.^39^, ^40^ Therefore, IAPP is an important pathological factor that causes type 2 diabetes mellitus (T2DM). Vogt et al. used monoclonal antibody m81 to prevent IAPP accumulation, which could block islet inflammation and delay the onset of T2DM.^91^ Slc30a8 encodes zinc transporter 8 (ZnT8) playing an essential role in zinc homeostasis inside pancreatic B cells and ZnT8 is vital for the biosynthesis and secretion of insulin.^52^ ZnT8 is a minor diabetogenic antigen that can participate in type 1 diabetes mellitus (T1DM) in conditions in which the islet is first made receptive to immunological insults.^92^ In addition, Slc30a8 was identified as a novel T2DM susceptibility gene.^93^ Both our study and the GSE89403 confirm that Slc30a8 expression levels would decrease after anti‐TB treatment, which is beneficial to improve pancreatic dysfunction. Other three genes Scg2 (secretogranin II), Chga, and Chgb (chromogranins A and B), belonging to the chromogranin/secretogranin family,^94^ are considered to have anti‐inflammatory properties, participate in inflammatory reactions, and contribute to host defense.^42^, ^43^ The increase in CHGA has been used as a new biomarker to evaluate the death risk of patients with severe sepsis or coronavirus disease.^43^, ^95^ In addition, CHGA, CHGB, SCG2, and some CHGA cleavage products affect glucose homeostasis and different types of diabetes.^96^ This study found for the first time that the significant increases of these three genes may be related to the inflammatory reaction after MTB infection, and also play important roles in the pathogenesis of various types of diabetes.^96^, ^97^ The current research showed that the risk of pulmonary TB in T2DM patients was about two to three times that of the general population,^98^ and the results of this study suggest that TB may also increase the incidence rate of diabetes. Amy2a5 is an amylase alpha 2 that catalyzes the hydrolysis of starch into sugar, providing energy for the body.^44^ It was reported that Amy2a5 increased in some infectious diseases.^99^ This study is the first report that the expression of Amy2a5 was significantly increased in the mouse TB model, but only showed an increasing trend in the newly‐treated TB patients, which may be caused by the fact that the catabolism of newly‐treated TB patients was not very severe. After immunotherapy with the *ag85a/b* DNA vaccine IM or EP, the metabolism of sugar, protein, and lipid in mice was reduced, and the state of high metabolism was corrected. The expressions of genes IAPP, Slc30a8, Scg2, Chga, and Chgb were significantly reduced, which can protect islet B cells from apoptosis and can also change the insulin secretion defect caused by the inflammatory environment of pancreatic islets, thus improving the function of islet B cells and reducing the risk of TB patients complicated with T2DM.^39^, ^97^

### 4.4 *Ag85a/b* DNA vaccine therapy improves anabolism of tuberculosis

In the TB model group, most of the downregulated DE genes were related to cell structural proteins and cell functional proteins, and the functional proteins of alveolar epithelial cells account for a large proportion, such as Sftpc, Sftpb, Sftpd, Sftpa1, Mgp, Wfdc2, Sec1413, Postn, Cldn5, Aqp5, Emp2, Foxf1, and so forth. Surfactant protein A (SFTPA, encoded by two homologous genes Sftpa1 and Sftpa2), B (SFTPB), C (SFTPC), and D (SFTPD), secreted by alveolar epithelial cell type II, are key elements of the innate immune system to maintain normal alveolar structure and function and resist MTB infection.^100^, ^101^ Among them, SFTPB and SFTPC play a role in reducing surface tension, and SFTPC also plays an immunomodulatory role in clearing lung infection.^102^ SFTPA and SFTPD are the host defense lectins, which participate in the innate immune response in the lung and enhance the phagocytosis of macrophages on MTB through interaction with alveolar macrophages,^58^, ^103^ thereby enhancing microbial clearance and regulating inflammation. SFTPA and SFTPD also regulate the functions of dendritic cells and T cells.^104^ Sftpa, Sftpd, and Sftpc gene polymorphisms not only increase the risk of TB but also may affect the host's immune response to MTB.^105^ Thacker VV et al.^106^ showed that the decreased expression of alveolar epithelial cells type II markers (Abca3, Sftpa, Sftpb, Sftpc, Sftpd) and type I markers (Aqp5 and Pdpn) would lead to the rapid growth of MTB in macrophages and alveolar epithelial cells.^107^ The growth of MTB in these two cells could be inhibited by the exogenous addition of Curosurf (surfactant substitute of phospholipid and hydrophobic protein). Mgp, a vitamin K‐dependent inhibitor of calcification, may play an anti‐inflammatory role in monocytes and macrophages.^108^, ^109^ Claudin‐5 (Cldn5), a tight junction protein, is mainly expressed by the vascular endothelium, especially expressed strongly in the endothelium of normal lungs.^110^ The expression levels of Cldn5 were significantly decreased in various lung diseases, such as Covid‐19,^111^ chronic obstructive pulmonary disease,^112^ and lung injury,^113^ which induced damage to the pulmonary endothelial barrier. Induction of Cldn5 expression has become a therapeutic strategy for these diseases.^113^ Both our study and GSE89403 found that MTB infection significantly reduced the expression of Sftpd and Cldn5, but the treatment of *ag85a/b* DNA vaccine and GSE89403 significantly increased the expression of Sftpd and Cldn5, proving that Sftpd and Cldn5 can also become the targets for TB treatment. Both our study and the GSE89839 showed that Mgp expression decreased after MTB infection, which may affect the anti‐inflammatory effect of mice. However, after the treatment of *ag85a/b* DNA vaccine and GSE89403, the expression of Mgp was significantly increased, suggesting that the anti‐inflammatory effect of mice may be improved. The mouse Retnla (human Retn), a member of the resistin family, is a secreted protein rich in cysteine. It is not only a protein related to insulin resistance but also a proinflammatory molecule.^67^ Retnla is a negative regulator of Th2‐mediated pneumonia. Retnla^−/−^ mice developed exacerbated lung inflammation compared with their wild‐type controls.^68^ In this study, the expression of Retnla was significantly downregulated in the mouse TB model, which was consistent with the results from two GSE data sets. In TB patients, Retn gene expression also showed a downward trend. The downregulation of Retnla (Retn) expression caused Th1 immune response to shift to Th2 immune response, while the immunotherapy of the *ag85a/b* DNA vaccine significantly increased Retnla expression, which was conducive to correcting Th1/Th2 immune imbalance.^67^ After the immunotherapy with *ag85a/b* DNA vaccine IM or EP in the mice infected with MTB, the anabolism, developmental process, and immune response‐related pathways (such as ECM receiver interaction, Focal induction, PI3K Akt signaling pathway, Rap1 signaling pathway, etc.) were enhanced, the transcriptional levels of the surfactant genes were significantly upregulated, the number of MTB colonies in the lung was reduced, and the lung lesions in mice were alleviated, which proved that pulmonary surfactants have a potential role in the host‐directed treatment of TB.^107^ The mechanism may be that surfactants can inhibit the growth of MTB by changing the interaction between MTB and host cells.^114^ In addition, surfactants can remove the virulence‐related proteins and lipids on the surface of MTB, and can wrap bacteria, so that they are not easy to infect host cells. Therefore, *ag85a/b* DNA vaccine IM or EP immunization can improve the immune response, eliminate MTB, and then correct metabolic disorders in mice.

## 5 CONCLUSION

MTB infection caused significant upregulation of catabolism‐related DE genes, GO biological processes, and signal pathways in mice, and significant downregulation of anabolism‐related DE genes, GO biological processes, and signal pathways, as well as significant downregulation of multiple immune‐related pathways. Our study found for the first time that the effective doses of *ag85a/b* DNA vaccine immunized whether by IM or EP could significantly upregulate immune‐related pathways, and recover the metabolic disorder and the injury caused by MTB. The action target and mechanism of two effective treatment groups (100 μg DNA IM group and 50 μg DNA EP group) are highly consistent. These findings provide a basis for further elucidating the immunotherapeutic target and mechanism of the *ag85a/b* DNA vaccine.

## AUTHOR CONTRIBUTIONS

**Nan Wang**: Data curation; formal analysis; validation; writing—original draft; writing—review and editing. **Yan Liang**: Methodology; project administration; supervision; writing—review & editing. **Qianqian Ma**: Validation. **Jie Mi**: Validation. **Yong Xue**: Methodology; validation. **Yourong Yang**: Resources; supervision; validation. **Lan Wang**: Validation. **Xueqiong Wu**: Data curation; funding acquisition; project administration; supervision; writing—review & editing.

## CONFLICT OF INTEREST STATEMENT

The authors declare no conflict of interest.

## ETHICS STATEMENT

Ninety‐six 6–8‐week age of the specific pathogen‐free (SPF) female BALB/c mice were purchased from Beijing Vital River Laboratory Animal Technology Company Limited (Beijing, China), placed and fed under infection barrier conditions in a negative pressure animal room in the Eighth Medical Center of the Chinese PLA General Hospital (Beijing, China). Animal experiments strictly followed the rules and regulations of the PLA General Hospital for the management and use of laboratory animals. After the whole blood from 10 confirmed TB patients and 15 healthy people recruited in the Eighth Medical Center of the Chinese PLA General Hospital were routinely tested, the remaining whole blood samples were used to perform RT‐qPCR verification. The study was approved respectively by the Animal Ethical Committee and the Medical Ethics Committee of the Eighth Medical Center of PLA General Hospital (approval 202209151022).

## Supporting information

Supporting information.

Supporting information.

Supporting information.

Supporting information.

Supporting information.

Supporting information.

Supporting information.

Supporting information.

## Data Availability

The authors confirm that the data supporting the findings of this study are available within the article and its supplementary materials.
