# Supplementary material for: Mechanisms of ag85a/b DNA vaccine conferred immunotherapy and recovery from Mycobacterium tuberculosis‐induced injury
Source: Immun Inflamm Dis. 2023 May 16;11(5):e854. doi: 10.1002/iid3.854 (PMC10187016; doi:10.1002/iid3.854)
Supplement: Supplementary file 1 — Supporting information. [file IID3-11-e854-s001.docx]

A B C


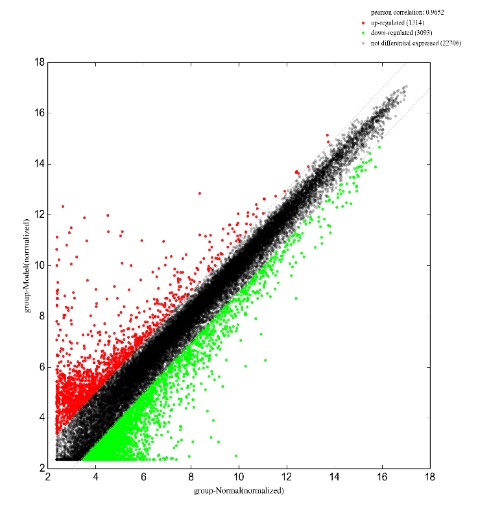

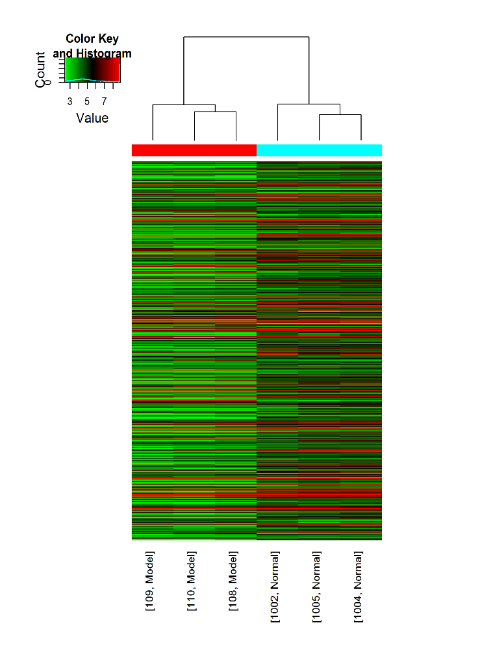

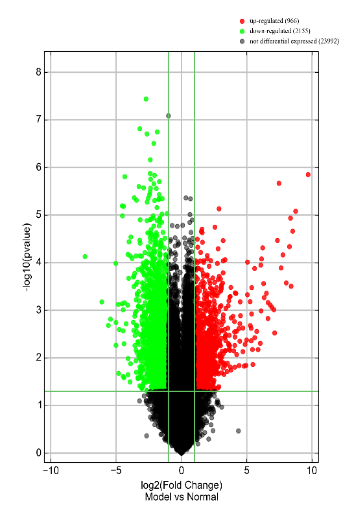


Normal control

TB Model

-Log10(*p* value)

Log2(Fold Change)

TB Model

Normal

(a)


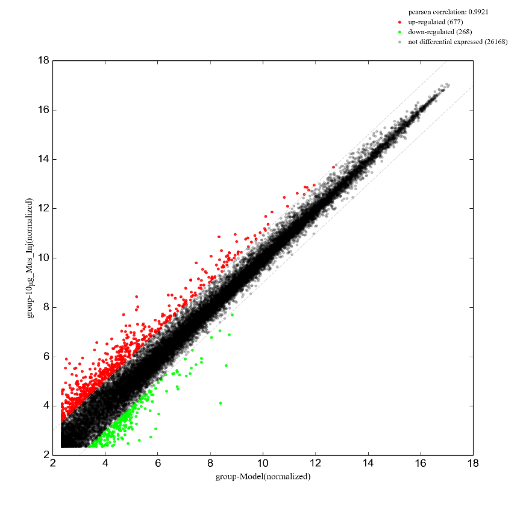

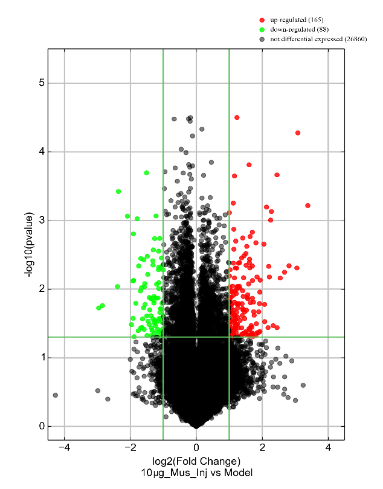

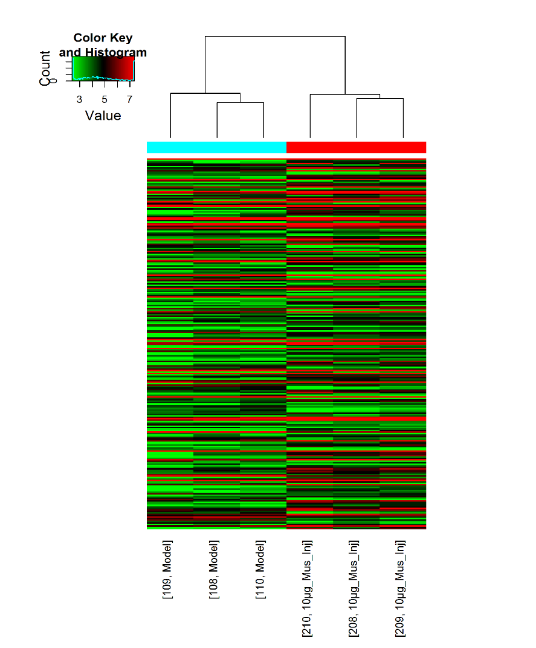


10μg DNA IM

TB Model

Log2(Fold Change)

-Log10(*p* value)

TB Model

10μg IM

(b)

TB Model


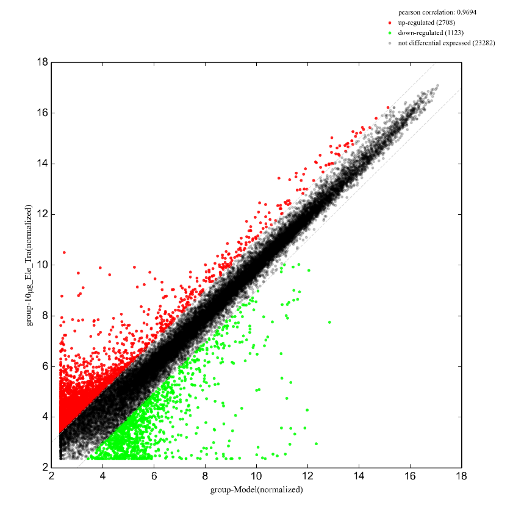


10μg DNA EP

TB Model


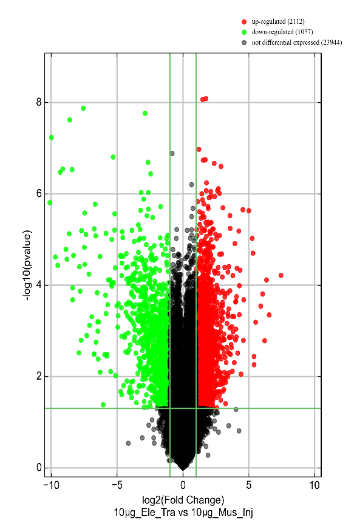


Log2(Fold Change)

-Log10(*p* value)


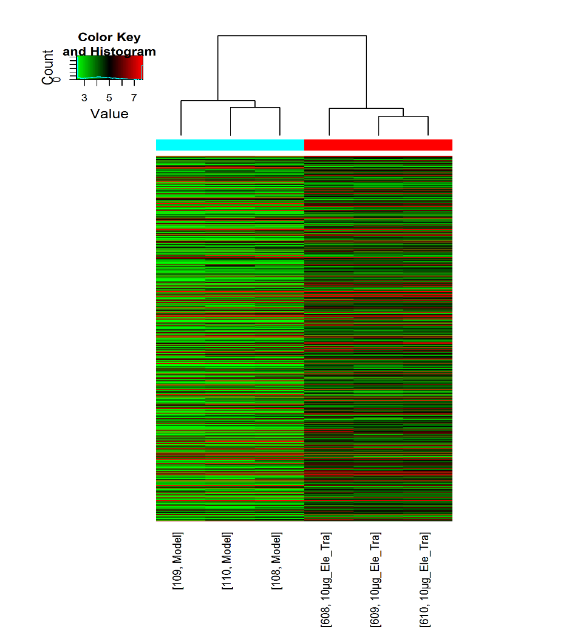


10μg EP

(c)


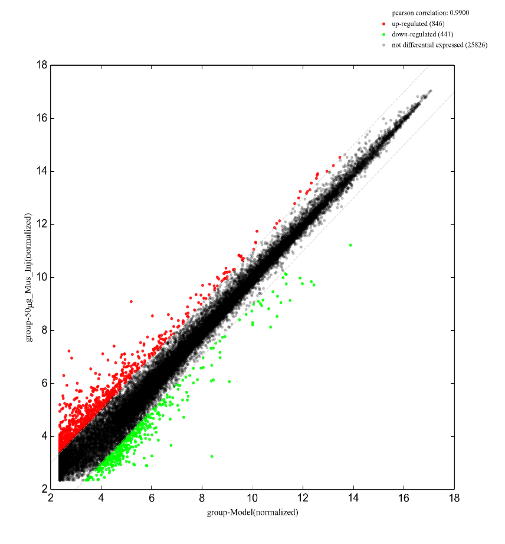

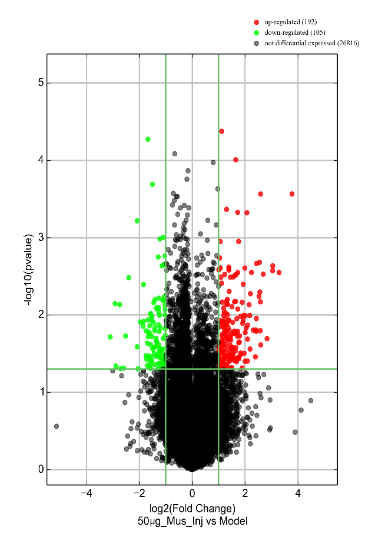

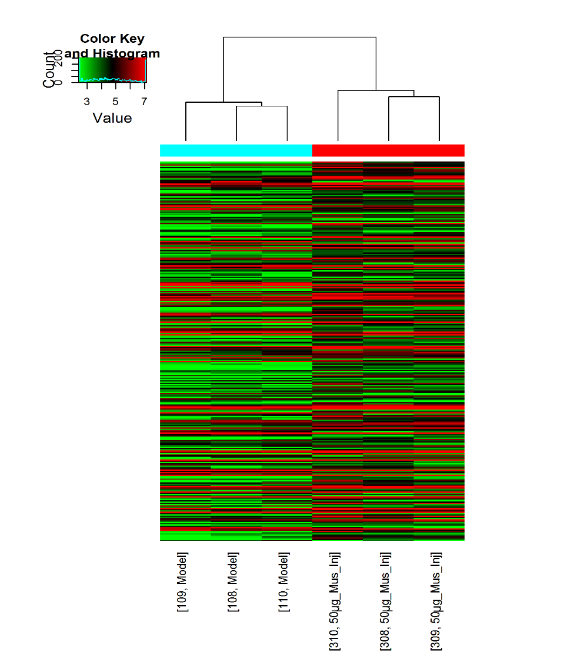


TB Model

50μg DNA IM

-Log10(*p* value)

Log2(Fold Change)

Log2(Fold Change)

TB Model

50μg IM

(d)


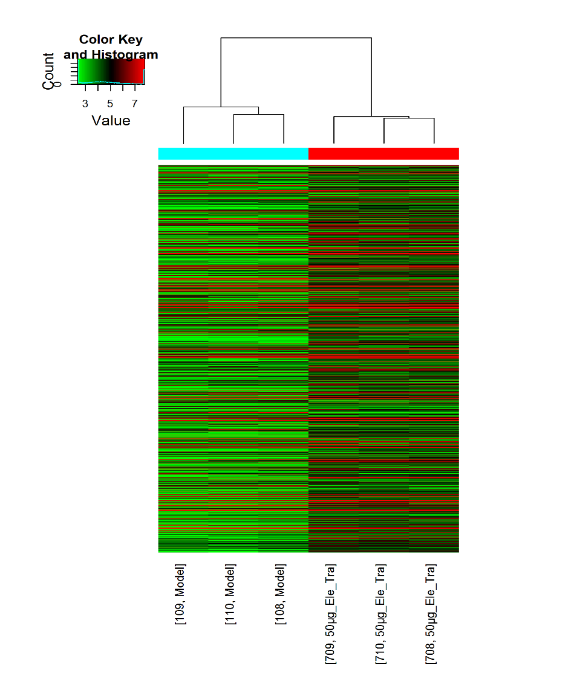


-Log10(*p* value)


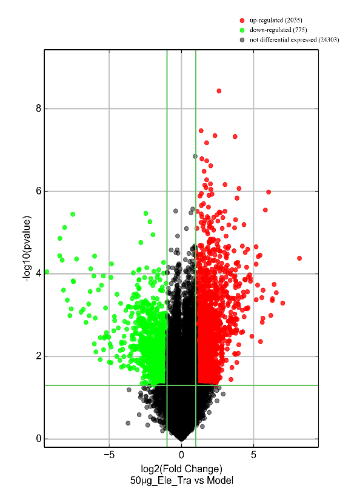


Log2(Fold Change)


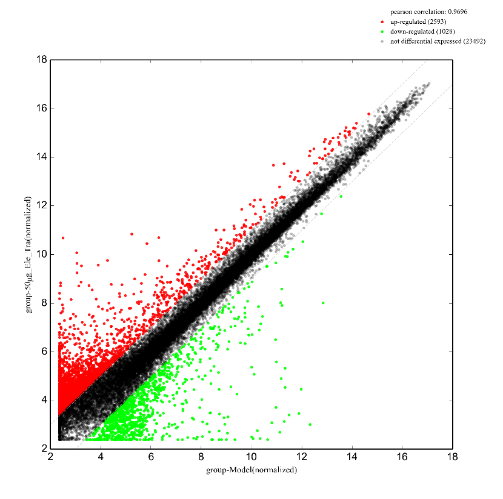


TB Model

50μg DNA EP

TB Model

50μg EP

(e)


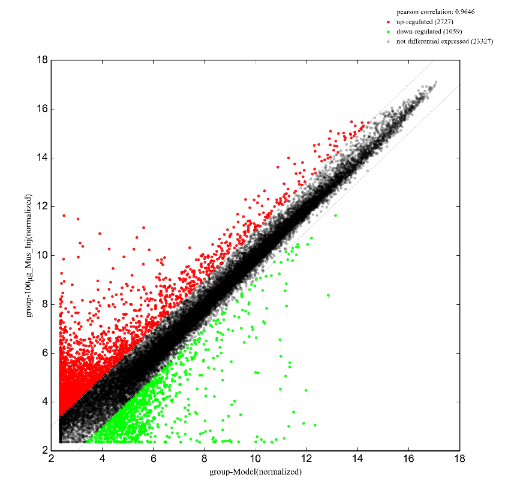

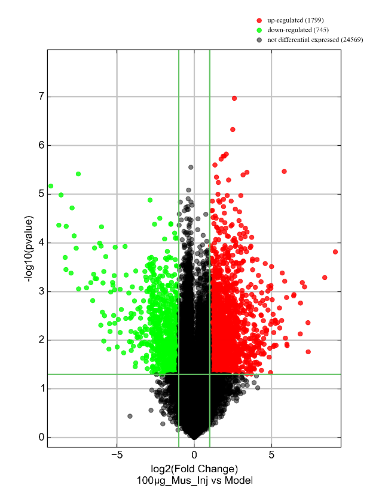

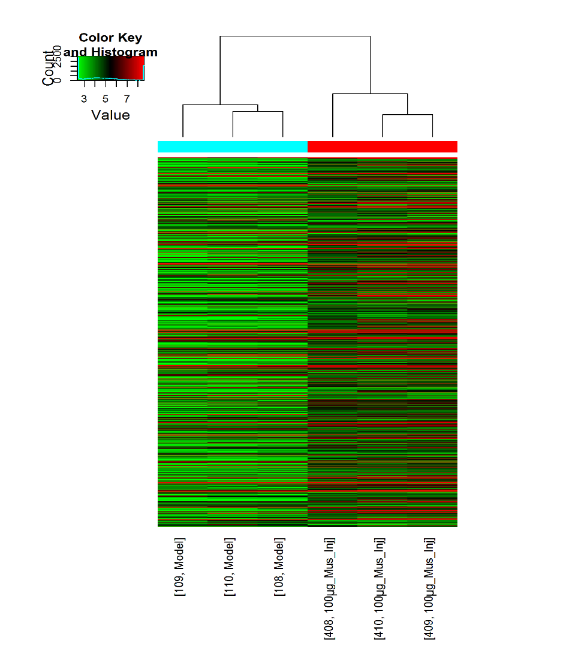


100μg DNA IM

-Log10(*p* value)

Log2(Fold Change)

TB Model

TB Model

100μg IM

(f)


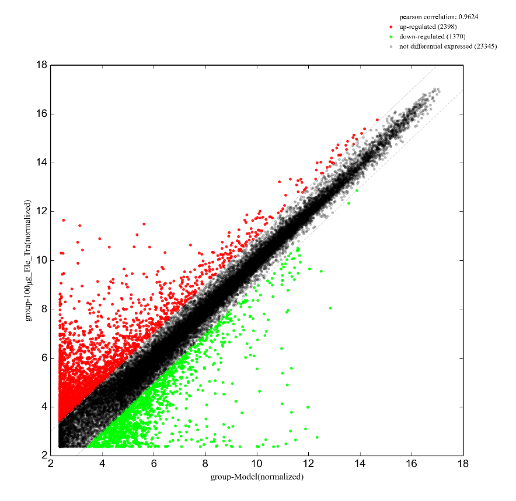


100μg DNA EP

TB Model


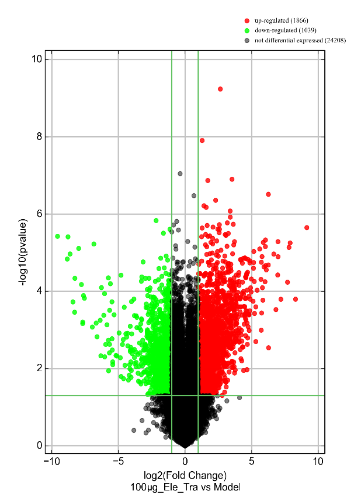


Log2(Fold Change)

-Log10(*p* value)


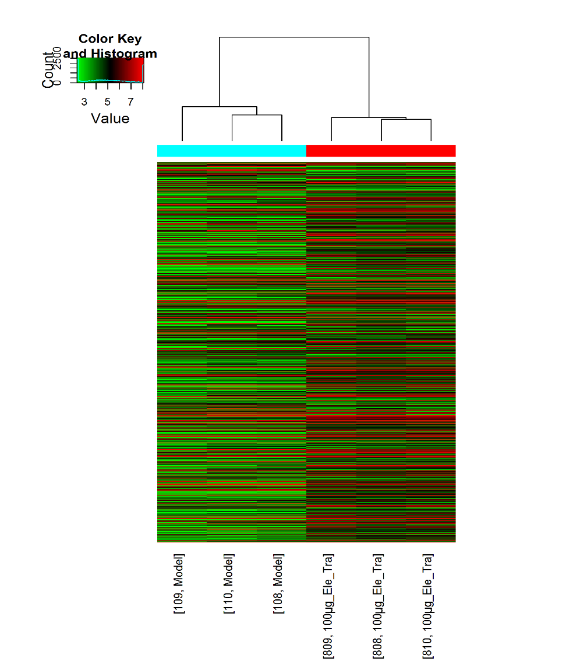


TB Model

100 μg EP

(g)


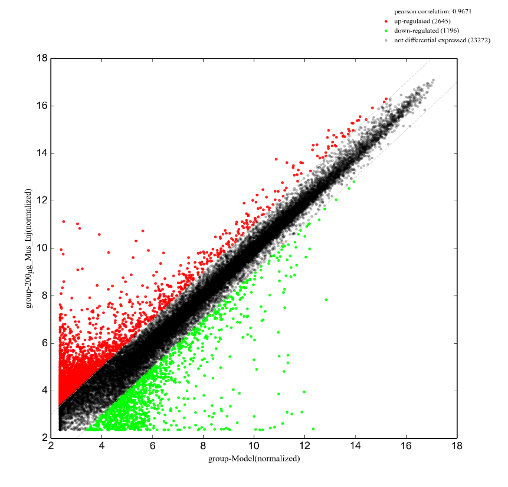

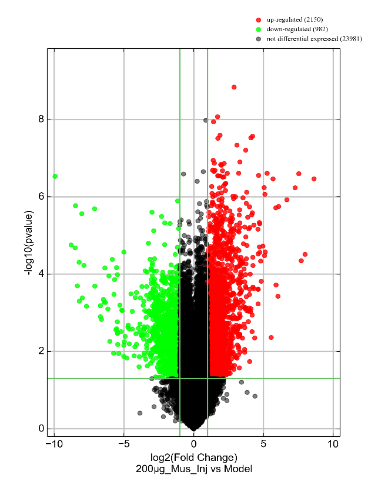

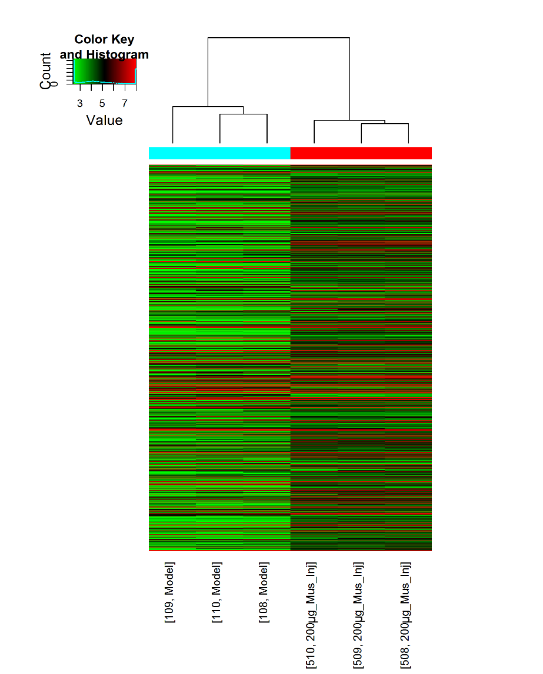


200μg DNA IM

TB Model

Log2(Fold Change)

-Log10(*p* value)

TB Model

200 μg IM

(h)


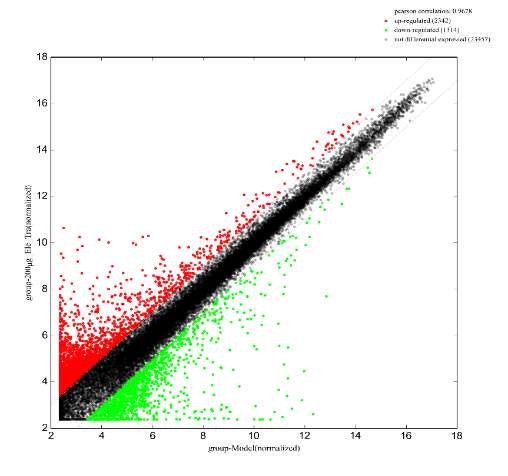


200μg DNA EP

TB Model


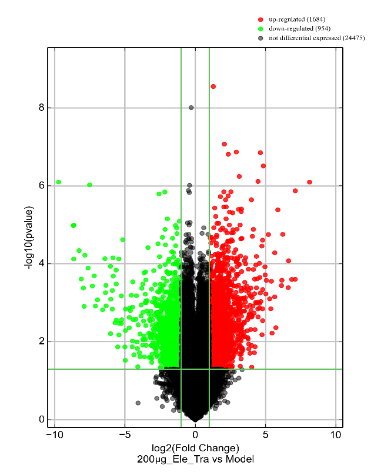


Log2(Fold Change)

-Log10(*p* value)


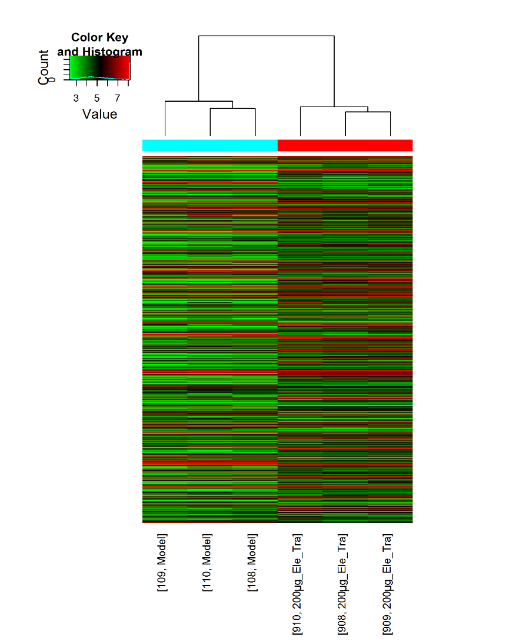


TB Model

200 μg EP

(i)

**Supplementary Figure 1. Scatter-Plot, Volcano plots, and hierarchical clustering visualized the gene expression variation between the TB model group and the normal group or each *ag85a/b* DNA vaccine group and TB model group.**

(A) Scatter-Plot, the values of X and Y axes in the Scatter-Plot are the averaged normalized signal values of the normal mouse group, mouse TB model group, 10, 50, 100, 200μg IM groups, or 10, 50, 100, 200μg EP groups (log2 scaled). The gray lines are Fold Change Lines (the default fold change value given is 2.0). The genes above the top gray line and below the bottom gray line indicated more than a 2-fold change of genes between the two groups (n = 3).

(B) Volcano plots, constructed by using fold-change values and P-values. The vertical green lines correspond to 2.0 fold up and down, respectively, and the horizontal green line represents a P-value of 0.05.

(C) Hierarchical clustering map, cluster analysis arranges samples into groups based on their expression levels. The dendrogram shows the comparison of gene expression differences between the two groups (3 samples). “Red” indicates the up-regulated expression of DE genes with statistical significance, and “green” indicates the down-regulated expression of DE genes with statistical significance.
