# Supplementary material for: Mechanisms of ag85a/b DNA vaccine conferred immunotherapy and recovery from Mycobacterium tuberculosis‐induced injury
Source: Immun Inflamm Dis. 2023 May 16;11(5):e854. doi: 10.1002/iid3.854 (PMC10187016; doi:10.1002/iid3.854)
Supplement: Supplementary file 2 — Supporting information. [file IID3-11-e854-s006.docx]

Up-regulated DE genes Down-regulated DE genes


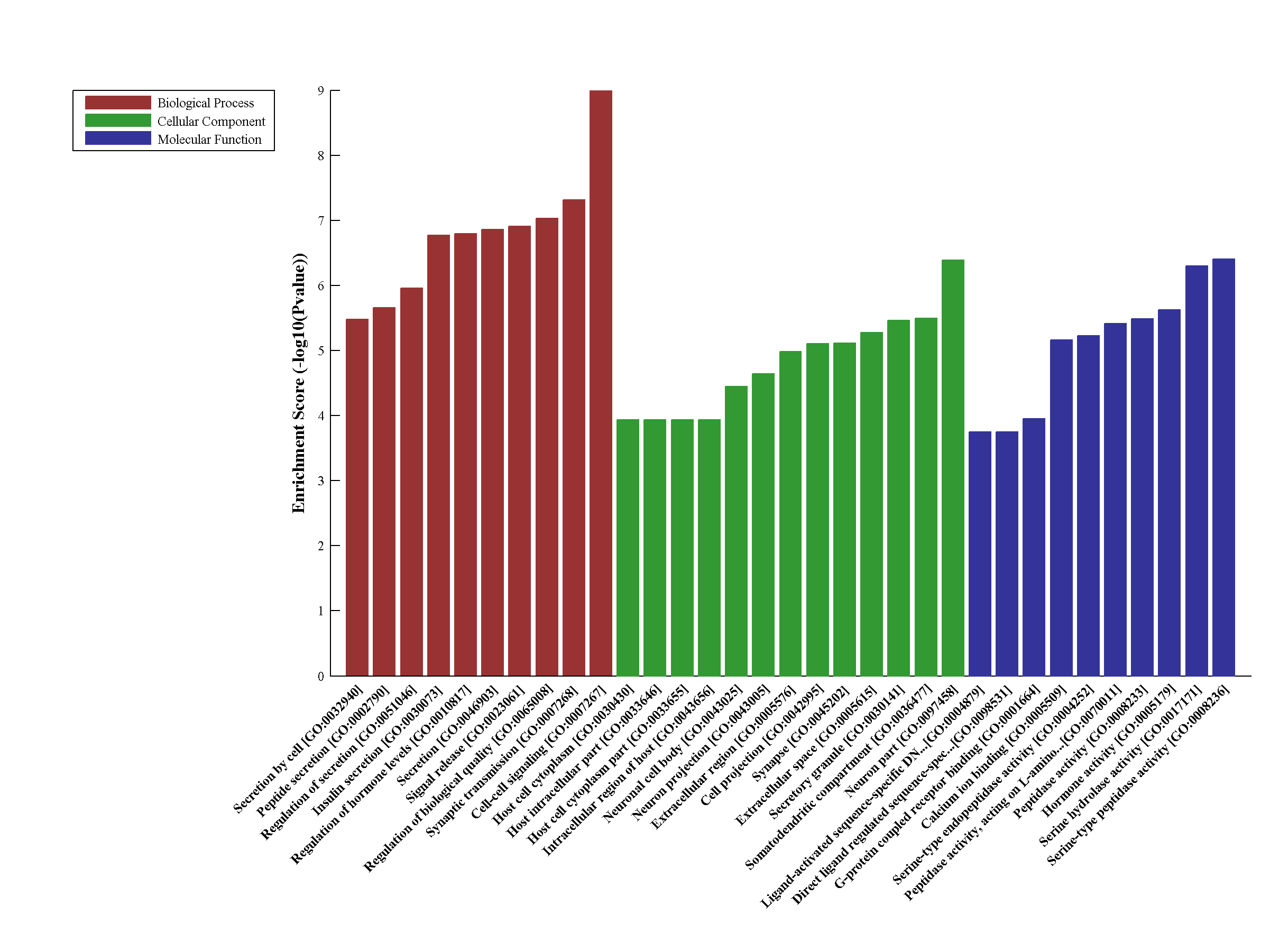

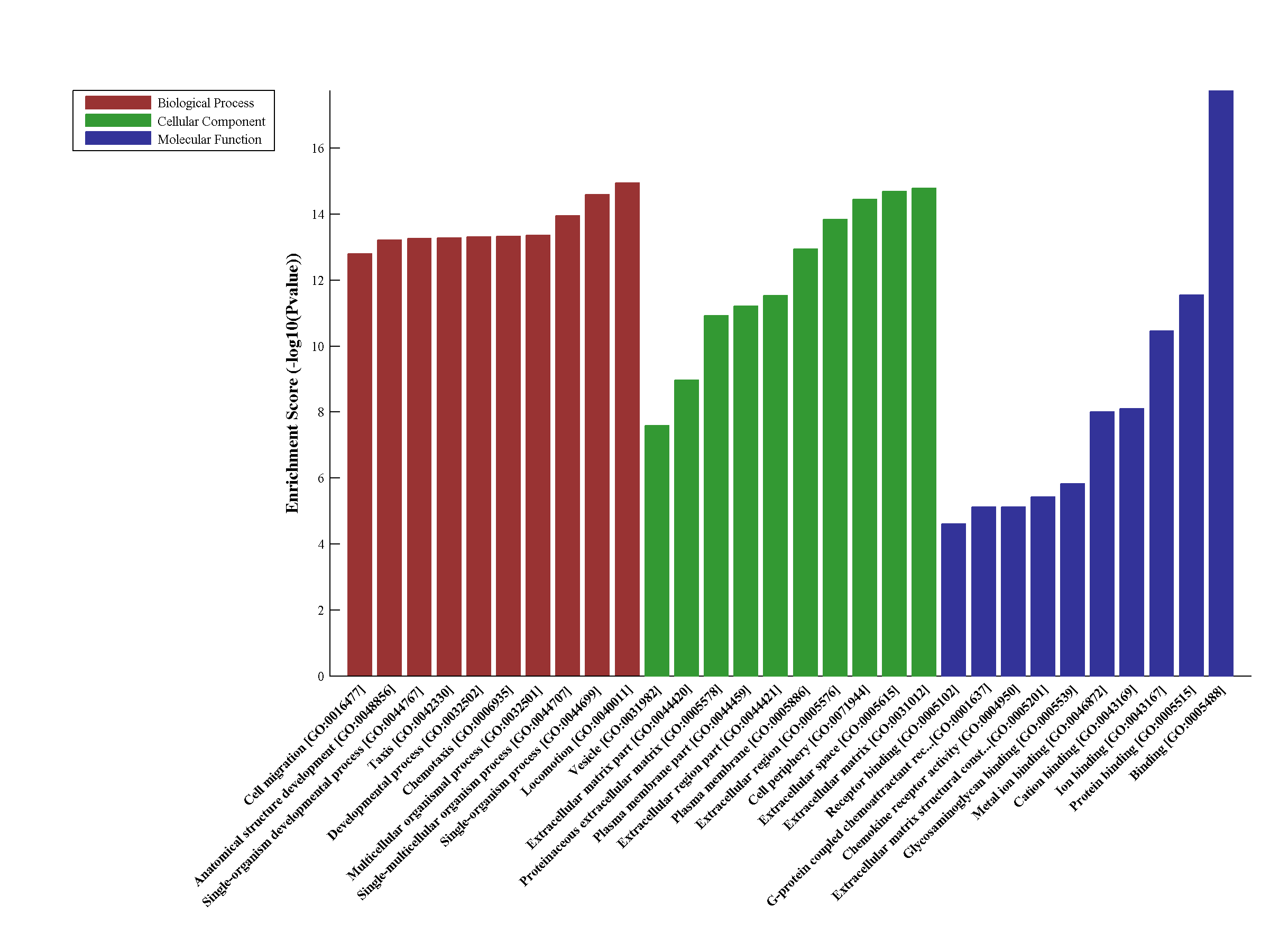


TB model group vs Normal group

A B

Down-regulated DE genes Up-regulated DE genes

10μg DNA IM group vs TB model group


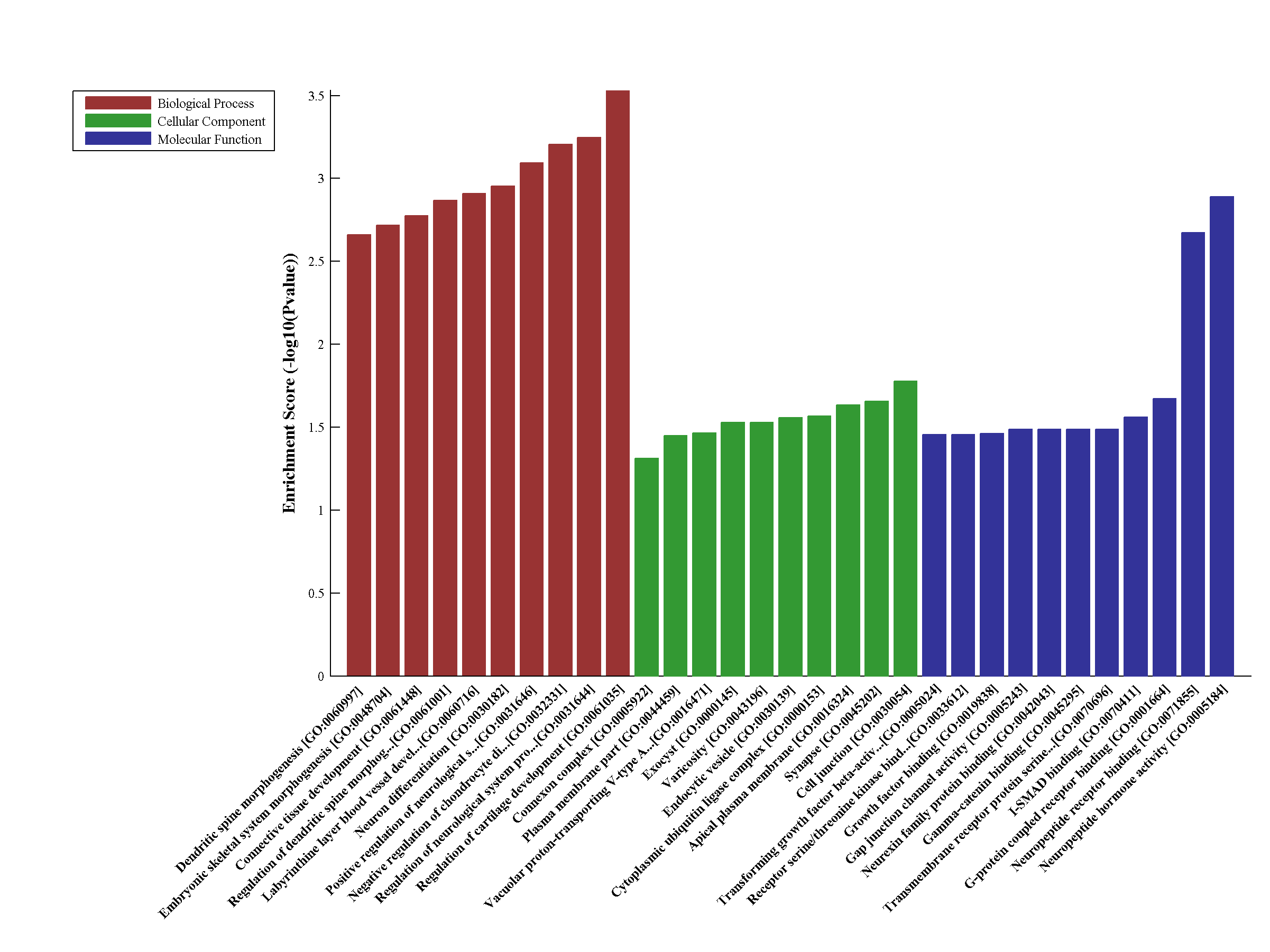

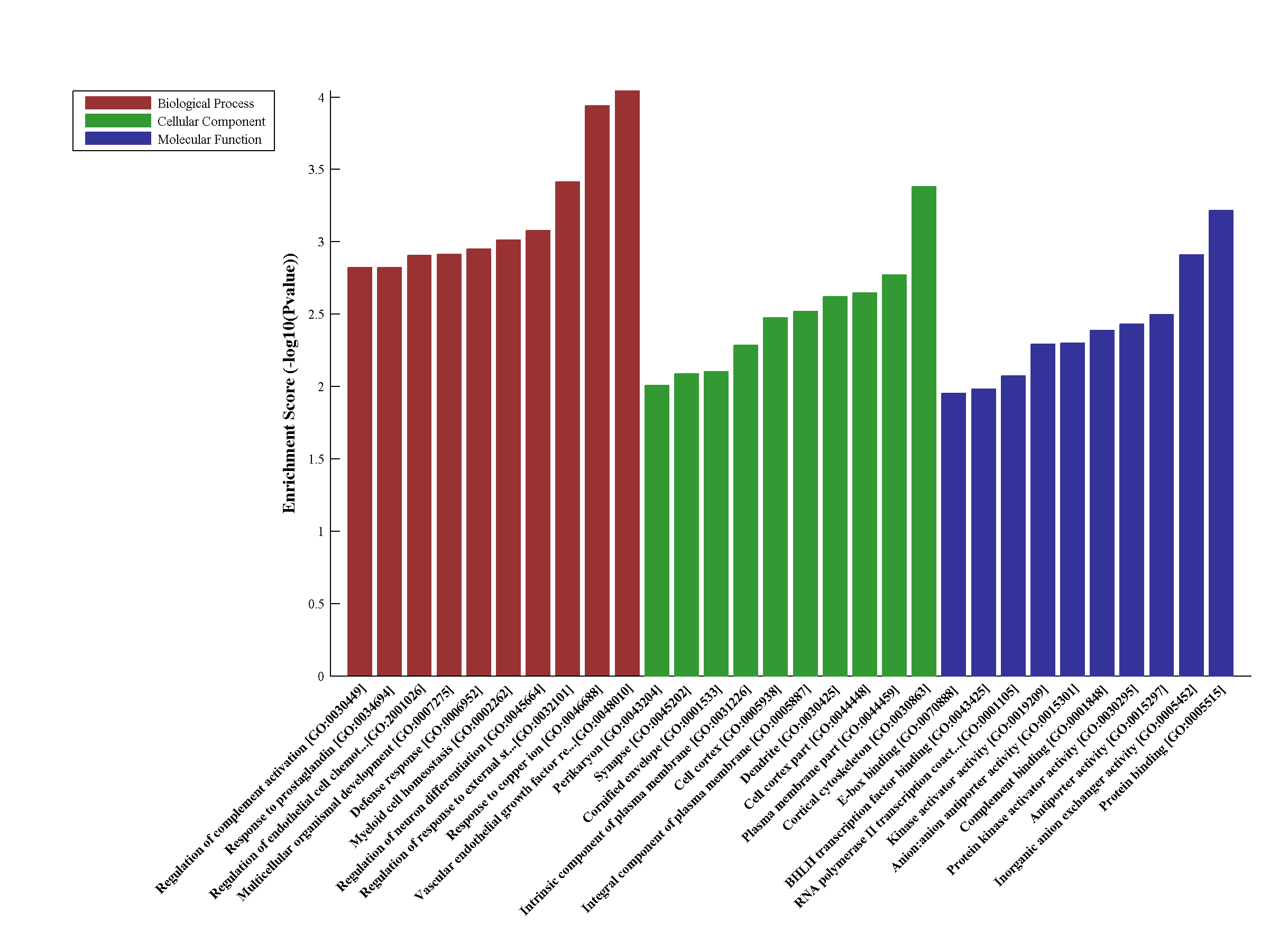


C D

50μg DNA IM group vs TB model group


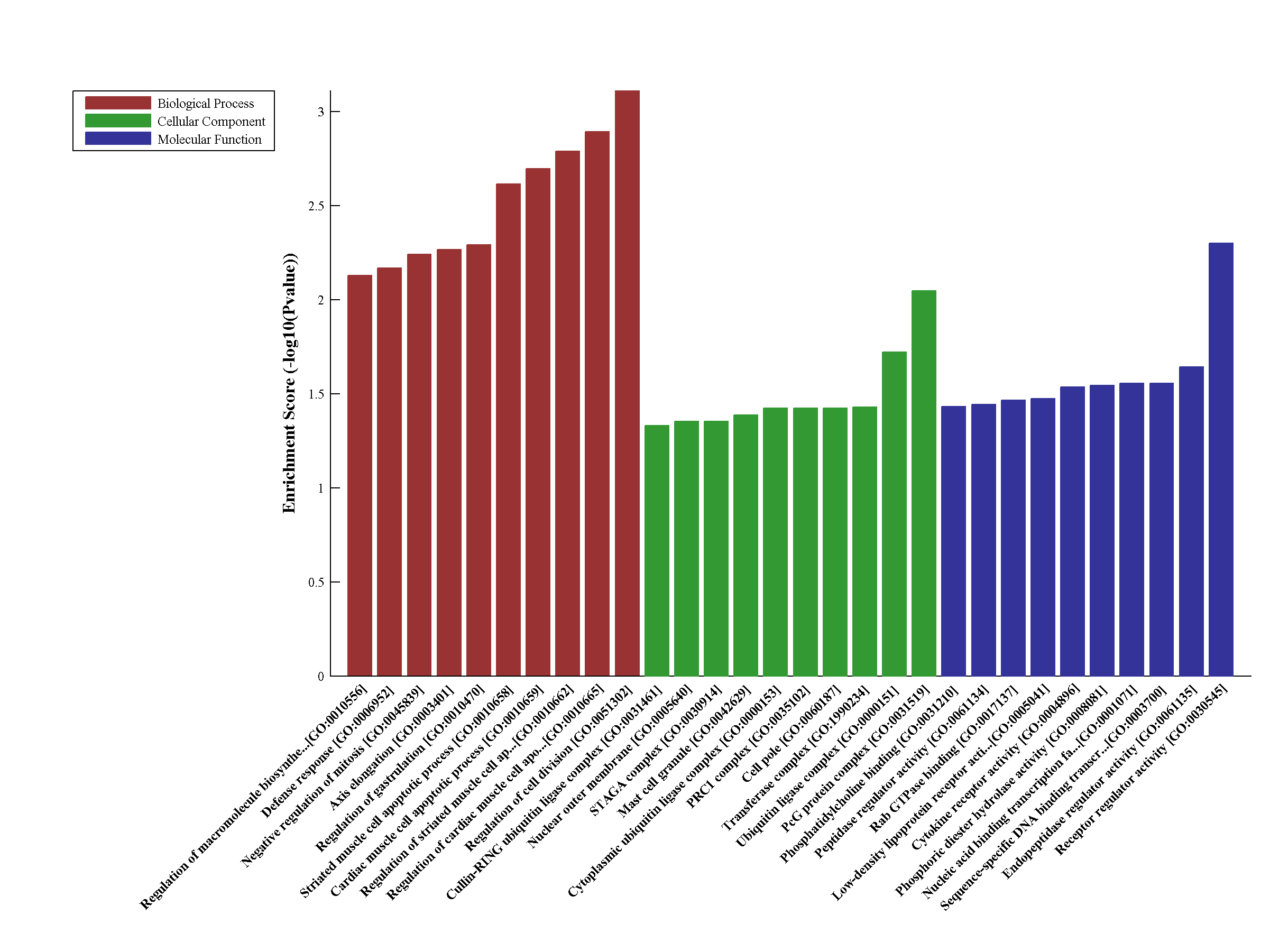

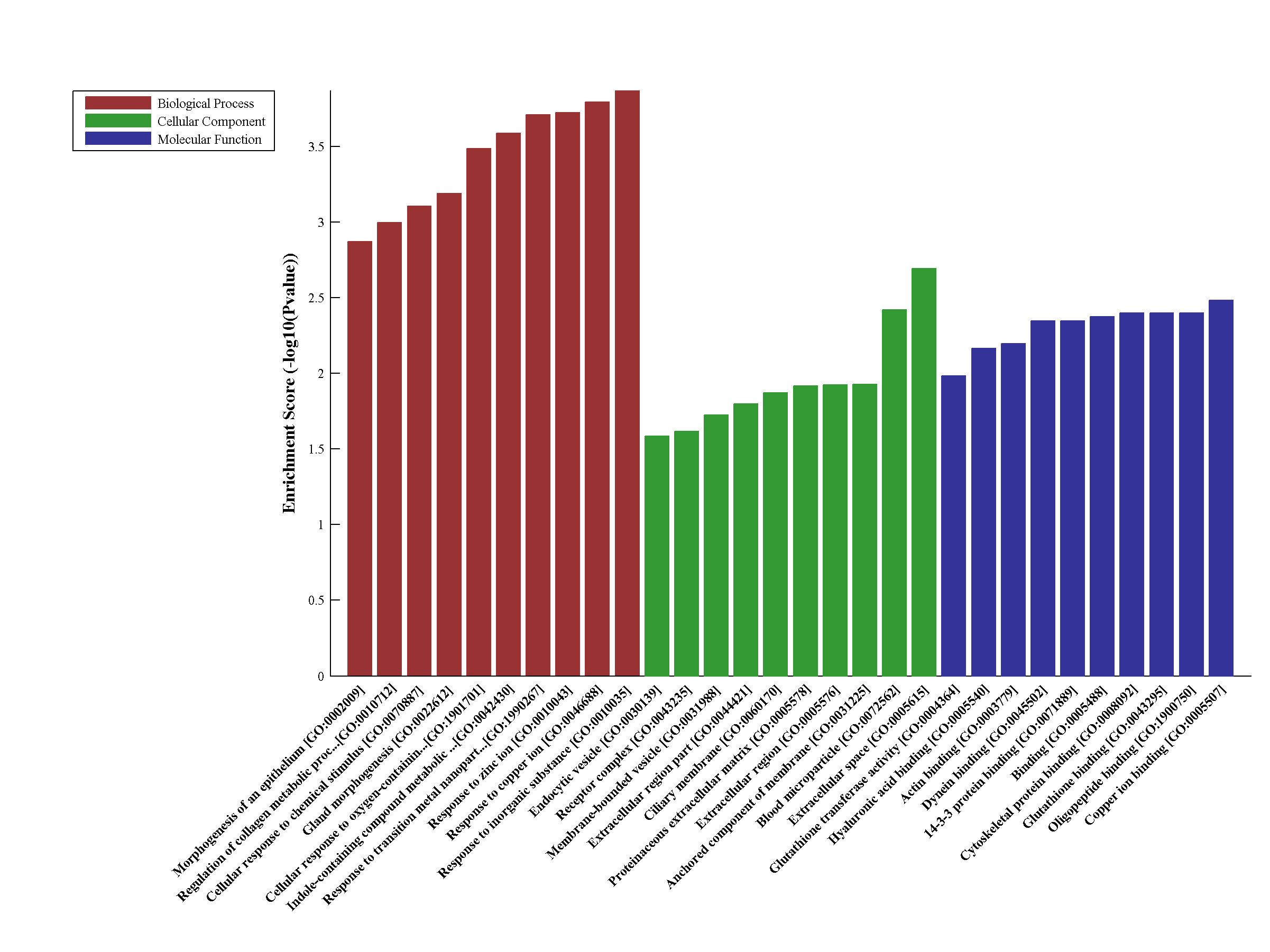


E F

100μg DNA IM group vs TB model group


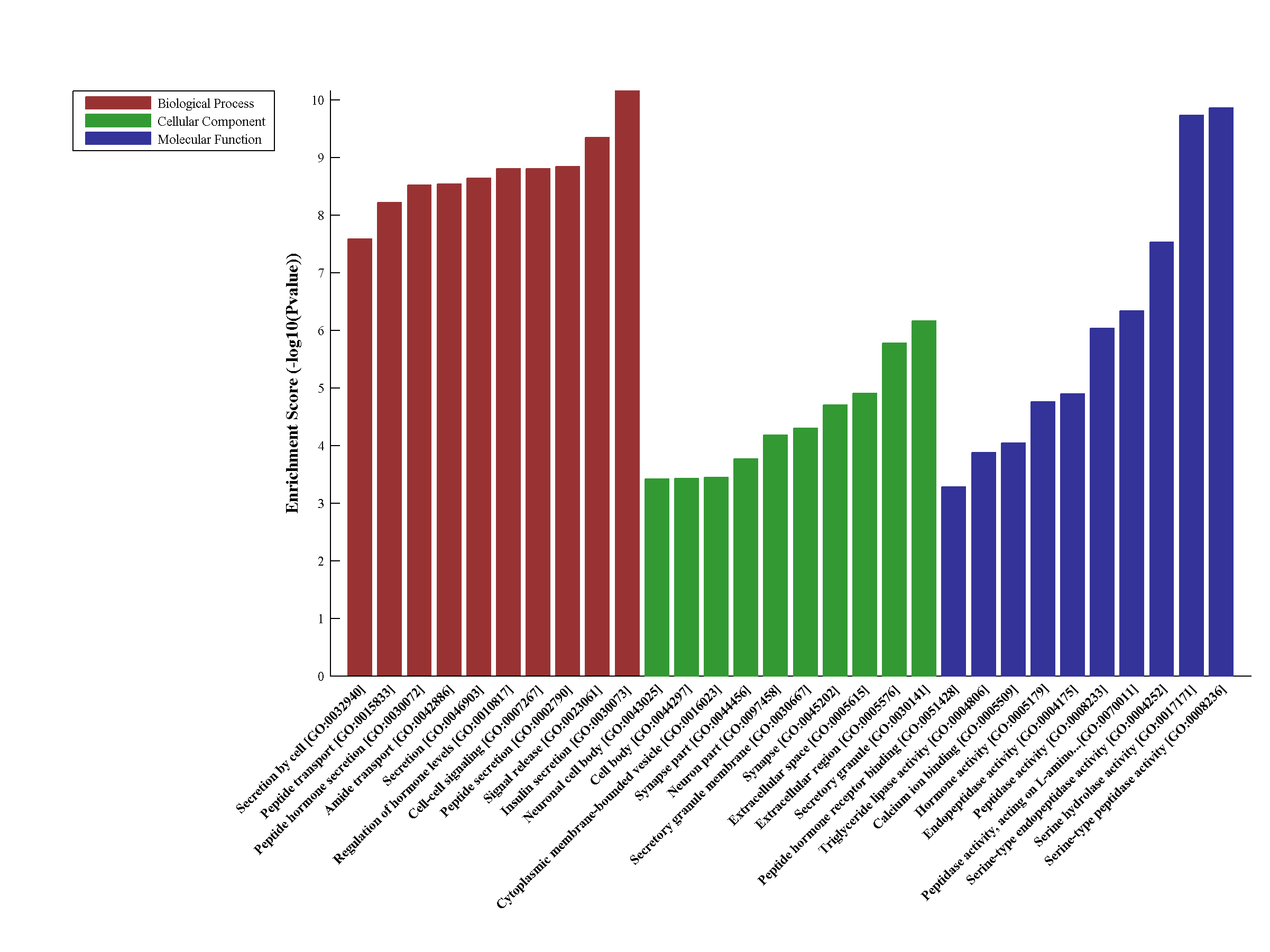

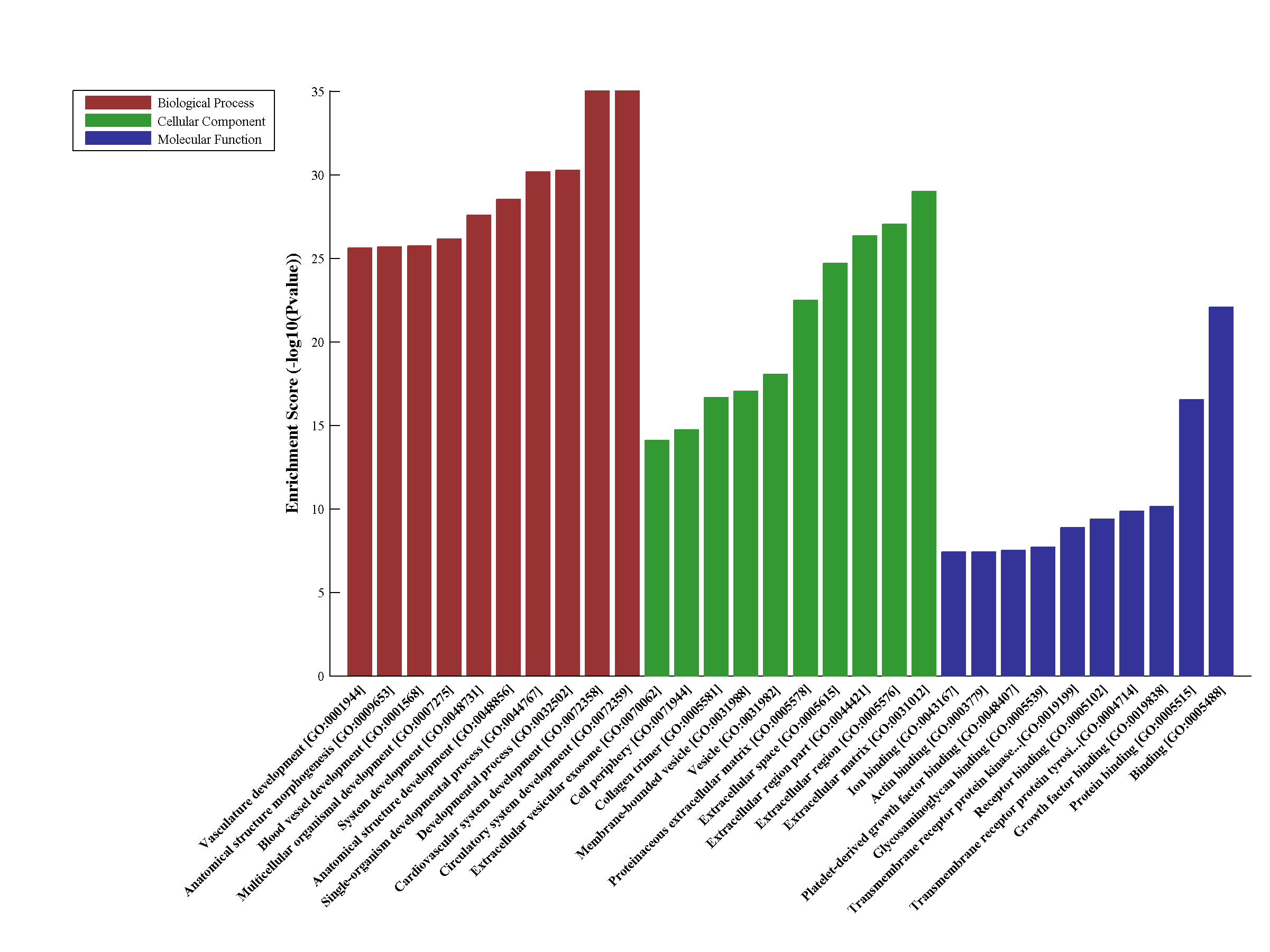


G H


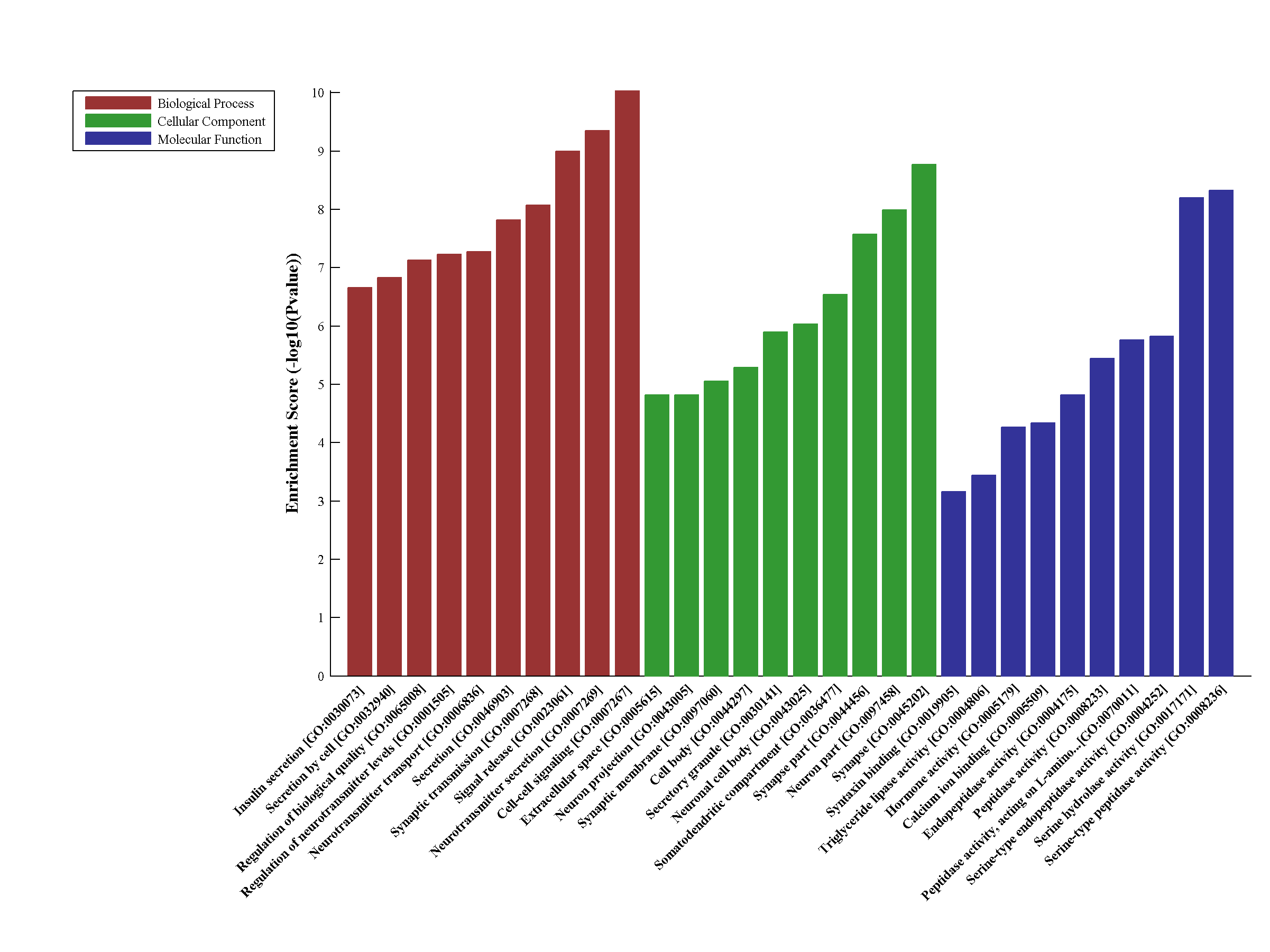

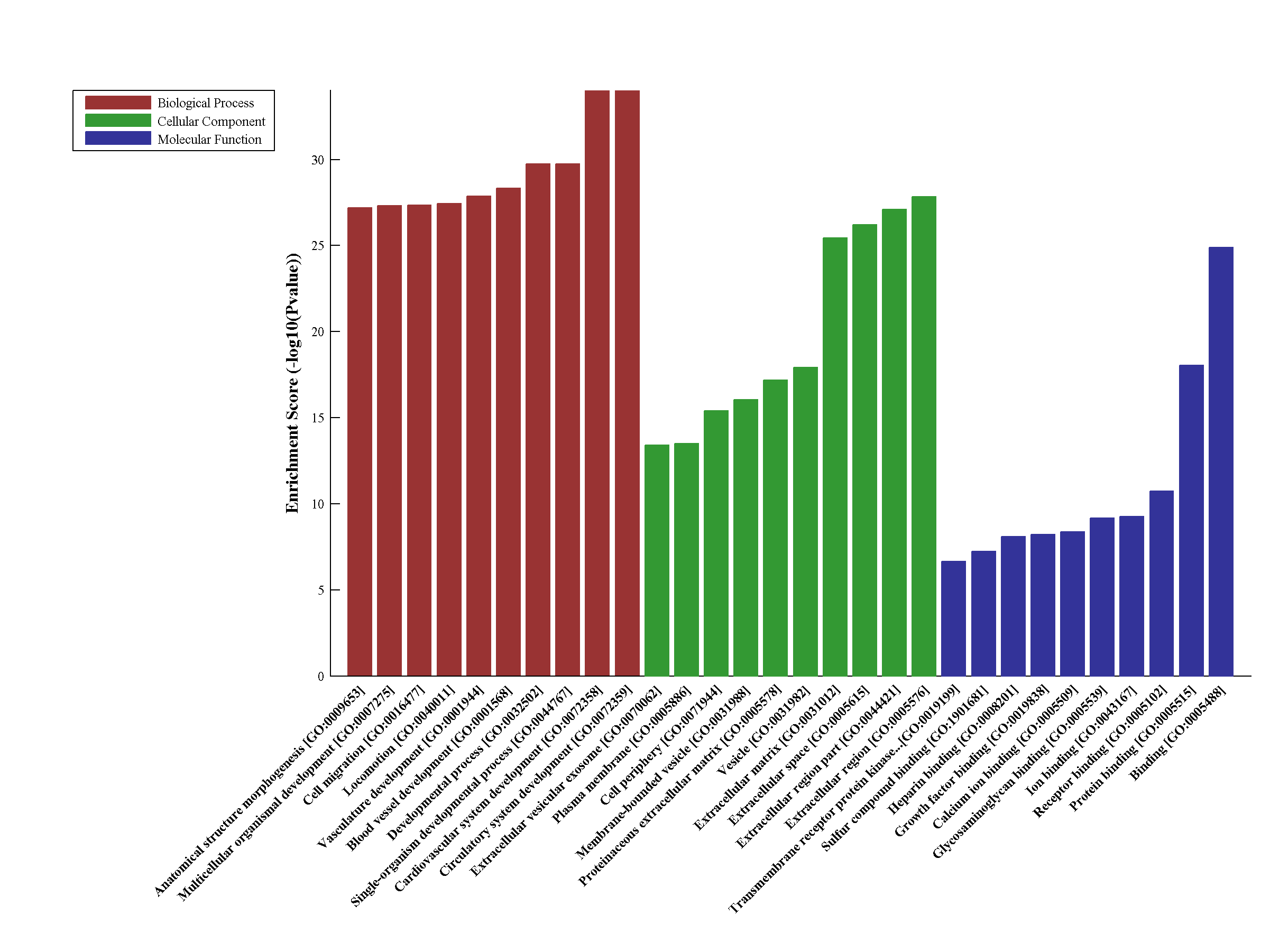


200μg DNA IM group vs TB model group

I J

10μg DNA EP group vs TB model group


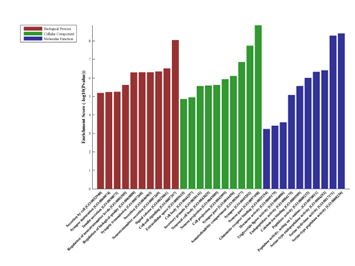

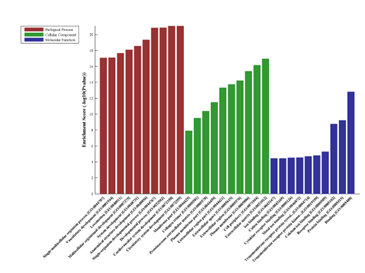


K L


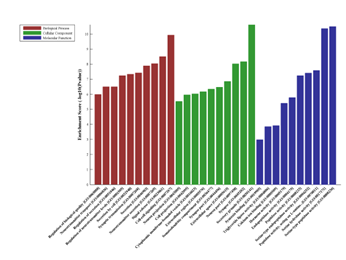

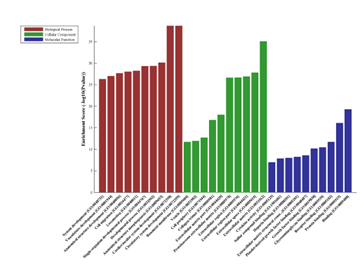


50μg DNA EP group vs TB model group

M N


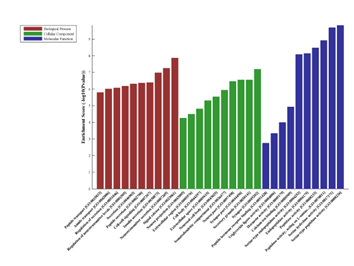

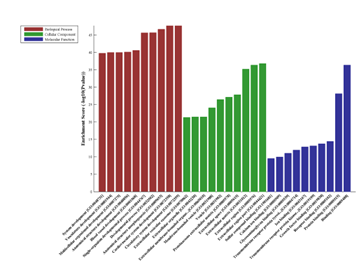


100μg DNA EP group vs TB model group

O P


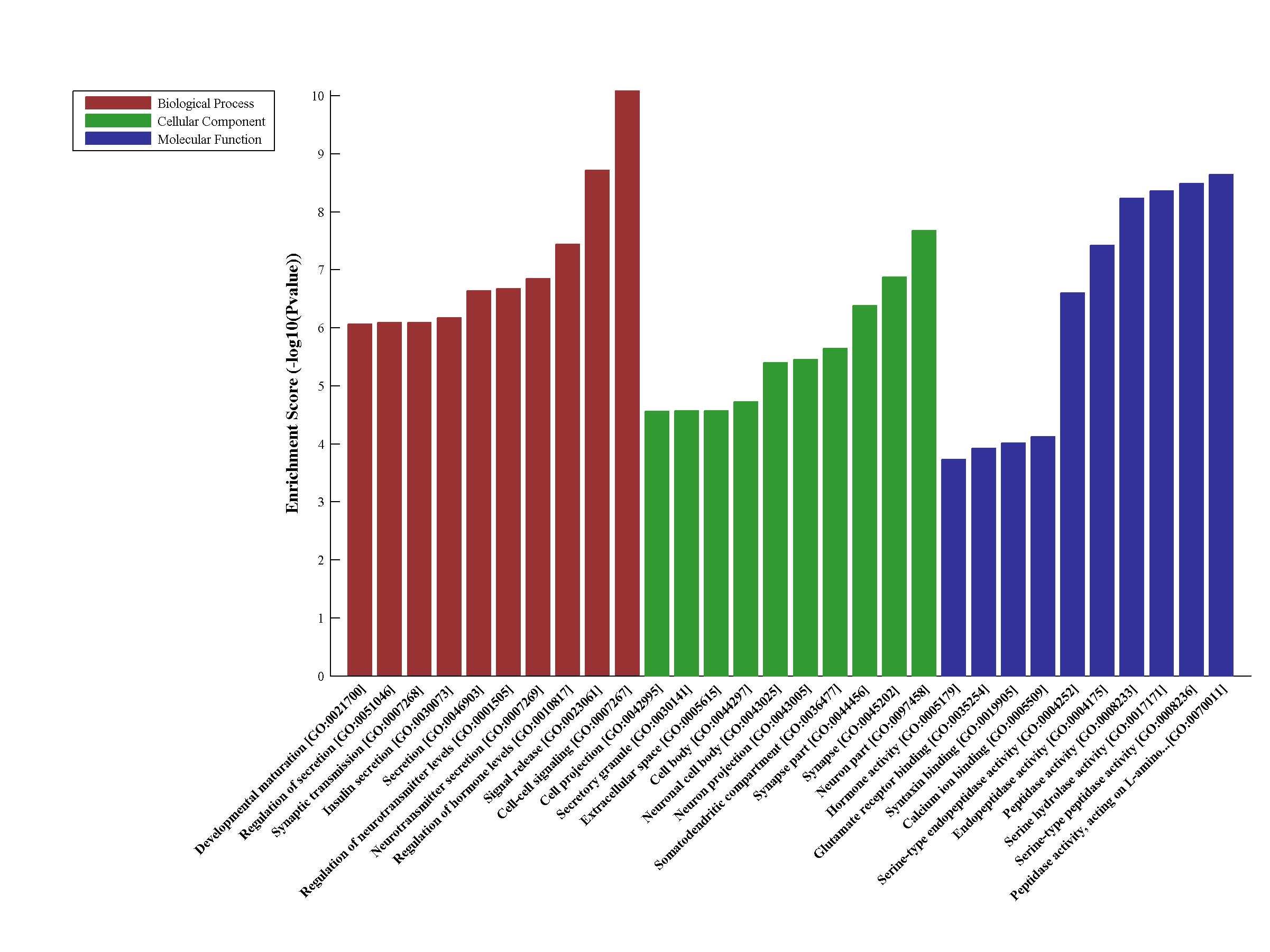

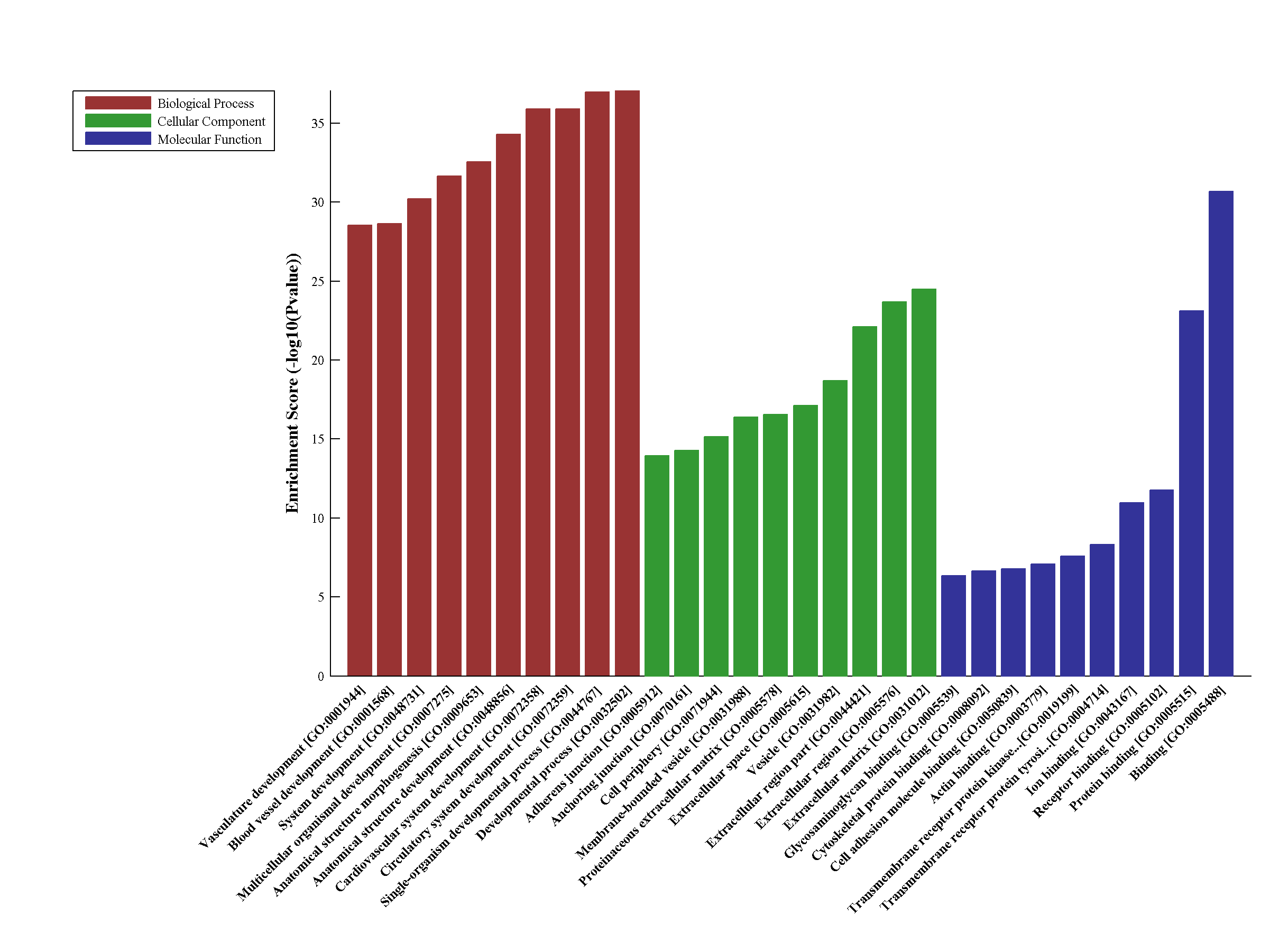


200μg DNA EP group vs TB model group

Q R

**Supplementary Figure 2. GO analyses of significant DE genes between the TB model group and the normal group or each *ag85ab* DNA vaccine group and TB model group.**

Significant DE genes between the mouse TB model group and the normal mouse group or between 10, 50, 100, and 200μg IM groups, or EP groups and mouse TB model group were respectively identified in biological process (BP), cellular component (CC), and molecular function (MF). The X-axis coordinate represents the top 10 items of enrichment scores (sorted by P-value), and the Y-axis represents enrichment scores (scaled by p-value Log10). The P-value represents the enrichment significance of the GO item in the DE gene list, and the smaller the P-value is, the more significant the enrichment is (P≤0.05 is significant). Red columns represent BP, green columns represent CC, and blue columns represent MF.
