# Supplementary material for: Mechanisms of ag85a/b DNA vaccine conferred immunotherapy and recovery from Mycobacterium tuberculosis‐induced injury
Source: Immun Inflamm Dis. 2023 May 16;11(5):e854. doi: 10.1002/iid3.854 (PMC10187016; doi:10.1002/iid3.854)
Supplement: Supplementary file 3 — Supporting information. [file IID3-11-e854-s008.docx]

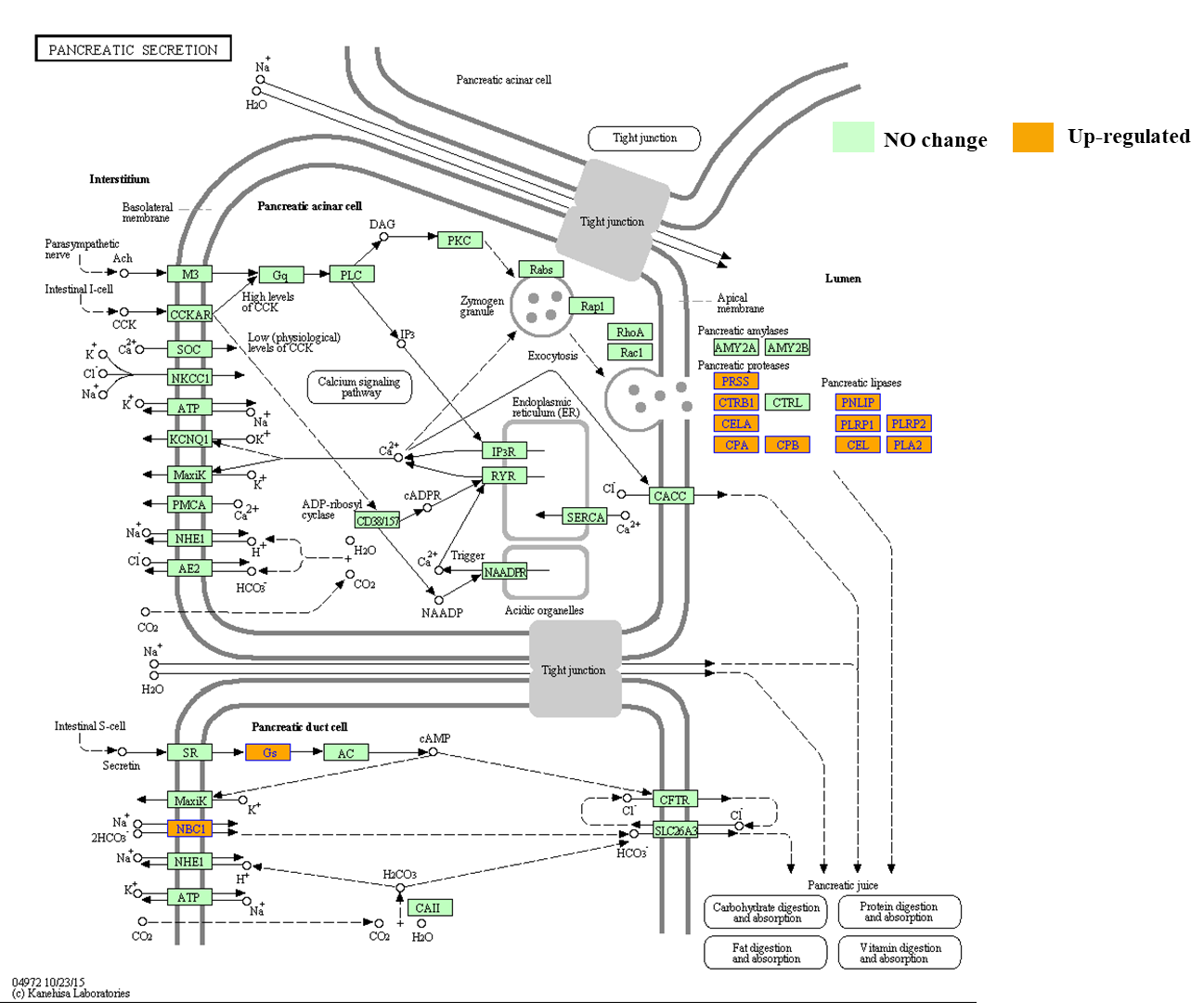


A


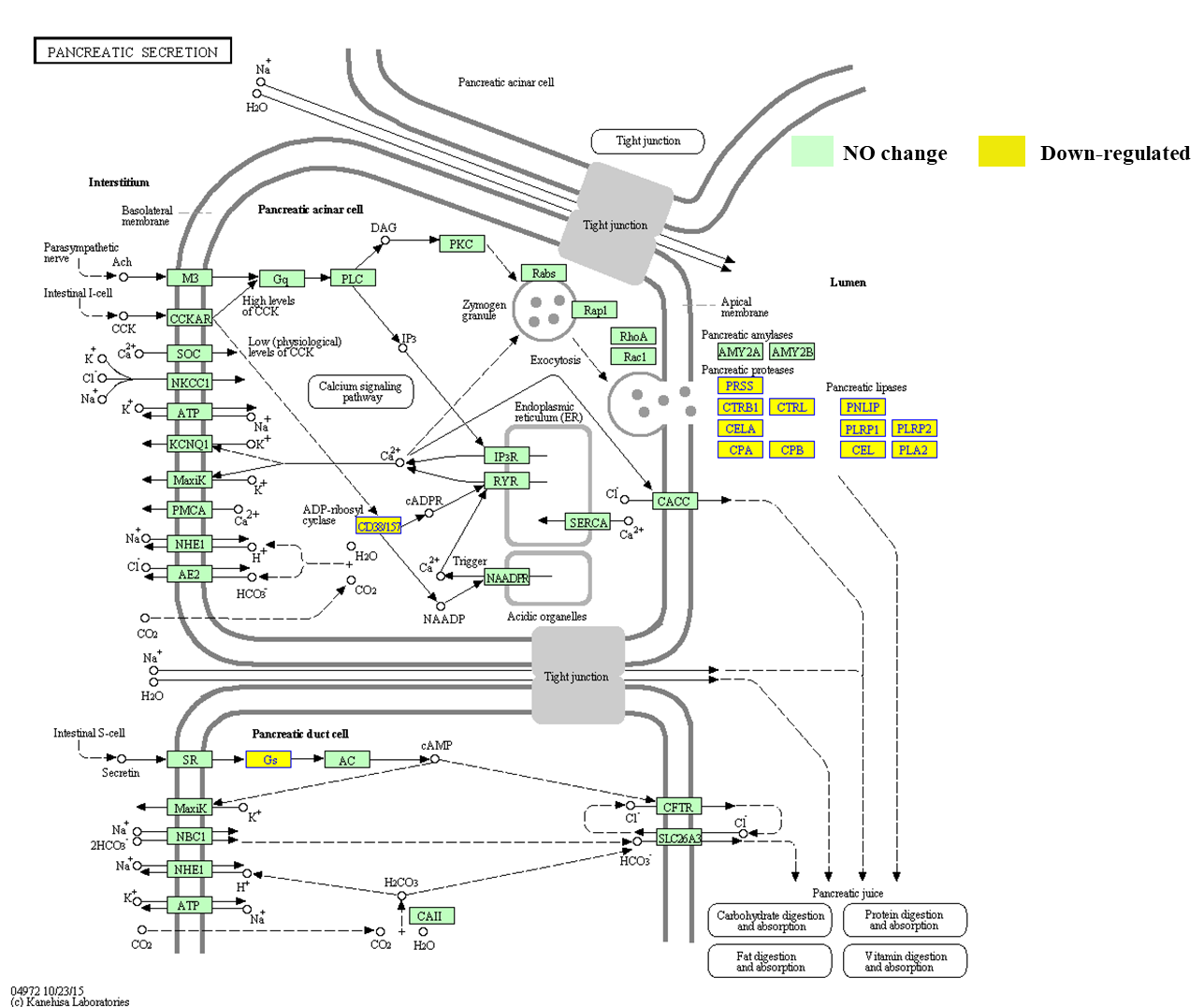


B


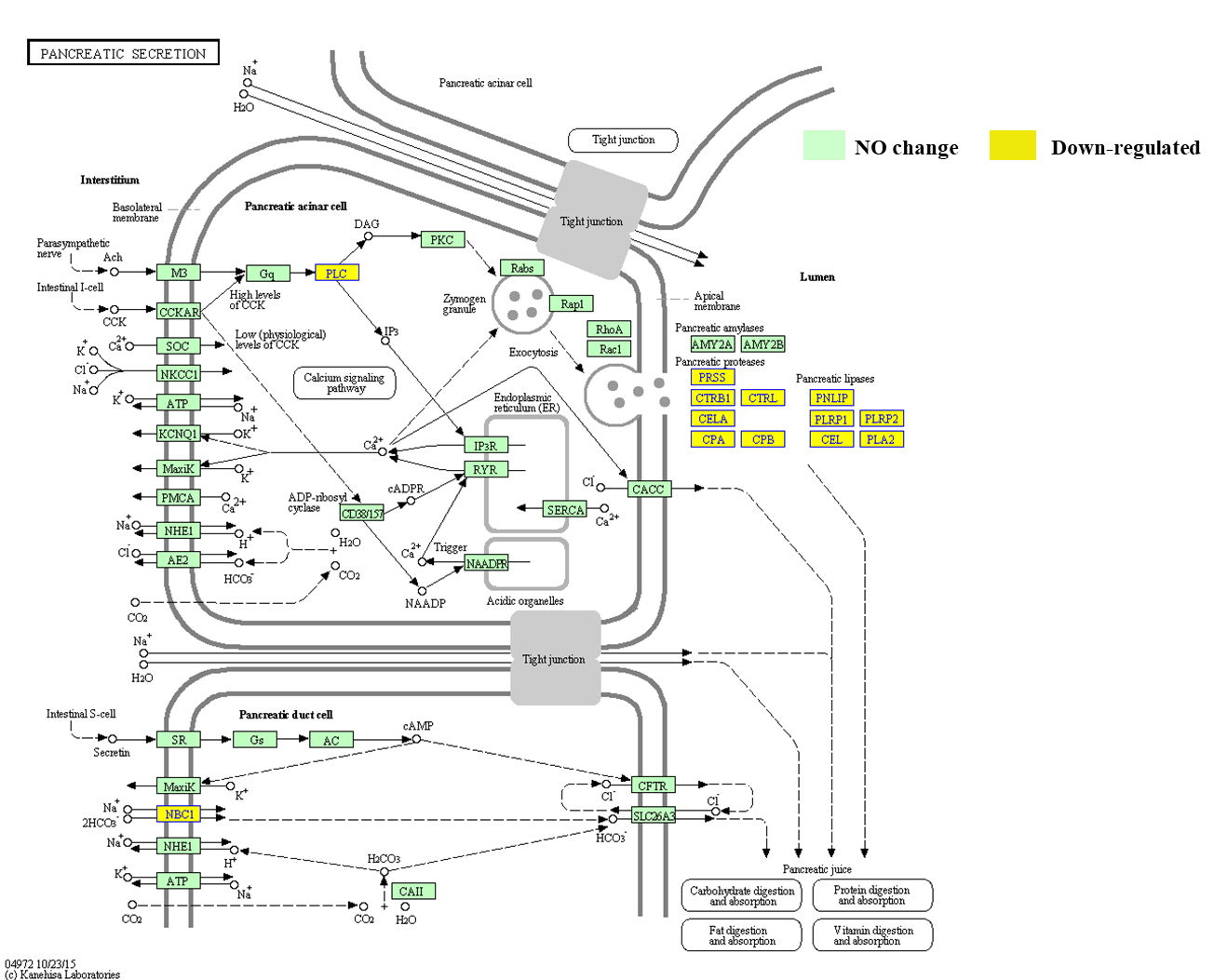


C


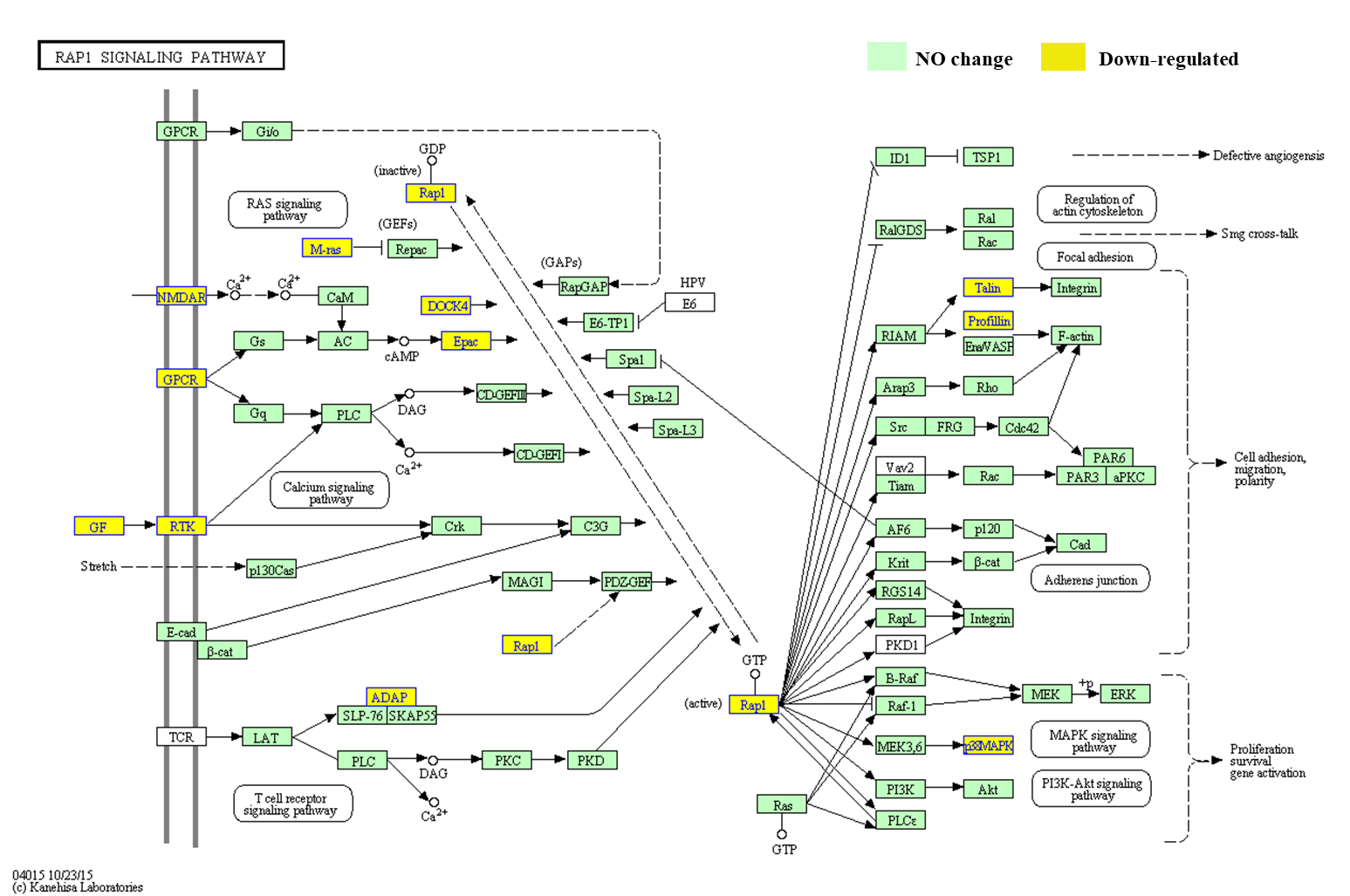


D


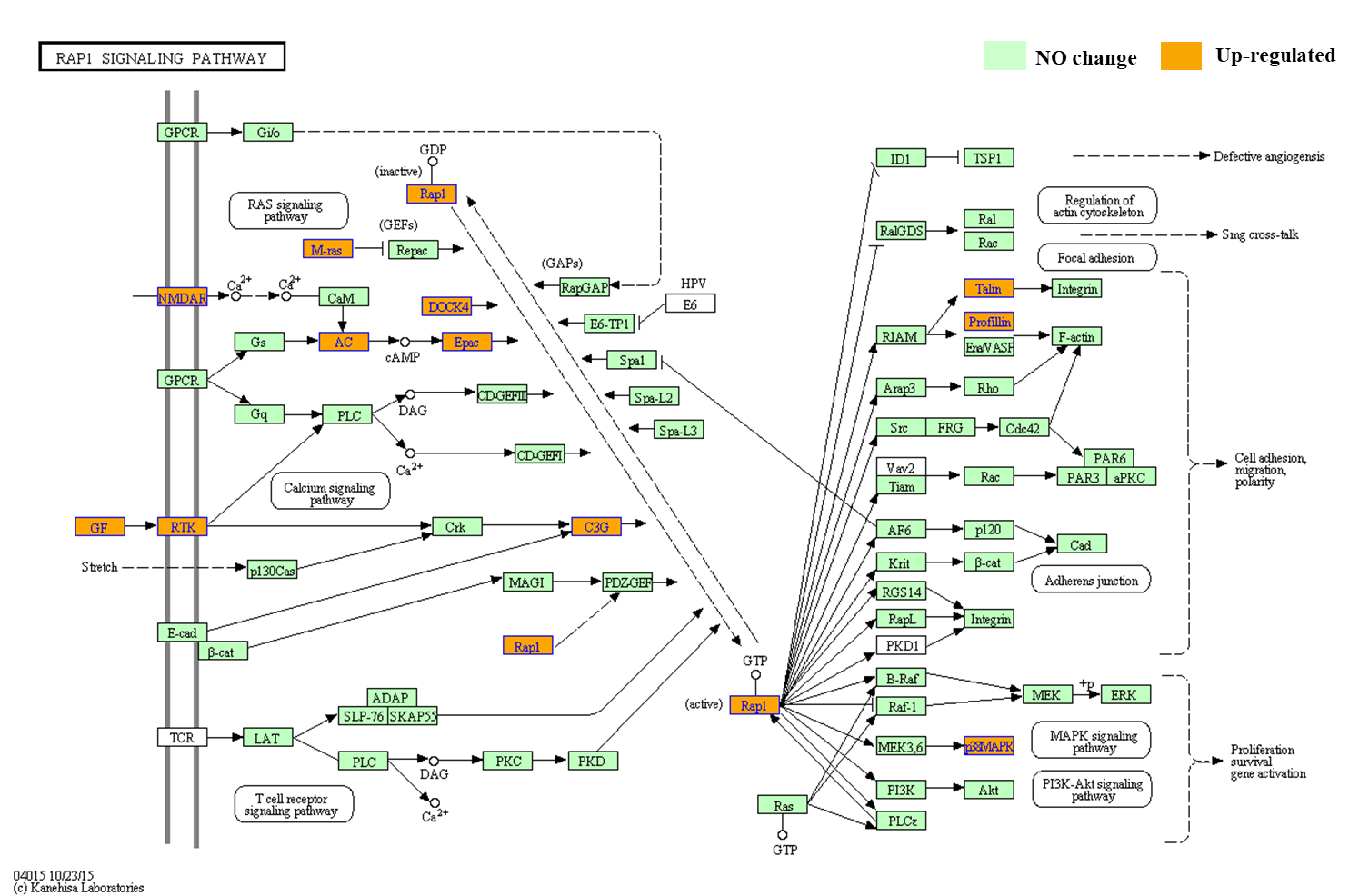


E


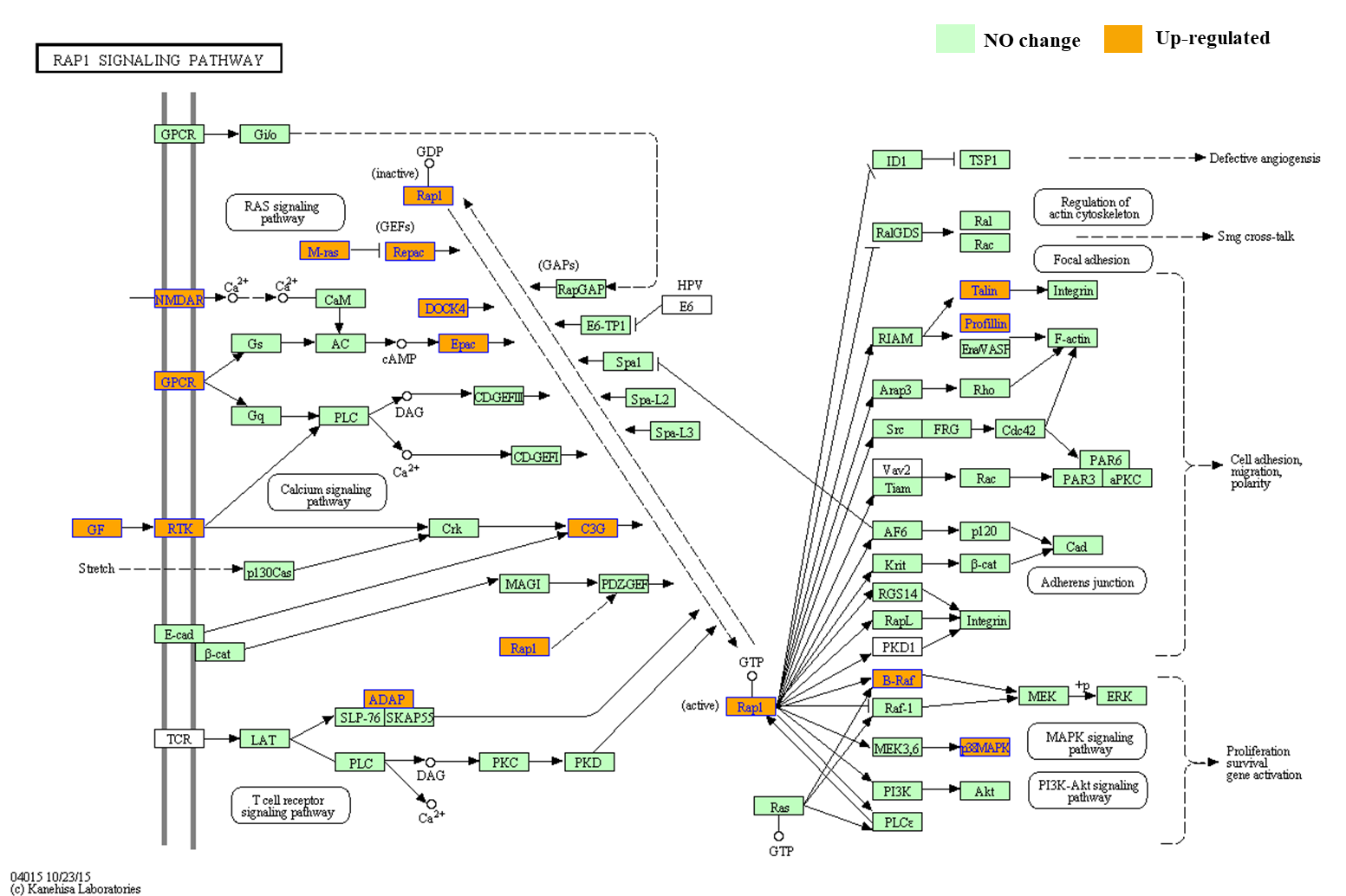


F

**Supplementary Figure 3. The changes of Pancreatic secretion pathway and Rap1 signaling pathway in TB model vs Normal group, 100μg DNA IM vs TB model group and 50μg DNA EP vs TB model group.**

A: Pancreatic secretion pathway in TB model vs Normal group; B: Pancreatic secretion pathway in 100μg DNA IM vs TB model group; C: Pancreatic secretion pathway in 50μg DNA EP vs TB model group; D: Rap1 signaling pathway in TB model vs Normal group; E: Rap1 signaling pathway in 100μg DNA IM vs TB model group; F: Rap1 signaling pathway in 50μg DNA EP vs TB model group
