# Supplementary material for: Mechanisms of ag85a/b DNA vaccine conferred immunotherapy and recovery from Mycobacterium tuberculosis‐induced injury
Source: Immun Inflamm Dis. 2023 May 16;11(5):e854. doi: 10.1002/iid3.854 (PMC10187016; doi:10.1002/iid3.854)
Supplement: Supplementary file 4 — Supporting information. [file IID3-11-e854-s003.docx]

Supplementary Table 1. The top 20 significantly down-regulated DE genes in 100μg *ag85a/b* DNA IM group vs. TB model group and its changes in TB model group vs. normal group and 50μg *ag85a/b* DNA EP group vs. TB model group

| **Genbank Accession** | **Gene Symbol** | **Fold Change value of the DE gene** | | | **Annotation** |
| --- | --- | --- | --- | --- | --- |
|  |  | 100μg DNA IM vs TB model | TB model vs Normal | 50μg DNA EP vs TB model |  |
| NM_010491 | Iapp | 620↓ | 832↑ | 640↓ | Islet amyloid polypeptide, has direct toxicity to islet B cells, mediates local inflammatory reaction, leading to pancreatic islet dysfunction (1-3) , an important pathological factor causing T2DM. |
| NM_007693 | Chga | 433↓ | 326↑ | 342↓ | Chromogranin A, has anti-inflammatory properties and participates in inflammatory reaction, involves in the pathogenesis of diabetes (4, 5). |
| NM_009129 | Scg2 | 391↓ | 428↑ | 345↓ | Secretogranin II, has anti-inflammatory properties and participates in inflammatory reaction, involves in the pathogenesis of diabetes(5, 6). |
| NM_008100 | Gcg | 326↓ | 260↑ | 212↓ | Glucagon, a kind of pleiotropic hormone with metabolic effects secreted by islet α cells, which can promote glycogen decomposition and gluconeogenesis to increase blood sugar, and also can promote fat decomposition and lipid oxidation(7). |
| NM_001003405 | Try5 | 314↓ | 337↑ | 287↓ | Trypsin 5, involved in the hydrolysis of proteins and peptide chains |
| NM_025350 | Cpa1 | 312↓ | 309↑ | 305↓ | Carboxypeptidase A1, cleaves C-terminal branched and aromatic amino acids in dietary proteins |
| NM_011646 | Try4 | 250↓ | 140↑ | 240↓ | Trypsin 4, involved in the hydrolysis of proteins and peptide chains |
| NM_001042711 | Amy2a5 | 239↓ | 365↑ | 272↓ | Amylase alpha 2A, a member of the amylase family, involves in starch digestion and glycogen metabolism(8). |
| NM_001126318 | Gm13011 | 218↓ | 218↑ | 178↓ | Elastase 3A, a member of the elastase family, involved in the hydrolysis of various proteins |
| NM_198627 | Vstm2l | 199↓ | 198↑ | 182↓ | V-set and transmembrane domain containing 2 like, a novel modulator of neuroprotective activity. Overexpression of VSTM2L in a variety of cancer samples regulates IL-4 signaling pathway, mainly enrichs in cell signal transduction, immune response, inflammatory response, calcium binding, etc(9). |
| NM_008386 | Ins1 | 181↓ | 177↑ | 184↓ | Insulin 1, a peptide hormone that plays a critical role in regulating carbohydrate and lipid metabolism |
| NM_009430 | Prss2 | 179↓ | 135↑ | 198↓ | Serine protease 2, a member of the trypsin family of serine proteases, encodes anionic trypsinogen |
| NM_029706 | Cpb1 | 125↓ | 121↑ | 124↓ | Carboxypeptidase B1, which cleaves the C-terminus of lysine or arginine, as a serological marker of acute pancreatitis |
| NM_007919 | Cela2a | 103↓ | 99↑ | 84↓ | Chymotrypsin-like elastase 2A, a member of the elastase family, circulates in plasma, reduces platelet hyperactivation, triggers insulin secretion and degradation, and increases insulin sensitivity(10). |
| NM_007694 | Chgb | 91↓ | 91↑ | 49↓ | Chromogranin B, a neuroendocrine granule protein, and CHGB are involved in the assembly of catecholamine-secreting vesicles and control their ability to secrete in response to nicotine stimulation |
| NM_153518 | Ccdc65 | 87↓ | 163↑ | 154↓ | Coiled-coil domain containing 65, encodes many proteins like motor and skeletal proteins and involved in protein refolding and molecular recognition systems(11). |
| NM_013697 | Ttr | 85↓ | 69↑ | 86↓ | Transthyretin, a homotetrameric carrier protein, transports thyroid hormones in plasma and cerebrospinal fluid, and is also involved in plasma retinol (vitamin A) transport, proteolysis, nerve regeneration, autophagy, and glucose homeostasis |
| NM_009049 | Resp18 | 80↓ | 81↑ | 63↓ | Regulated endocrine specific protein 18, a novel molecule involved in the secretory pathway of neuroendocrine cells from the lumen to the nucleus, and involved in multiple neuroendocrine regulatory pathways |
| NM_053243 | Prss1 | 66↓ | 66↑ | 65↓ | Serine protease 1, a member of the trypsin family of serine proteases, secreted by the pancreas and cleaved into its active form in the small intestine |
| NM_030596 | Dsg3 | 65↓ | 67↑ | 67↓ | Desmoglein 3, also known as Cadherin family member 6 (CDHF6), a member of the Cadherin family, plays a key role in cell-cell adhesion, mainly expressed in lamellar squamous epithelium, including skin, oral mucosa and esophagus |

Supplementary Table 2. The top 20 significantly up-regulated DE genes in 100μg *ag85a/b* DNA IM group vs. TB model group and its changes in TB model group vs. normal group and 50μg *ag85a/b* DNA EP group vs. TB model group

| **Genbank Accession** | **Gene Symbol** | **Fold Change value of the DE gene** | | | **Annotation** |
| --- | --- | --- | --- | --- | --- |
|  |  | 100μg DNA IM vs TB model | TB model vs Normal | 50μg DNA EP vs TB model |  |
| NM_011359 | Sftpc | 559↑ | 167↓ | 289↑ | Surfactant protein C, a hydrophobic protein secreted by alveolar epithelial cells, maintains lung tissue stability by reducing the surface tension of the fluid covering the lungs(12). |
| NM_001282071 | Sftpb | 348↑ | 68↓ | 129↑ | Surfactant protein B, an amphoteric surfactant protein secreted by alveolar epithelial cells, increases the diffusion rate and stability of the surfactant layer in vitro(13). |
| NM_007817 | Cyp2f2 | 166↑ | 22↓ | 48↑ | Cytochrome P450, family 2, subfamily f, polypeptide 2, is a monooxygenase that catalyzes many reactions in drug metabolism and metabolizes a variety of pulmonary toxicants(14). |
| NM_009349 | Inmt | 164↑ | 43↓ | 36↑ | Indolethylamine N-methyltransferase, is a methyltransferase that regulates the N-methylation of tryptamine family proteins, participates in the development and activity of the nervous system(15). |
| NM_009160 | Sftpd | 141↑ | 48↓ | 78↑ | Surfactant protein D, a member of the collectin family, is soluble innate immune molecule which maintain lung homeostasis through their dual roles as anti-infectious and immunomodulatory agents(16, 17). |
| NM_023134 | Sftpa1 | 127↑ | 32↓ | 50↑ | Surfactant protein A1, a member of type C lectin subfamily, plays an important role in surfactant homeostasis and defense against respiratory pathogens, and mediates adhesion and phagocytosis of MTB by alveolar macrophages (18). |
| NM_026323 | Wfdc2 | 117↑ | 32↓ | 73↑ | WAP four-disulfide core domain 2, a small secreted protein that functions as a protease inhibitor, plays critical roles in multiple aspects of lung function, such as promoting mucociliary clearance, conferring anti-inflammatory activity, and reducing surface tension(19). |
| NM_001029937 | Sec14l3 | 116↑ | 28↓ | 86↑ | SEC14-like 3, a 45kDa secretory protein specifically expressed in airway epithelial cells, has a close relationship with airway inflammation, and decreases significantly with the aggravation of airway inflammation, which may be a new marker of airway inflammation(20, 21). |
| NM_001198766 | Postn | 87↑ | 27↓ | 79↑ | Periostin, a 90-kDa secreted extracellular matrix protein, binds to many extracellular matrix proteins through its different domains, and can bind to diverse integrins to activate the TGF-β, PI3K/Akt, Wnt, RhoA/ROCK, NF-κB, MAPK, and JAK pathways(22). |
| NM_010426 | Foxf1 | 86↑ | 17↓ | 39↑ | Forkhead box F1, a mesenchymal transcriptional factor essential for lung development, promotes normal lung homeostasis and repair(23). |
| NM_007621 | Cbr2 | 64↑ | 12↓ | 13↑ | Carbonyl reductase 2, enables carbonyl reductase (NADPH) activity, located in mitochondria and involved in glucose metabolism. |
| NM_007929 | Emp2 | 64↑ | 21↓ | 45↑ | Epithelial membrane protein 2, a member of the tetraspan superfamily of membrane protein, has a variety of functions, including endocytosis, cell signaling, proliferation, migration, and adhesion(24). |
| NM_013657 | Sema3c | 60↑ | 11↓ | 24↑ | Semaphorin 3C, a secreted semaphore, plays a role in the proliferation, apoptosis, adhesion, and migration of various endothelial cells and cancer cells, and promotes the progression of many different cancer types by promoting angiogenesis (25) |
| NM_008344 | Igfbp6 | 58↑ | 16↓ | 15↑ | Insulin-like growth factor binding protein 6, prolongs the half-life of insulin-like growth factor (IGF), inhibits or stimulates the growth-promoting effect of IGF in cell culture, activates the MAPK signaling pathway, and induces cell migration (26) |
| NM_010329 | Pdpn | 57↑ | 32↓ | 66↑ | Podoplanin, a small transmembrane mucin-like glycoprotein that plays an important role in immune response. In different inflammation-related diseases, the expression of Pdpn in immune cells participates in the regulation of inflammation(27). |
| NM_013805 | Cldn5 | 52↑ | 23↓ | 36↑ | Claudin 5, a member of the Claudin family, is an integral membrane protein and a component of tight junctions, which act as physical barriers preventing free passage of solutes and water through the paracellular space between epithelial or endothelial cell sheets. |
| NM_008542 | Smad6 | 50↑ | 12↓ | 13↑ | SMAD family member 6, SMAD protein is a signal transducer and transcriptional regulator that mediates multiple signaling pathways, and this protein plays a role in the negative regulation of BMP and TGF-β signaling pathways |
| NM_011536 | Tbx4 | 46↑ | 15↓ | 27↑ | T-box transcription factor 4, transcriptional regulator with important roles in lung, pelvis, and hindlimb organogenesis |
| NM_011681 | Scgb1a1 | 46↑ | 12↓ | 9↑ | Secretoglobin Family 1A Member 1, implicated in multiple functions including anti-inflammatory, inhibition of phospholipase A2 and the sequestering of hydrophobic ligands |
| NM_181395 | Pxdn | 39↑ | 10↓ | 24↑ | Peroxidasin，a heme-containing peroxidase secreted into the extracellular matrix, involved in extracellular matrix formation, and may play a role in the physiological and pathological fibrotic responses of the fibrotic kidney |

1. Denroche HC, Verchere CB. IAPP and type 1 diabetes: implications for immunity, metabolism and islet transplants. J Mol Endocrinol. 2018;60(2):R57-R75.

2. Masters SL, Dunne A, Subramanian SL, Hull RL, Tannahill GM, Sharp FA, et al. Activation of the NLRP3 inflammasome by islet amyloid polypeptide provides a mechanism for enhanced IL-1beta in type 2 diabetes. Nat Immunol. 2010;11(10):897-904.

3. Morikawa S, Kaneko N, Okumura C, Taguchi H, Kurata M, Yamamoto T, et al. IAPP/amylin deposition, which is correlated with expressions of ASC and IL-1beta in beta-cells of Langerhans' islets, directly initiates NLRP3 inflammasome activation. Int J Immunopathol Pharmacol. 2018;32:2058738418788749.

4. Muntjewerff EM, Dunkel G, Nicolasen MJT, Mahata SK, van den Bogaart G. Catestatin as a Target for Treatment of Inflammatory Diseases. Front Immunol. 2018;9:2199.

5. De Lorenzo R, Sciorati C, Ramirez GA, Colombo B, Lore NI, Capobianco A, et al. Chromogranin A plasma levels predict mortality in COVID-19. PLoS One. 2022;17(4):e0267235.

6. Shooshtarizadeh P, Zhang D, Chich JF, Gasnier C, Schneider F, Haikel Y, et al. The antimicrobial peptides derived from chromogranin/secretogranin family, new actors of innate immunity. Regul Pept. 2010;165(1):102-10.

7. Zeigerer A, Sekar R, Kleinert M, Nason S, Habegger KM, Muller TD. Glucagon's Metabolic Action in Health and Disease. Compr Physiol. 2021;11(2):1759-83.

8. Azzopardi E, Lloyd C, Teixeira SR, Conlan RS, Whitaker IS. Clinical applications of amylase: Novel perspectives. Surgery. 2016;160(1):26-37.

9. Zhang S, Xiong H, Yang J, Yuan X. Pan-Cancer Analysis Reveals the Multidimensional Expression and Prognostic and Immunologic Roles of VSTM2L in Cancer. Front Mol Biosci. 2021;8:792154.

10. Esteghamat F, Broughton JS, Smith E, Cardone R, Tyagi T, Guerra M, et al. CELA2A mutations predispose to early-onset atherosclerosis and metabolic syndrome and affect plasma insulin and platelet activation. Nat Genet. 2019;51(8):1233-43.

11. Deng T, Shen P, Li A, Zhang Z, Yang H, Deng X, et al. CCDC65 as a new potential tumor suppressor induced by metformin inhibits activation of AKT1 via ubiquitination of ENO1 in gastric cancer. Theranostics. 2021;11(16):8112-28.

12. Mulugeta S, Beers MF. Surfactant protein C: its unique properties and emerging immunomodulatory role in the lung. Microbes Infect. 2006;8(8):2317-23.

13. Hawgood S. Surfactant protein B: structure and function. Biol Neonate. 2004;85(4):285-9.

14. Shultz MA, Morin D, Chang AM, Buckpitt A. Metabolic capabilities of CYP2F2 with various pulmonary toxicants and its relative abundance in mouse lung subcompartments. J Pharmacol Exp Ther. 2001;296(2):510-9.

15. Jianfeng W, Yutao W, Jianbin B. Indolethylamine-N-Methyltransferase Inhibits Proliferation and Promotes Apoptosis of Human Prostate Cancer Cells: A Mechanistic Exploration. Front Cell Dev Biol. 2022;10:805402.

16. Haczku A. Protective role of the lung collectins surfactant protein A and surfactant protein D in airway inflammation. J Allergy Clin Immunol. 2008;122(5):861-79; quiz 80-1.

17. Forbes LR, Haczku A. SP-D and regulation of the pulmonary innate immune system in allergic airway changes. Clin Exp Allergy. 2010;40(4):547-62.

18. Gaynor CD, McCormack FX, Voelker DR, McGowan SE, Schlesinger LS. Pulmonary surfactant protein A mediates enhanced phagocytosis of Mycobacterium tuberculosis by a direct interaction with human macrophages. J Immunol. 1995;155(11):5343-51.

19. Nakajima K, Ono M, Radovic U, Dizdarevic S, Tomizawa SI, Kuroha K, et al. Lack of whey acidic protein (WAP) four-disulfide core domain protease inhibitor 2 (WFDC2) causes neonatal death from respiratory failure in mice. Dis Model Mech. 2019;12(11).

20. Shan L, Kawakami T, Asano S, Noritake S, Yoshimoto D, Yamashita K, et al. Inverse relationship between Sec14l3 mRNA/protein expression and allergic airway inflammation. Eur J Pharmacol. 2009;616(1-3):293-300.

21. Shan L, Noritake S, Fujiwara M, Asano S, Yoshida-Noro C, Noro N, et al. Sec14l3 is specifically expressed in mouse airway ciliated cells. Inflammation. 2012;35(2):702-12.

22. Wang Z, An J, Zhu D, Chen H, Lin A, Kang J, et al. Periostin: an emerging activator of multiple signaling pathways. J Cell Commun Signal. 2022;16(4):515-30.

23. Cai Y, Bolte C, Le T, Goda C, Xu Y, Kalin TV, et al. FOXF1 maintains endothelial barrier function and prevents edema after lung injury. Sci Signal. 2016;9(424):ra40.

24. Lin WC, Gowdy KM, Madenspacher JH, Zemans RL, Yamamoto K, Lyons-Cohen M, et al. Epithelial membrane protein 2 governs transepithelial migration of neutrophils into the airspace. J Clin Invest. 2020;130(1):157-70.

25. Hui DHF, Tam KJ, Jiao IZF, Ong CJ. Semaphorin 3C as a Therapeutic Target in Prostate and Other Cancers. Int J Mol Sci. 2019;20(3).

26. Fu P, Yang Z, Bach LA. Prohibitin-2 binding modulates insulin-like growth factor-binding protein-6 (IGFBP-6)-induced rhabdomyosarcoma cell migration. J Biol Chem. 2013;288(41):29890-900.

27. Zhang Z, Zhang N, Yu J, Xu W, Gao J, Lv X, et al. The Role of Podoplanin in the Immune System and Inflammation. J Inflamm Res. 2022;15:3561-72.
