# Supplementary material for: Mechanisms of ag85a/b DNA vaccine conferred immunotherapy and recovery from Mycobacterium tuberculosis‐induced injury
Source: Immun Inflamm Dis. 2023 May 16;11(5):e854. doi: 10.1002/iid3.854 (PMC10187016; doi:10.1002/iid3.854)
Supplement: Supplementary file 5 — Supporting information. [file IID3-11-e854-s007.docx]

Supplementary Table 3 The top 20 significantly down-regulated DE genes in 50μg *ag85a/b* DNA EP group vs. TB model group and its changes in TB model group vs. normal group and 100μg *ag85a/b* DNA IM group vs. TB model group

| **Genbank Accession** | **Gene Symbol** | **Fold Change value of the DE gene** | | | **Annotation** |
| --- | --- | --- | --- | --- | --- |
|  |  | 50μg DNA EP vs TB model | TB model vs Normal | 100μg DNA IM vs TB model |  |
| NM_010491 | Iapp | 640↓ | 832↑ | 620↓ | Islet amyloid polypeptide, has direct toxicity to islet B cells, mediates local inflammatory reaction, leading to pancreatic islet dysfunction [1-3] , an important pathological factor causing T2DM. |
| NM_009129 | Scg2 | 345↓ | 428↑ | 391↓ | Secretogranin II, has anti-inflammatory properties and participates in inflammatory reaction, involves in the pathogenesis of diabetes[4, 5]. |
| NM_007693 | Chga | 342↓ | 326↑ | 433↓ | Chromogranin A, has anti-inflammatory properties and participates in inflammatory reaction, involves in the pathogenesis of diabetes [5, 6]. |
| NM_025350 | Cpa1 | 305↓ | 309↑ | 312↓ | Carboxypeptidase A1, cleaves C-terminal branched and aromatic amino acids in dietary proteins |
| NM_001003405 | Try5 | 287↓ | 337↑ | 314↓ | Trypsin 5, involved in the hydrolysis of proteins and peptide chains |
| NM_001042711 | Amy2a5 | 272↓ | 365↑ | 239↓ | Amylase alpha 2A, a member of the amylase family, involves in starch digestion and glycogen metabolism[7]. |
| NM_011646 | Try4 | 240↓ | 140↑ | 250↓ | Trypsin 4, involved in the hydrolysis of proteins and peptide chains |
| NM_008100 | Gcg | 212↓ | 260↑ | 326↓ | Glucagon, a kind of pleiotropic hormone with metabolic effects secreted by islet α cells, which can promote glycogen decomposition and gluconeogenesis to increase blood sugar, and also can promote fat decomposition and lipid oxidation[8]. |
| NM_009430 | Prss2 | 198↓ | 135↑ | 179↓ | Serine protease 2, a member of the trypsin family of serine proteases, encodes anionic trypsinogen |
| NM_008386 | Ins1 | 184↓ | 177↑ | 181↓ | Insulin 1, a peptide hormone that plays a critical role in regulating carbohydrate and lipid metabolism |
| NM_198627 | Vstm2l | 182↓ | 198↑ | 199↓ | V-set and transmembrane domain containing 2 like, a novel modulator of neuroprotective activity. Overexpression of VSTM2L in a variety of cancer samples regulates IL-4 signaling pathway, mainly enrichs in cell signal transduction, immune response, inflammatory response, calcium binding, etc[9]. |
| NM_001126318 | Gm13011 | 178↓ | 218↑ | 218↓ | Elastase 3A, a member of the elastase family, involved in the hydrolysis of various proteins |
| NM_153518 | Ccdc65 | 154↓ | 163↑ | 87↓ | Coiled-coil domain containing 65, encodes many proteins like motor and skeletal proteins and involved in protein refolding and molecular recognition systems[10]. |
| NM_029706 | Cpb1 | 124↓ | 121↑ | 125↓ | Carboxypeptidase B1, which cleaves the C-terminus of lysine or arginine, as a serological marker of acute pancreatitis |
| NM_026925 | Pnlip | 111↓ | 112↑ | 60↓ | Pancreatic lipase, a member of the lipase family, secreted by the pancreas, hydrolyzes dietary triglycerides in the small intestine[11]. |
| NM_013697 | Ttr | 86↓ | 69↑ | 85↓ | Thyroid hormone carrier protein, a homologous tetramer carrier protein, transports thyroid hormone in plasma and cerebrospinal fluid, and also participates in the transport of retinol (vitamin A) in plasma, proteolysis, nerve regeneration, autophagy and glucose homeostasis |
| NM_007919 | Cela2a | 84↓ | 99↑ | 103↓ | Chymotrypsin-like elastase 2A, a member of the elastase family, circulates in plasma, reduces platelet hyperactivation, triggers insulin secretion and degradation, and increases insulin sensitivity[12]. |
| NM_172816 | Slc30a8 | 80↓ | 78↑ | 58↓ | Solute carrier family 30 member 8, a zinc transporter, transports Zinc from cytoplasm to insulin secretory granules in the pancreatic beta-cells(51). |
| NM_030596 | Dsg3 | 67↓ | 67↑ | 65↓ | Desmoglein 3, also known as Cadherin family member 6 (CDHF6), is a member of the connectome Cadherin family and plays a key role in cell-cell adhesion. It is mainly expressed in stratified squamous epithelium, including skin, oral mucosa and esophagus. |
| NM_053243 | Prss1 | 65↓ | 66↑ | 66↓ | Serine protease 1, a member of the trypsin family that is secreted by the pancreas and splits into its active form in the small intestine |

Supplementary Table 4 The top 20 significantly up-regulated DE genes in 50μg *ag85a/b* DNA EP group vs. TB model group and its changes in TB model group vs. normal group and 100μg *ag85a/b* DNA IM group vs. TB model group

| **Genbank Accession** | **Gene Symbol** | **Fold Change value of the DE gene** | | | **Annotation** |
| --- | --- | --- | --- | --- | --- |
|  |  | 50μg DNA EP vs TB model | TB model vs Normal | 100μg DNA IM vs TB model |  |
| NM_011359 | Sftpc | 289↑ | 167↓ | 559↑ | Surfactant protein C, a hydrophobic protein secreted by alveolar epithelial cells, maintains lung tissue stability by reducing the surface tension of the fluid covering the lungs(89). |
| NM_001282071 | Sftpb | 129↑ | 68↓ | 348↑ | Surfactant protein B, an amphoteric surfactant protein secreted by alveolar epithelial cells, increases the diffusion rate and stability of the surfactant layer in vitro(90). |
| NM_020509 | Retnla | 95↑ | 21↓ | 24↑ | Resistin-like alpha, a member of the resistin family, is a secreted cysteine-rich protein with insulin resistance and also an anti-inflammatory marker of macrophages(78, 79). |
| NM_001029937 | Sec14l3 | 86↑ | 28↓ | 116↑ | SEC14-like 3, a 45kDa secretory protein specifically expressed in airway epithelial cells, has a close relationship with airway inflammation, and decreases significantly with the aggravation of airway inflammation, which may be a new marker of airway inflammation(96, 97). |
| NM_001198766 | Postn | 79↑ | 27↓ | 87↑ | Periostin, a 90-kDa secreted extracellular matrix protein, binds to many extracellular matrix proteins through its different domains, and can bind to diverse integrins to activate the TGF-β, PI3K/Akt, Wnt, RhoA/ROCK, NF-κB, MAPK, and JAK pathways(98). |
| NM_009160 | Sftpd | 78↑ | 48↓ | 141↑ | Surfactant protein D, involved in surfactant metabolism of inhaled microorganisms and chemicals, also involved in the innate immune response, protects the lungs from inhaled microorganisms and chemicals [13, 14] |
| NM_026323 | Wfdc2 | 73↑ | 32↓ | 117↑ | WAP four-disulfide core domain 2, a small secreted protein that functions as protease inhibitors, such as serine-type endopeptidase inhibitor activity and cysteine-type endopeptidase Inhibitor activity |
| NM_010329 | Pdpn | 66↑ | 32↓ | 57↑ | Podoplanin, a transmembrane sialoglycoprotein with different distributions in different tissues, is significantly expressed in various malignant tumor cells, and involved in the membrane transport of amino acids and folic acid |
| NM_011315 | Saa3 | 56↑ | 20↓ | NO | Serum amyloid A3, a pseudogene in humans, is a major component of acute-phase inflammation, serves as an endogenous peptide ligand for TLR4, and binds MD-2 to activate p38 and NF-κB pathways in a MyD88-dependent manner(102). |
| NM_023134 | Sftpa1 | 50↑ | 32↓ | 127↑ | Surfactant protein A1, a member of type C lectin subfamily, plays an important role in surfactant homeostasis and defense against respiratory pathogens, and mediates adhesion and phagocytosis of MTB by alveolar macrophages [15] |
| NM_010217 | Ctgf | 50↑ | 15↓ | 31↑ | Connective tissue growth factor, a 38-kDa protein with a tetramodular structure, is involved in the basic lung development process and promotes lung fibroblast proliferation, migration, and differentiation(107). |
| NM_008597 | Mgp | 48↑ | 17↓ | 32↑ | Matrix gla protein, a vitamin K-dependent inhibitor of calcification, may play an anti-inflammatory role in monocytes and macrophages(105). |
| NM_007817 | Cyp2f2 | 48↑ | 22↓ | 166↑ | Cytochrome P450, family 2, subfamily f, polypeptide 2, is a monooxygenase that catalyzes many reactions in drug metabolism and metabolizes a variety of pulmonary toxicants(99). |
| NM_007929 | Emp2 | 45↑ | 21↓ | 64↑ | Epithelial membrane protein 2, a member of the tetraspan superfamily of membrane protein, has a variety of functions, including endocytosis, cell signaling, proliferation, migration, and adhesion(101). |
| NM_008047 | Fstl1 | 44↑ | 11↓ | 27↑ | Follistatin like 1, a dual regulator promoting cardiomyocyte proliferation and fibrosis |
| NM_010426 | Foxf1 | 39↑ | 17↓ | 86↑ | Forkhead box F1, a mesenchymal transcriptional factor essential for lung development, promotes normal lung homeostasis and repair(103). |
| NM_173864 | Gm5077 | 36↑ | 8↓ | 24↑ | - |
| NM_009349 | Inmt | 36↑ | 43↓ | 164↑ | Indoleamine N-methyltransferase, which can N-methylate indole substances such as tryptamine to degrade metabolism |
| NM_013805 | Cldn5 | 36↑ | 23↓ | 52↑ | Claudin 5, a member of the Claudin family, is an integral membrane protein and a component of tight junctions, which act as physical barriers preventing free passage of solutes and water through the paracellular space between epithelial or endothelial cell sheets. |
| NM_010171 | F3 | 29↑ | NO | 15↑ | Coagulation factor III, a cell surface glycoprotein, enables cells to initiate coagulation cascade reaction and acts as a high affinity receptor of coagulation factor VII |

1. Denroche, H.C. and C.B. Verchere, *IAPP and type 1 diabetes: implications for immunity, metabolism and islet transplants.* J Mol Endocrinol, 2018. **60**(2): p. R57-R75.

2. Masters, S.L., et al., *Activation of the NLRP3 inflammasome by islet amyloid polypeptide provides a mechanism for enhanced IL-1beta in type 2 diabetes.* Nat Immunol, 2010. **11**(10): p. 897-904.

3. Morikawa, S., et al., *IAPP/amylin deposition, which is correlated with expressions of ASC and IL-1beta in beta-cells of Langerhans' islets, directly initiates NLRP3 inflammasome activation.* Int J Immunopathol Pharmacol, 2018. **32**: p. 2058738418788749.

4. Shooshtarizadeh, P., et al., *The antimicrobial peptides derived from chromogranin/secretogranin family, new actors of innate immunity.* Regul Pept, 2010. **165**(1): p. 102-10.

5. De Lorenzo, R., et al., *Chromogranin A plasma levels predict mortality in COVID-19.* PLoS One, 2022. **17**(4): p. e0267235.

6. Muntjewerff, E.M., et al., *Catestatin as a Target for Treatment of Inflammatory Diseases.* Front Immunol, 2018. **9**: p. 2199.

7. Azzopardi, E., et al., *Clinical applications of amylase: Novel perspectives.* Surgery, 2016. **160**(1): p. 26-37.

8. Zeigerer, A., et al., *Glucagon's Metabolic Action in Health and Disease.* Compr Physiol, 2021. **11**(2): p. 1759-1783.

9. Zhang, S., et al., *Pan-Cancer Analysis Reveals the Multidimensional Expression and Prognostic and Immunologic Roles of VSTM2L in Cancer.* Front Mol Biosci, 2021. **8**: p. 792154.

10. Deng, T., et al., *CCDC65 as a new potential tumor suppressor induced by metformin inhibits activation of AKT1 via ubiquitination of ENO1 in gastric cancer.* Theranostics, 2021. **11**(16): p. 8112-8128.

11. Hegele, R.A., et al., *Polymorphisms in PNLIP, encoding pancreatic lipase, and associations with metabolic traits.* J Hum Genet, 2001. **46**(6): p. 320-4.

12. Esteghamat, F., et al., *CELA2A mutations predispose to early-onset atherosclerosis and metabolic syndrome and affect plasma insulin and platelet activation.* Nat Genet, 2019. **51**(8): p. 1233-1243.

13. Haczku, A., *Protective role of the lung collectins surfactant protein A and surfactant protein D in airway inflammation.* J Allergy Clin Immunol, 2008. **122**(5): p. 861-79; quiz 880-1.

14. Forbes, L.R. and A. Haczku, *SP-D and regulation of the pulmonary innate immune system in allergic airway changes.* Clin Exp Allergy, 2010. **40**(4): p. 547-62.

15. Gaynor, C.D., et al., *Pulmonary surfactant protein A mediates enhanced phagocytosis of Mycobacterium tuberculosis by a direct interaction with human macrophages.* J Immunol, 1995. **155**(11): p. 5343-51.
