# Supplementary material for: Mechanisms of ag85a/b DNA vaccine conferred immunotherapy and recovery from Mycobacterium tuberculosis‐induced injury
Source: Immun Inflamm Dis. 2023 May 16;11(5):e854. doi: 10.1002/iid3.854 (PMC10187016; doi:10.1002/iid3.854)
Supplement: Supplementary file 6 — Supporting information. [file IID3-11-e854-s002.docx]

Supplementary Table 5 The top 20 significantly down-regulated pathways in 100μg *ag85a/b* DNA IM group vs. TB model group and their changes in TB model group and 50μg *ag85a/b* DNA EP group vs. TB model group

| **Pathway ID** | **Definition** | **Enrichment Score of the pathway** | | |
| --- | --- | --- | --- | --- |
|  |  | 100μg DNA IM vs TB model | TB model vs Normal | 50μg DNA EP vs TB model |
| mmu04972 | Pancreatic secretion | 13.494424↓ | 11.335708 | 13.102152↓ |
| mmu04974 | Protein digestion and absorption | 9.228974↓ | 6.798788 | 8.944734↓ |
| mmu04950 | Maturity onset diabetes of the young | 5.934764↓ | 3.101886 | 4.619483↓ |
| mmu04975 | Fat digestion and absorption | 4.715005↓ | 4.937938 | 4.584038↓ |
| mmu04614 | Renin-angiotensin system | 4.057283↓ | 1.804049 | 2.121122↓ |
| mmu04911 | Insulin secretion | 4.053136↓ | 3.887882 | 4.676213 |
| mmu04080 | Neuroactive ligand-receptor interaction | 3.895453↓ | 3.434282 | 3.649268 |
| mmu04961 | Endocrine and other factor-regulated calcium reabsorption | 3.847204↓ | 1.772715 | 2.897346 |
| mmu05032 | Morphine addiction | 2.423023↓ | 1.825693 | NO |
| mmu05030 | Cocaine addiction | 2.414299↓ | 2.644894 | 1.619297 |
| mmu04930 | Type II diabetes mellitus | 2.376016↓ | NO | 1.590557 |
| mmu04917 | Prolactin signaling pathway | 2.299486↓ | 2.354739 | 2.202203 |
| mmu04713 | Circadian entrainment | 2.297331↓ | 2.7979 | 2.188517 |
| mmu04970 | Salivary secretion | 2.215196↓ | 2.2593 | 1.5306 |
| mmu04723 | Retrograde endocannabinoid signaling | 2.180025↓ | NO | NO |
| mmu04913 | Ovarian steroidogenesis | 2.100693↓ | NO | 2.017946 |
| mmu04728 | Dopaminergic synapse | 2.070013↓ | 1.883599 | 1.486645 |
| mmu00561 | Glycerolipid metabolism | 2.069674↓ | 2.924039 | 1.987283 |
| mmu04066 | HIF-1 signaling pathway | 2.049171↓ | NO | 1.441163 |
| mmu04727 | GABAergic synapse | 1.917068↓ | NO | NO |

Supplementary Table 6 The top 20 significantly up-regulated pathways in 100μg *ag85a/b* DNA IM group vs. TB model group and their changes in TB model group and 50μg *ag85a/b* DNA EP group vs. TB model group

| **Pathway ID** | **Definition** | **Enrichment Score of the pathway** | | |
| --- | --- | --- | --- | --- |
|  |  | 100μg DNA IM vs TB model | TB model vs Normal | 50μg DNA EP vs TB model |
| mmu04512 | ECM-receptor interaction | 8.116145↑ | 2.574761↓ | 7.645445↑ |
| mmu04510 | Focal adhesion | 6.102566↑ | 1.881851↓ | 8.554973↑ |
| mmu04974 | Protein digestion and absorption | 6.066787↑ | 3.682461↓ | 5.672109↑ |
| mmu04610 | Complement and coagulation cascades | 5.643564↑ | 2.391915↓ | 4.144754↑ |
| mmu05205 | Proteoglycans in cancer | 4.623089↑ | NO | 4.624056↑ |
| mmu04151 | PI3K-Akt signaling pathway | 4.191528↑ | 1.526425↓ | 4.990313↑ |
| mmu04933 | AGE-RAGE signaling pathway in diabetic complications | 4.077841↑ | 2.609451↓ | 4.263249↑ |
| mmu00982 | Drug metabolism - cytochrome P450 | 3.331959↑ | 1.951735↓ | 2.742079↑ |
| mmu05200 | Pathways in cancer | 3.270596↑ | NO | 2.748116↑ |
| mmu04015 | Rap1 signaling pathway | 3.119349↑ | 1.39725↓ | 2.822574↑ |
| mmu05204 | Chemical carcinogenesis | 2.997822↑ | NO | NO |
| mmu00980 | Metabolism of xenobiotics by cytochrome P450 | 2.886266↑ | 2.042694↓ | 1.8995↑ |
| mmu00590 | Arachidonic acid metabolism | 2.655805↑ | 2.263825↓ | NO |
| mmu05150 | Staphylococcus aureus infection | 2.522364↑ | 2.220857↓ | 2.605515↑ |
| mmu04360 | Axon guidance | 2.442363↑ | 2.756632↓ | 3.325582↑ |
| mmu05133 | Pertussis | 2.424137↑ | 4.154819↓ | 2.846166↑ |
| mmu05217 | Basal cell carcinoma | 2.35262↑ | NO | NO |
| mmu05146 | Amoebiasis | 2.30181↑ | 1.914775↓ | 2.893891↑ |
| mmu00480 | Glutathione metabolism | 2.299118↑ | 1.550779↓ | NO |
| mmu05414 | Dilated cardiomyopathy | 2.244087↑ | NO | 1.389668↑ |
