# Supplementary material for: Mechanisms of ag85a/b DNA vaccine conferred immunotherapy and recovery from Mycobacterium tuberculosis‐induced injury
Source: Immun Inflamm Dis. 2023 May 16;11(5):e854. doi: 10.1002/iid3.854 (PMC10187016; doi:10.1002/iid3.854)
Supplement: Supplementary file 7 — Supporting information. [file IID3-11-e854-s004.docx]

Supplementary Table 7 The top 20 significantly down-regulated pathways in 50μg *ag85a/b* DNA IM group vs. TB model group and their changes in TB model group and 100μg *ag85a/b* DNA EP group vs. TB model group

| **Pathway ID** | **Definition** | **Enrichment Score of the pathway** | | |
| --- | --- | --- | --- | --- |
|  |  | 50μg DNA EP vs TB model | TB model vs Normal | 100μg DNA IM vs TB model |
| mmu04972 | Pancreatic secretion | 13.102152↓ | 11.335708↑ | 13.494424↓ |
| mmu04974 | Protein digestion and absorption | 8.944734↓ | 6.798788↑ | 9.228974↓ |
| mmu04911 | Insulin secretion | 4.676213↓ | 3.887882↑ | 4.053136↓ |
| mmu04950 | Maturity onset diabetes of the young | 4.619483↓ | 3.101886↑ | 5.934764↓ |
| mmu04975 | Fat digestion and absorption | 4.584038↓ | 4.937938↑ | 4.715005↓ |
| mmu04080 | Neuroactive ligand-receptor interaction | 3.649268↓ | 3.434282↑ | 3.895453↓ |
| mmu04961 | Endocrine and other factor-regulated calcium reabsorption | 2.897346↓ | 1.772715↑ | 3.847204↓ |
| mmu04917 | Prolactin signaling pathway | 2.202203↓ | 2.354739↑ | 2.299486↓ |
| mmu04713 | Circadian entrainment | 2.188517↓ | 2.7979↑ | 2.297331↓ |
| mmu04614 | Renin-angiotensin system | 2.121122↓ | 1.804049↑ | 4.057283↓ |
| mmu04913 | Ovarian steroidogenesis | 2.017946↓ | NO | 2.100693↓ |
| mmu00561 | Glycerolipid metabolism | 1.987283↓ | 2.924039↑ | 2.069674↓ |
| mmu04912 | GnRH signaling pathway | 1.847607↓ | 1.949105↑ | NO |
| mmu04973 | Carbohydrate digestion and absorption | 1.742254↓ | 1.443016↑ | 1.809373↓ |
| mmu04750 | Inflammatory mediator regulation of TRP channels | 1.629171↓ | NO | NO |
| mmu05030 | Cocaine addiction | 1.619297↓ | 2.644894↑ | 2.414299↓ |
| mmu04930 | Type II diabetes mellitus | 1.590557↓ | NO | 2.376016↓ |
| mmu04010 | MAPK signaling pathway | 1.559062↓ | 2.327035↑ | NO |
| mmu04970 | Salivary secretion | 1.5306↓ | 2.2593↑ | 2.215196↓ |
| mmu00514 | Other types of O-glycan biosynthesis | 1.496797↓ | NO | NO |

Supplementary Table 8 The top 20 significantly down-regulated pathways in 50μg *ag85a/b* DNA IM group vs. TB model group and their changes in TB model group and 100μg *ag85a/b* DNA EP group vs. TB model group

| **Pathway ID** | **Definition** | **Enrichment Score of the pathway** | | |
| --- | --- | --- | --- | --- |
|  |  | 50μg DNA EP vs TB model | TB model vs Normal | 100μg DNA IM vs TB model |
| mmu04510 | Focal adhesion | 8.554973↑ | 1.881851↓ | 6.102566↑ |
| mmu04512 | ECM-receptor interaction | 7.645445↑ | 2.574761↓ | 8.116145↑ |
| mmu04974 | Protein digestion and absorption | 5.672109↑ | 3.682461↓ | 6.066787↑ |
| mmu04060 | Cytokine-cytokine receptor interaction | 5.027105↑ | 6.389233↓ | 1.422668↑ |
| mmu04151 | PI3K-Akt signaling pathway | 4.990313↑ | 1.526425↓ | 4.191528↑ |
| mmu05205 | Proteoglycans in cancer | 4.624056↑ | NO | 4.623089↑ |
| mmu04933 | AGE-RAGE signaling pathway in diabetic complications | 4.263249↑ | 2.609451↓ | 4.077841↑ |
| mmu04610 | Complement and coagulation cascades | 4.144754↑ | 2.391915↓ | 5.643564↑ |
| mmu04360 | Axon guidance | 3.325582↑ | 2.756632↓ | 2.442363↑ |
| mmu04014 | Ras signaling pathway | 2.986947↑ | 1.337103↓ | 2.061538↑ |
| mmu05146 | Amoebiasis | 2.893891↑ | 1.914775↓ | 2.30181↑ |
| mmu05133 | Pertussis | 2.846166↑ | 4.154819↓ | 2.424137↑ |
| mmu04015 | Rap1 signaling pathway | 2.822574↑ | 1.39725↓ | 3.119349↑ |
| mmu05200 | Pathways in cancer | 2.748116↑ | NO | 3.270596↑ |
| mmu00982 | Drug metabolism - cytochrome P450 | 2.742079↑ | 1.951735↓ | 3.331959↑ |
| mmu05150 | Staphylococcus aureus infection | 2.605515↑ | 2.220857↓ | 2.522364↑ |
| mmu04350 | TGF-beta signaling pathway | 2.298151↑ | 1.656492↓ | 1.568223↑ |
| mmu04012 | ErbB signaling pathway | 2.211183↑ | NO | 1.506401↑ |
| mmu05410 | Hypertrophic cardiomyopathy (HCM) | 1.928036↑ | NO | 2.005368↑ |
| mmu00980 | Metabolism of xenobiotics by cytochrome P450 | 1.8995↑ | 2.042694↓ | 2.886266↑ |
